# Hit-to-lead optimization of a benzene sulfonamide series for potential antileishmanial agents†

**DOI:** 10.1039/d0md00165a

**Published:** 2020-08-25

**Authors:** Paul J. Koovits, Marco A. Dessoy, An Matheeussen, Louis Maes, Guy Caljon, Leonardo L. G. Ferreira, Rafael C. Chelucci, Simone Michelan-Duarte, Adriano D. Andricopulo, Simon Campbell, Jadel M. Kratz, Charles E. Mowbray, Luiz C. Dias

**Affiliations:** Institute of Chemistry, University of Campinas (UNICAMP) Rua Josué de Castro, S/N, Cidade Universitária Campinas SP 13083-861 Brazil ldias@iqm.unicamp.br +55 19 3521 3097; Laboratory of Microbiology, Parasitology and Hygiene (LMPH), University of Antwerp Universiteitsplein 1 2610 Wilrijk Belgium; Laboratory of Medicinal and Computational Chemistry, Physics Institute of Sao Carlos, University of Sao Paulo Av. Joao Dagnone 1100 13563-120 Sao Carlos-SP Brazil; Drugs for Neglected Diseases initiative (DNDi) 15 Chemin Louis-Dunant 1202 Geneva Switzerland

## Abstract

A series of benzene sulphonamides with good potency and selectivity against *Leishmania* spp. intracellular amastigotes was identified by high-throughput screening. Approximately 200 compounds were synthesized as part of a hit-to-lead optimization program. The potency of the series appears to be strongly dependent on lipophilicity, making the identification of suitable orally available candidates challenging due to poor pharmacokinetics. Despite not identifying a clinical candidate, a likely solvent exposed area was found, best exemplified in compound **29**. Ongoing detailed mode-of-action studies may provide an opportunity to use target-based medicinal chemistry to overcome the issues with the current series.

## Abstract 2

A series of benzene sulphonamides with good potency and selectivity against *Leishmania* spp. intracellular amastigotes was identified by high-throughput screening.

## Graphical abstract

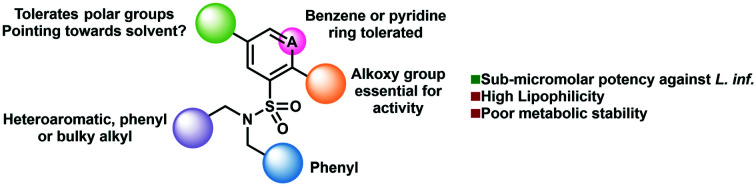

Leishmaniasis is a neglected parasitic protozoal disease that causes an estimated 20 000–30 000 deaths annually and infects up to 12 million people, leaving up to a billion at risk across Latin America, Africa, Asia and the Middle East.^1,2^ The disease is caused by the kinetoplastid *Leishmania*,^3^ of which there are over 20 species, and is transmitted by the female phlebotomine sand fly. There are several clinical forms, the most common being cutaneous leishmaniasis (CL) and visceral leishmaniasis (VL). Annually, there are an estimated 700 000–1 200 000 new cases of CL,^4^ which presents itself as skin lesions on exposed body parts such as the arms, legs, and face, leaving permanent scars and severe disabilities. VL or kala-azar is the more serious form and is fatal without treatment. It is characterized by anaemia, enlarged organs (spleen and liver), fever and weight loss. VL is specifically caused by *L. infantum* (*L. inf.*) or *L. donovani* (*L. don.*). *L. inf.* is zoonotic in Latin America, Europe, and Central Asia, where canines act as the major reservoir. In South Asia and East Africa, *L. don.* is considered anthroponotic and is responsible for 95% of VL cases worldwide.^5^ Whilst the numbers of new infections has been decreasing annually and dropped below 20 000 in 2018,^6^ it is estimated that only 20–45% of cases are reported to the WHO.^7,8^ Despite these large numbers, infections by *L. inf.* generally remain sub-clinical and VL only develops in cases where the patient is immunosuppressed, such as patients with HIV or severe malnourishment.^9^ The latter is one of the reasons why leishmaniasis particularly affects poor populations in rural areas.

Today, there are insufficient treatment options (Fig. 1),^5,10^ all of which are associated with severe side-effects and/or other limitations, often requiring intravenous or painful intramuscular injections as well as monitoring in hospital. Miltefosine (MIL) is presently the only approved oral drug for VL and CL. Although MIL has good efficacy, it has a long treatment regimen (28 days); has dose-limiting side-effects (due to vomiting and diarrhoea); is teratogenic which limits use in pregnancy;^11^ and there are reports of resistance and variable efficacy in different geographical areas.^12–14^ Whilst the current pipeline looks more promising than in previous years, with three candidates in phase I and several more in pre-clinical phases,^15–17^ due to the pressing need for new medicines it is imperative that we continue to develop novel, affordable, effective and safe drugs for leishmaniasis.

**Fig. 1:**
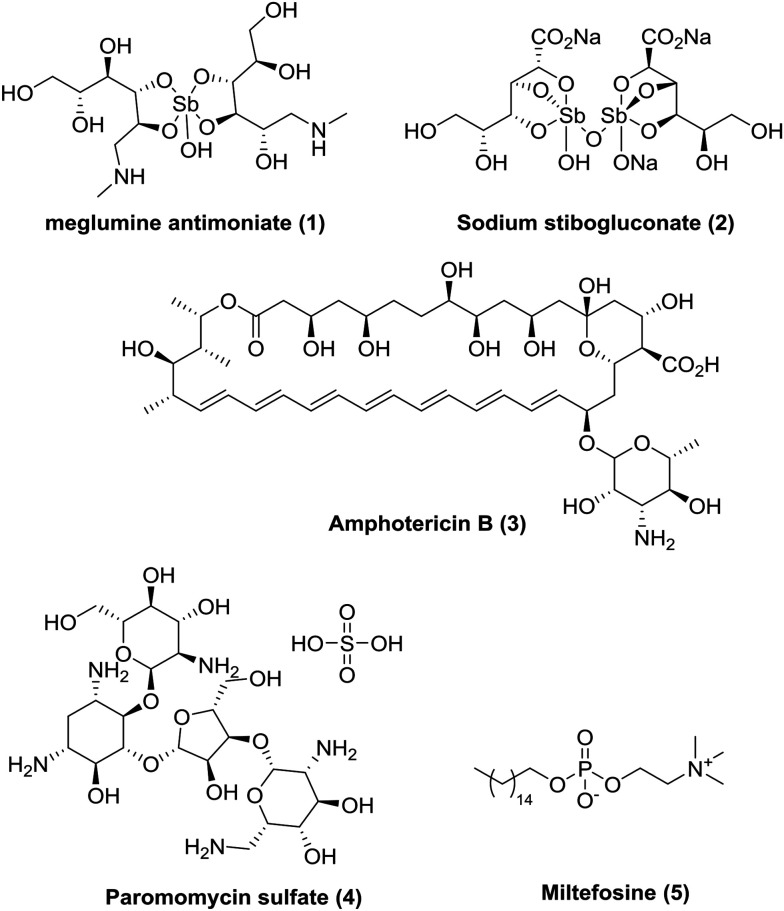
Current available drugs for visceral leishmaniasis.

As part of our collaboration with the Drugs for Neglected Diseases *initiative* (DND*i*), we have been examining new chemical series with anti-trypanosomal activity as potential leads for drug discovery programs.^18^ Sulphonamide hits **6–8** (Fig. 2)^19^ with moderate to good potency (pIC_50_ 4.92–5.82) and selectivity were identified in a cidal high-throughput phenotypic screen of a commercial library against axenic *L. don.* amastigotes (MHOM/SD/62/1S-CL2, LdBOB), as previously described.^20^ Further profiling indicated that intracellular amastigotes of *L. inf.* were slightly more sensitive (pIC_50_ 6.23–6.35). However, all these hits were highly lipophilic (clog *P* ≥ 5) and were rapidly cleared *in vitro* in the presence of both human and mouse microsomes (S9 fractions). To rapidly investigate their structure–activity relationship (SAR), a library of approximately 100 commercial analogues was purchased and tested in parallel against intracellular amastigotes of both *L. inf.* and *L. don.* (see ESI†). Active compounds could be clustered into two sub-sets, both of which contained a 2-alkoxybenzene-sulphonamide moiety (Fig. 2). The first sub-set consisted of a long alkyl chain (at least 4 carbons) on the *ortho*-substituted oxygen and possessed a secondary sulphonamide. The second sub-set consisted of a simple methoxy group at the *ortho* position and a tertiary sulphonamide. Of these sub-sets, no compounds offered a significant advantage over the initial hits in terms of potency or lower lipophilicity. The previous heightened potency against *L. inf.* over *L. don.* (3 to 5-fold) was consistently observed as well, although further investigation of this discrepancy was not pursued in the scope of this work. A hit-to-lead program was initiated focusing primarily on the tertiary sulphonamide sub-set (a) as these compounds offered slightly higher potency and lower lipophilicity, as well as ease of synthesis. It was also felt that improved metabolic stability would be more readily achieved without a large lipophilic alkyl chain in the molecule. The initial hits were divided into two different subsets: tertiary sulphonamides (hit **6**) and secondary sulphonamides (hits **7** and **8**). Attempts to reduce lipophilicity of the hit **6** by replacing the tertiary sulphonamide moiety with a secondary one led to complete loss of potency. The observed loss of potency most likely is related to the drop in log *D* that such modification implies. A mandatory requisite for the potency of secondary sulphonamides (hits **7** and **8**) is the presence of a fatty alkoxy chain on the 2 position of the benzenesulfonyl core. Overall, this requirement tends to increase the log *D* for this class of analogues, leading to compounds with poor metabolic stability.

**Fig. 2:**
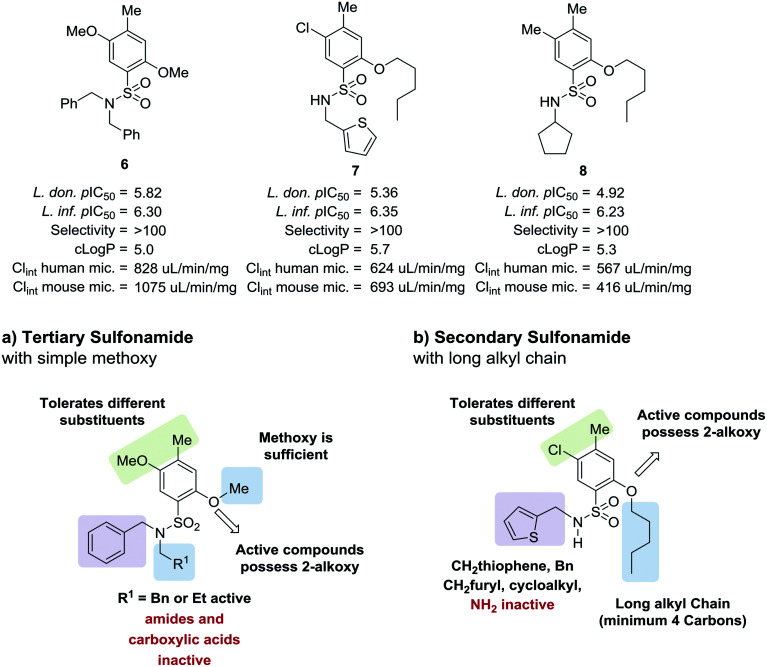
Initial hits and preliminary structure activity relationship (SAR).

Initially, a variety of substituents in the parent aromatic ring was explored in combination with the dibenzylsulphonamide portion (Table 1). It was found that the 4-methyl group from the original hit **6** was not necessary for activity (compound **9**), allowing a reduction in lipophilicity as well as removing a possible metabolic soft spot. As observed in the initial hits **7** and **8**, the 5-methoxy substituent was also not essential for potency (compound **10**), allowing removal of the *p*-dimethoxyaryl moiety, which has been associated with inherent toxicity risks. The 2-alkoxy group, however, was confirmed to be crucial, since removal resulted in a loss of potency (compounds **11–14**). The 2-trifluoromethoxy analogue **15** retained some potency (pIC_50_ = 5.96), but with a large increase in lipophilicity (elog *D* = 5.1) and therefore was not further pursued. Removal of the alkyl ether group to give a phenol was investigated, and 5-cyano analogue **16** was chosen because of ease of synthesis,^21^ but resulted in loss of potency (pIC_50_ = 4.51). Re-installing a methoxy group (compound **17**) restored potency (pIC_50_ = 6.36), confirming that the phenol group is not tolerated.

**Table 1:** Variation of benzene sulphonamide substituents

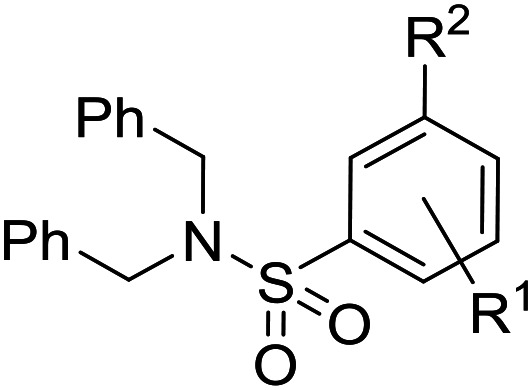								
Compound	R^1^	R^2^	*L. inf.* pIC_50_^a^	PMM pIC_50_^b^	Microsome Cl_int_^c^ (μL min^−1^ mg^−1^)		elog *D*^d^	LLE
					Human	Mouse		
**9**	2-OMe	OMe	6.43 ± 0.24	<4.19	242	1300	4.6	2.86
**10**	2-OMe	H	6.48 ± 0.00	<4.19	n.a.	n.a.	n.a	2.68
**11**	4-OMe	H	4.34 ± 0.15	<4.19	184	2412	4.7	0.70
**12** e	2-F	F	<4.19	<4.19	n.a.	n.a.	n.a.	0.07
**13**	2-CN	H	4.95 ± 0.00	<4.19	699	n.a.	4.3	1.40
**14**	2-SO_2_Me	H	4.49 ± 0.06	<4.19	417	2880	4.0	1.77
**15**	2-OCF_3_	H	5.96 ± 0.21	<4.19	92.9	689	5.1	1.15
**16** e	2-OH	CN	4.51 ± 0.05	4.34 ± 0.15	27.4	1100	3.7	1.10
**17**	2-OMe	CN	6.36 ± 0.04	<4.19	304	n.a.	4.3	2.88
**18**	2-OMe	F	5.86 ± 0.04	<4.19	955	n.a.	4.7	2.11
**19**	2-OMe	Cl	5.86 ± 0.04	<4.19	407	1352	5.0	1.61
**20**	2-OMe	CO_2_H	4.24 ± 0.05	<4.19	<23	94.0	2.5	1.11
**21**	2-OMe	CONH_2_	5.13 ± 0.04	<4.19	108	1670	3.6	2.40
**22**	2-OMe	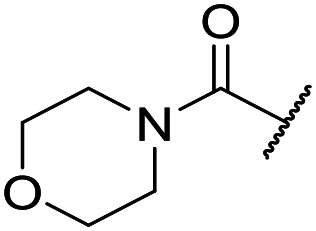	6.34 ± 0.04	4.34 ± 0.15	810	2025	3.8	3.02
**23** e	2-OMe	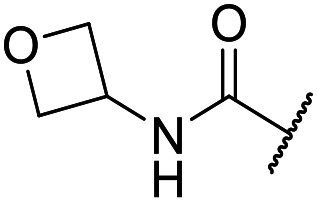	6.10 ± 0.00	<4.19	178	395	4.0	3.11
**24** e	2-OMe	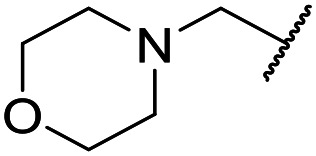	6.28 ± 0.02	<4.19	49.4	3200	4.3	2.84
**25** e	2-OMe	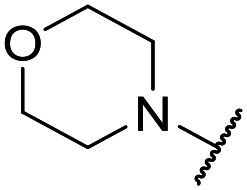	6.49 ± 0.00	<4.19	421	1400	4.2	2.98
**26** e	2-OMe	SO_2_Me	5.89 ± 0.21	<4.19	296	n.a.	3.9	3.04
**27**	2-OMe	SO_2_NH_2_	6.25 ± 0.02	<4.19	94.1	884	3.6	3.84
**28**	2-OMe	SOMe	6.23 ± 0.13	<4.19	233	929	3.5	3.08
**29**	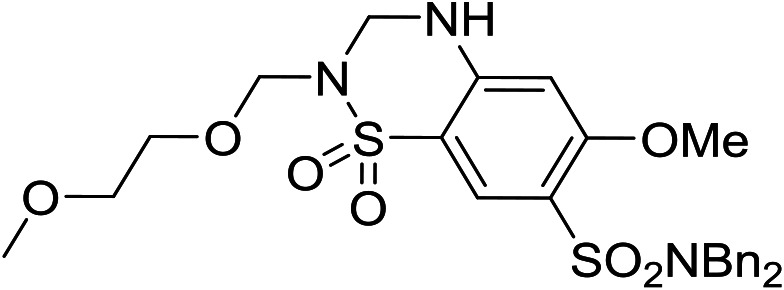		6.18 ± 0.08	<4.19	518	1370	4.0	3.69
**30**	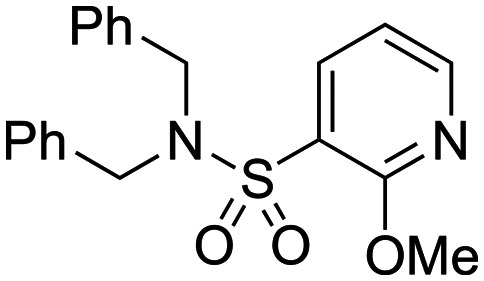		6.19 ± 0.09	4.19	849	2567	4.3	3.20
**31**	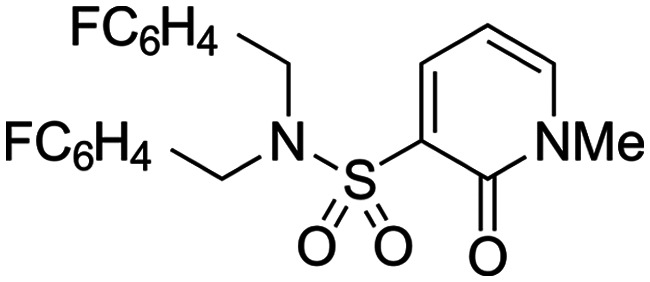		4.68 ± 0.21	4.19	676	2450	3.7	2.42

a Geometric mean of a minimum of two experiments ± standard error. Assay was run according to experimental description (see ESI†) using miltefosine as a positive control.

b Geometric mean of a minimum of two experiments ± standard error. Assay was run according to experimental description (see ESI†) using tamoxifen as a positive control.

c Intrinsic *in vitro* clearance calculated using liver microsomes.

d HPLC measured log *D*.

e Analogues with two *p*-fluorobenzyl groups on sulphonamide nitrogen instead of simple benzyl groups.

The substituent in the 5-position was further explored and found to be tolerant towards almost all changes (see Table 1, compounds **17–28**), except for the carboxylic acid **20** (pIC_50_ = 4.24). Nitrile **17**, amides **22** and **23**, sulfone **26**, sulphonamide **27**, and sulfoxide **28** were the most promising analogues offering the best ligand-lipophilicity efficiency (LLE) and improved *in vitro* clearance (albeit still too high for *in vivo* studies). Morpholine **25** was the most potent of these analogues (pIC_50_ = 6.49) but was not further pursued due to perceived toxicity risks associated with anilines, and the likelihood of oxidation to a reactive iminoquinone-like intermediate.

It was hypothesized that the large degree of tolerability around the 5-position was consistent with these substituents pointing towards solvent. Compound **29** provides more support for this idea, as the capped methoxyethoxymethyl (MEM) derivative still possessed sub-micromolar potency, raising the exciting prospect of using polyethyleneglycol (PEG) based linkers as tool compounds to explore the mechanism of action.

Finally, we investigated some heterocyclic aromatic sulphonamides, such as compound **30**, which retained potency but led to poor metabolic stability. Pyridinone analogues unfortunately showed a significant loss of potency (see Table 1, compound **31** and ESI†).

The lack of improvement in *in vitro* clearance from changes to the upper portion of the molecule suggested that metabolic soft spots are likely to be present in the substituents on the sulphonamide nitrogen. It was hoped that changes here could result in improved metabolic stability. The two suspected spots for metabolic oxidation were the *para*-positions of the benzene ring and the benzyl positions. Firstly, fluorination at the *para*-position was tested and offered similar potency (pIC_50_ = 6.27 for **32***versus* 6.43 for **9**), although clearance data could not be obtained for this analogue (failed in automatic detection high-throughput assay). Replacement of the 4-fluorobenzyl group with a benzene ring (or pyridine – see ESI†) reduced potency by more than 1.5 log units (Table 2, compound **33**, pIC_50_ = 4.94). We next examined smaller or more polar alternatives (see Table 2, compounds **33–42** and ESI†). At best, these analogues led to a 0.75 log unit reduction in potency *versus* the parent dibenzyl analogue **9**, and in some cases even complete loss of activity. The most promising analogue was the methoxyethane derivative **38** which still possessed moderate potency (pIC_50_ = 5.68) as well as lower lipophilicity (elog *D* = 3.5) and slightly improved metabolic stability compared to previous analogues. Replacing the 2,5-dimethoxybenzene group of **38** with other substituents from Table 1, such as the cyano and morpholine amide moieties, further improved the *in vitro* clearance as exemplified by compounds **41** and **42**, but with a loss of potency (pIC_50_ = 5.05 and 4.36 respectively). It is possible that the elog *D* of this series has a lower limit of about 3.5, as no compounds have been observed with good potency (pIC_50_ ≥ 6.0) below this value.

**Table 2:** Variation of substituents on sulphonamide nitrogen

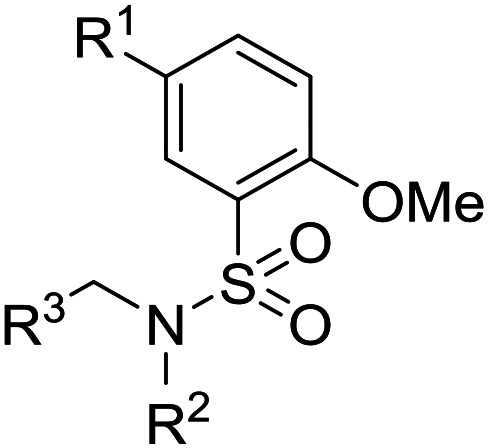									
Entry	R^1^	R^2^	R^3^	*L. inf.* pIC_50_^a^	PMM pIC_50_^b^	Microsome Cl_int,u_^c^ (μL min^−1^ mg^−1^)		elog *D*^d^	LLE
						Human	Mouse		
**32**	OMe	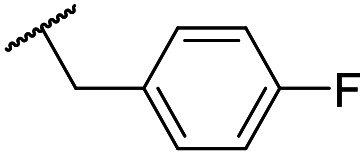	C_6_H_4_F	6.27 ± 0.00	<4.19	n.a.	n.a.	4.7	2.49
**33**	OMe	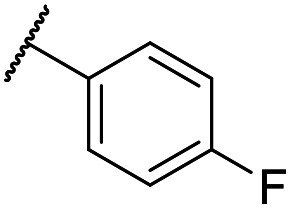	C_6_H_4_F	4.94 ± 0.25	<4.19	482	1560	4.3	1.60
**34**	OMe	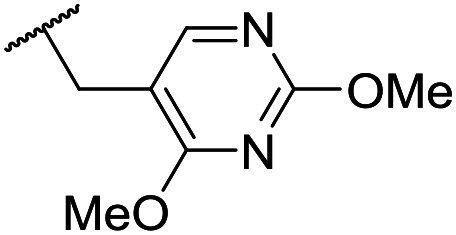	C_6_H_4_F	4.27 ± 0.08	<4.19	498	3148	3.8	1.58
**35**	OMe	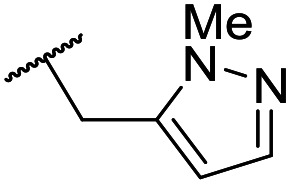	C_6_H_4_F	5.28 ± 0.26	4.34 ± 0.15	651	5480	3.5	3.49
**36**	OMe	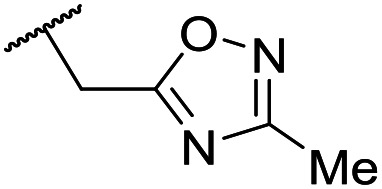	C_6_H_4_F	5.11 ± 0.05	<4.19	350	3054	3.5	3.03
**37**	OMe	Me	C_6_H_4_F	4.93 ± 0.04	<4.19	414	n.a.	3.7	2.67
**38**	OMe	Et	Ph	5.60 ± 0.03	<4.19	534	3497	3.8	3.04
**39**	OMe	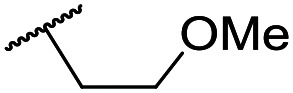	Ph	5.68 ± 0.00	<4.19	253	n.a.	3.5	3.62
**40**	OCF_3_	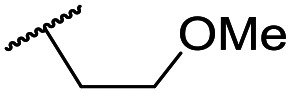	C_6_H_4_F	5.08 ± 0.11	<4.19	194	222	3.8	1.78
**41**	CN	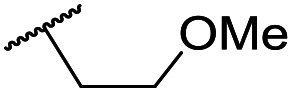	C_6_H_5_F	5.05 ± 0.03	<4.19	<23	585	3.4	2.98
**42**	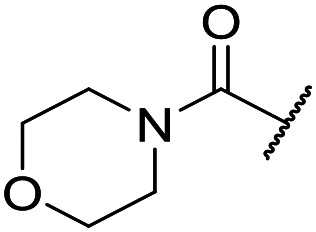	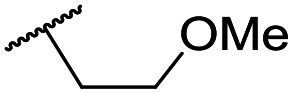	C_6_H_5_F	4.36 ± 0.06	<4.19	28	60	2.9	2.74
**43**	CN	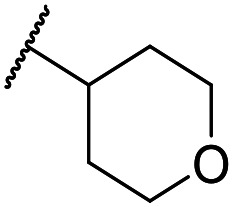	C_6_H_5_F	<4.19	<4.19	194	253	3.4	1.64
**44**	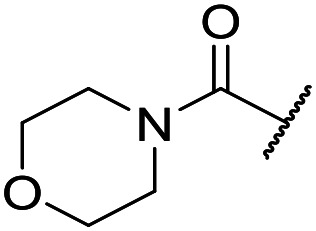	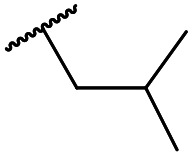	C_6_H_5_F	5.16 ± 0.13	<4.19	1050	1400	3.6	2.08
**45**	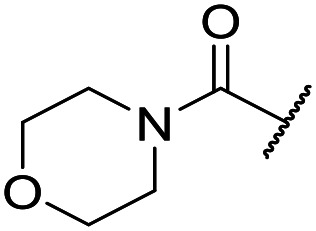	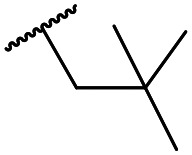	Ph	6.00 ± 0.10	<4.19	1600	2580	3.8	2.45
**46** e	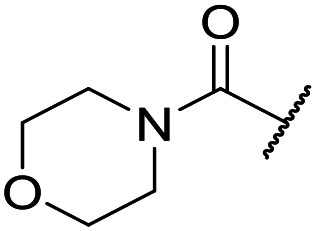	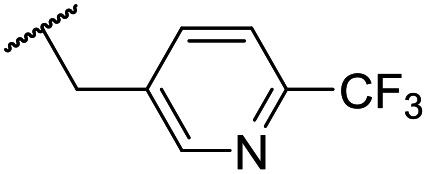	C_6_H_5_F	5.76 ± 0.06	<4.19	265	534	3.7	2.44
**47** e	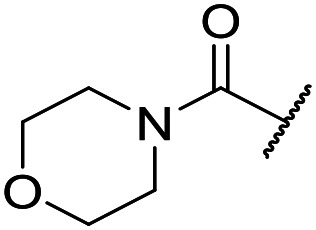	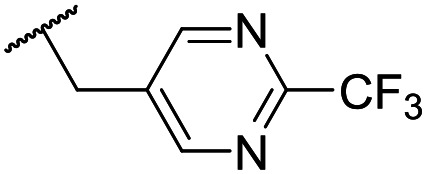	Ph	5.35 ± 0.25	<4.19	73	152	3.5	2.86
**48**	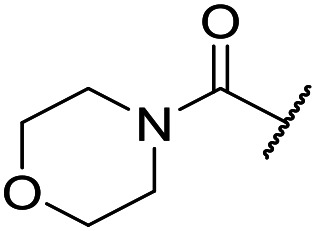	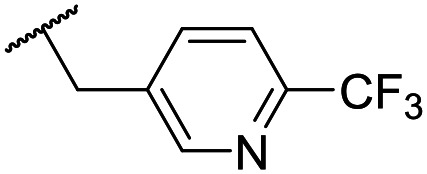	^*t*^Bu	4.34 ± 0.15	<4.19	143	379	3.6	0.89
**49**	CN	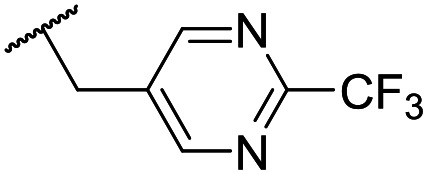	Ph	5.41 ± 0.16	<4.19	100	n.a.	3.8	2.76
**50**	CN	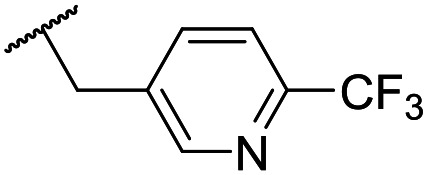	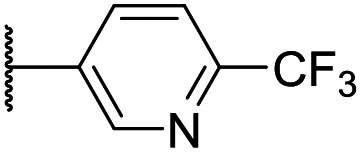	4.41 ± 0.23	<4.19	<23	95	3.8	1.13

a Geometric mean of a minimum of two experiments ± standard error. Assay was run according to experimental description (see ESI†) using miltefosine as a positive control.

b Geometric mean of a minimum of two experiments ± standard error. Assay was run according to experimental description (see ESI†) using tamoxifen as a positive control.

c Intrinsic *in vitro* clearance calculated using liver microsomes.

d HPLC measured log *D*.

e Expected degradation product would be carboxylic acid **20** which was inactive.

At this point, results from metabolite identification (MetID) experiments (see ESI† Tables S2 and S3) suggested that the benzyl CH_2_ was indeed the major metabolic soft spot as previously postulated. Several analogues containing sterically hindered aliphatic groups as lipophilic alternatives to the benzyl group were synthesised to reduce clearance whilst maintaining potency (Table 2, compounds **43–45**). The more polar 4-tetrahydropyranyl **43** was inactive and the isobutyl analogue **44** only possessed moderate potency against *L. inf.* (pIC_50_ = 5.16). The bulkier neopentyl analogue **45** showed good potency (pIC_50_ = 6.00) albeit without much improvement in metabolic stability. The substituents which led to the largest improvement in clearance without completely compromising potency were trifluoro-pyridine and trifluoro-pyrimidine analogues (Table 2, compounds **46–47**). Replacement of a phenyl group with a trifluoro-pyridine, as in **46**, gave a 4-fold improvement in the *in vitro* mouse clearance but suffered from a decrease in potency (pIC_50_ = 5.76). The trifluoro-pyrimidine analogue **47** gave a further 4-fold improvement in clearance but once more with a drop in potency (pIC_50_ = 5.35). Attempts to switch the morpholine amide for a nitrile group (compound **49**) did not lead to any improvement, and the replacement of the second benzyl group with a neopentyl (compound **48**) or another trifluoropyridyl (compound **50**) resulted in inactive compounds, albeit with further improvements in metabolic stability.

Despite not having identified a likely lead, the compounds with the best compromise between potency and clearance, trifluoro-pyridine **46** and trifluoro-pyrimidine **47**, were examined in more detail (Table 3). In addition to the poor *in vitro* clearance it was found that these compounds had extremely low aqueous solubility, which is likely to be due to their high lipophilicity. Furthermore, they possessed surprisingly poor stability with only 22% and 42% (for **46** and **47**, respectively) remaining after 6 h incubation in mouse plasma. Amide hydrolysis may be responsible for poor stability, although this has not been observed in microsomes, but further analogues would need to be tested to confirm this hypothesis.

**Table 3:** Further DMPK assays of compounds **46** and **47**

Compound	**46**	**47**
*L. inf.* pIC_50_	5.76	5.35
*In vitro* Cl_int_ (human)	265 μL min^−1^ mg^−1^	73 μL min^−1^ mg^−1^
*In vitro* Cl_int_ (mouse)	534 μL min^−1^ mg^−1^	152 μL min^−1^ mg^−1^
Plasma protein binding (PPB)	95.9%	88.3%
Mouse plasma stability (6 h)	22% remaining	42% remaining
Kinetic solubility (pH 2.0)	3.8 μg mL^−1^	11.3 μg mL^−1^
Kinetic solubility (pH 7.4)	3.2 μg mL^−1^	6.2 μg mL^−1^

Despite evaluating almost 200 compounds, no suitable candidate with an acceptable balance of potency and physicochemical properties could be identified, and it was decided to halt further work on this series. In particular, the potency of these sulphonamides appears to be dependent on lipophilicity with almost all compounds with elog *D* < 3.5 losing activity. As can be seen in Fig. 3, it proved difficult to synthesise compounds with LLE > 4. It should be noted that attempts to return to the initially identified subset of secondary sulphonamides (Fig. 2) and improve lipophilicity also led to significant reduction in potency (see ESI†).

**Fig. 3:**
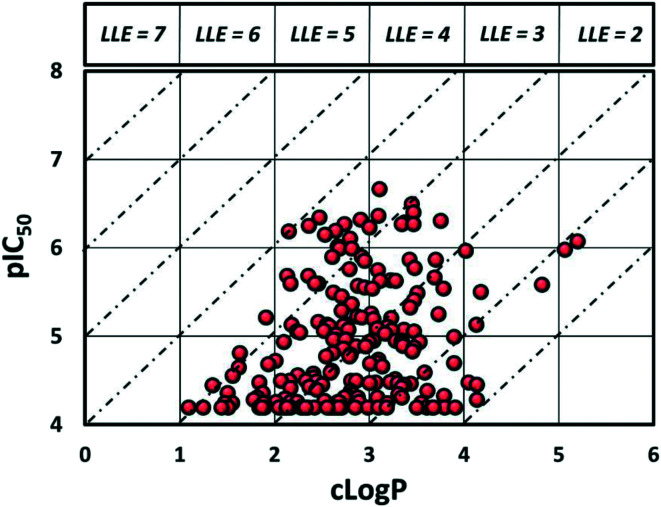
Plot of lipophilicity *versus* potency.

One of the reasons why drug discovery is notoriously challenging in leishmaniasis is the difficulty of reaching the parasites. The amastigotes reside in parasitophorous vacuoles inside the hosts' macrophages,^22,23^ requiring molecules to cross three cell membranes with differing pH gradients.^10^ There are several examples of target-based approaches for leishmaniasis failing to translate into cell-based assays due to this difficulty in permeating the parasite.^24–26^ This may be responsible for the loss of potency in the benzenesulphonamide compounds with lower lipophilicities. Additionally, given the species difference in drug sensitivity observed during initial SAR exploration, we also examined the activity of several analogues against *L. inf.* in a different, immortalized, cell line (THP-1). We noticed significant variability in potency (see ESI† Table S1), suggesting different host cell characteristics and host–parasite interactions may play an important role in the antileishmanial activity of this series and ultimately impact hit discovery campaigns.^27–29^ This may be of relevance when considering host cell selection for future screening efforts against leishmaniasis, particularly given the ethical and logistical constraints of using primary *versus* immortalized cells.

Despite the difficulties in optimizing these compounds, this series showed good potency and selectivity against intracellular forms of *Leishmania* spp. covering several species of CL and VL (see ESI†). The possibility of using these tool compounds in detailed mode of action studies is currently being explored. Such studies may provide an opportunity to use target-based medicinal chemistry to overcome the current issues with this series.

## Material and methods

### Parasite and cell cultures

*Leishmania infantum* MHOM/MA(BE)/67/ITMAP263 and *L. donovani* MHOM/ET/67/L82 amastigotes were collected from the spleen of an infected donor Golden hamster and used to infect primary peritoneal mouse macrophages (PMM). PMM were collected 2 days after peritoneal stimulation of Swiss mice with a 2% potato starch suspension and maintained in RPMI-1640 medium supplemented with 200 mM l-glutamine and 5% FCS. Cultures and assays were maintained at 37 °C under an atmosphere of 5% CO_2_.

### Compound solutions/dilutions

Compound stock solutions were prepared in 100% DMSO at 20 mM. The compounds were serially pre-diluted (2-fold) in 100% DMSO to maintain maximal solubility, followed by a one-step further dilution in demineralized water to achieve a final in-test DMSO concentration of <1%. The compounds were tested at 2-fold compound dilutions covering a range of 64 to 0.00024 μM.

### Susceptibility assay

For the intracellular amastigote susceptibility assay, 3 × 10^4^ PMM were seeded in a 96-well plate. After 24 h, 5 × 10^5^ amastigotes per well were added and incubated for 2 h at 37 °C. The compound dilutions were added next and the plates were further incubated for 5 days at 37 °C and 5% CO_2_. Total parasite burdens were microscopically assessed after Giemsa staining. In treated wells with high amastigote burdens, an overall estimate of the total burden per well was made without discrimination between the number of infected macrophages and the number of amastigotes per infected cell. In treated wells with low burdens, exact counting was performed. The results were expressed as % reduction in parasite burdens compared to control wells and an IC_50_ was calculated. Miltefosine was included as the reference drug (IC_50_ ∼5–10 μM).

### Cytotoxicity assays

The evaluation of toxicity to PMM was part of the *in vitro* susceptibility assay, determined by microscopic evaluation of cell detachment, lysis, and granulation. Evaluation was done by semi-quantitative scoring (no exact counting was performed) of at least 500 cells distributed over adjacent microscopic fields. The results were expressed as % reduction in normal cells compared to untreated control wells and an CC_50_ (50% cytotoxic concentration) was determined. Tamoxifen was used as reference for cytotoxicity (CC_50_ ∼ 5–10 μM).

### ADME assays

For experimental determination of log *D*, test compounds were prepared at a physiologically relevant pH of 7.4 at 200 nM and 2% DMSO in a 50/50 mix of mobile phase A (5% methanol in 10 mM ammonium acetate and adjusted to pH 7.4) and B (100% methanol adjusted to pH 7.4) with an appropriate internal standard at 4 nM, and injected onto an Ascentis Express RP Amide column. Retention times were compared to a standard curve of nine commercial drugs covering a log *D* range of −1.86 to 6.1. The retention times of each of the standards was plotted against the published log *D* values. The resulting equation for this line (*y* = *mx* + *b*) was used to calculate the log *D* values for the test compounds where ‘*x*’ was the retention time in minutes of the test compound and the resultant “*y*” was the experimental log *D* value.

For the experimental stability determination of test compounds in liver microsomes in the presence of NADPH, a clearance rate was determined. Assay conditions were 0.25 mg mL^−1^ liver microsomal protein from the species of interest (mouse and human), 0.5 μM test compound, pH 7.4 and 37 °C. The reagent was purchased commercially, and the work did not involve the use of animals or humans. Samples were taken at 0, 5, 10, 15, 20 and 30 min. The reaction was started after *T*_0_ was taken, with the addition of NADPH at 0.5 μM. The reaction was stopped by addition of 95% acetonitrile/5% methanol containing an internal standard. Time point samples were combined in compound groups of six that had been pre-sorted by mono molecular weight and analyzed by LC/MS/MS. Peak area ratios (analyte peak area/internal standard peak area) were converted to % remaining using the area ratio at time 0 as 100%. Half-life (*t*1/2 = ln(2)/*k*) and intrinsic clearance (Cl_int_ = *k* × 1000/(0.25)) in μL min^−1^ mg^−1^ were calculated from % remaining *versus* incubation time. From this plot, the slope (*k*) was determined. Data was qualified if *t*1/2 > 4*X* the last time point.

## Author contribution

P. J. K. took the lead in writing the manuscript; P. J. K. and M. A. D. carried out design and synthetic chemistry efforts; A. M., L. L. G. F., R. C. C. and S. M.-D. carried out biological experiments; L. M., G. C., A. D. A., J. M. K., C. E. M. and L. C. D. conceived experiments, provided guidance about data interpretation and design of compounds; S. C. contributed to compound design, interpretation of results and to writing the manuscript; J. M. K, C. E. M. and L. C. D. conceived and planned the project.

## Conflicts of interest

The authors declare no competing interests.

## Supplementary Material

MD-011-D0MD00165A-s001

MD-011-D0MD00165A-s002
