# Supplementary material for: Hit-to-lead optimization of a benzene sulfonamide series for potential antileishmanial agents
Source: RSC Med Chem. 2020 Aug 25;11(11):1267–74. doi: 10.1039/d0md00165a (PMC8126888; doi:10.1039/d0md00165a)
Supplement: MD-011-D0MD00165A-s002 [file MD-011-D0MD00165A-s002.pdf]

Electronic Supplementary Material (ESI) for RSC Medicinal Chemistry.
This journal is © The Royal Society of Chemistry 2020

SUPPORTING INFORMATION FILE

Hit-to-Lead Optimization of a Benzene Sulfonamides Series for Potential Antileishmanial Agents

Paul J. Koovits,[a] Marco A. Dessoy,[a] An Matheeussen,[b] Louis Maes,[b] Guy Caljon,[b] Leonardo L. G. Ferreira,[c] Rafael C. Chelucci,[c] Simone Michelan-Duarte,[c] Adriano D. Andricopulo,[c] Simon Campbell,[d] Jadel M. Kratz,[d] Charles E. Mowbray,[d] Luiz C. Dias,[a,*]

[a] Institute of Chemistry, University of Campinas (UNICAMP), Rua Josué de Castro, S/N, Cidade Universitária, Campinas, SP, 13083-861, Brazil

[b] Laboratory of Microbiology, Parasitology and Hygiene (LMPH), Universiteitsplein 1, 2610 Antwerpen, Belgium

[c] Laboratory of Medicinal and Computational Chemistry, Physics Institute of Sao Carlos, University of Sao Paulo, Av. Joao Dagnone 1100, 13563-120, Sao Carlos-SP, Brazil

[d] Drugs for Neglected Diseases *initiative* (DND*i*), 15 Chemin Louis-Dunant, 1202 Geneva, Switzerland

* To whom correspondence should be addressed. L.C.D.: telephone: + 55 19 3521 3097; email: ldias@iqm.unicamp.br

Table of Contents

Biology Experimental Procedures **3**

Parasite and cell cultures *3*

Compound solutions/dilutions *3*

Cytotoxicity assays *4*

Parasitology assays *4*

ADME assays *5*

Secondary parasitology assay using THP-1 as host cell *7*

Secondary cytotoxicity assay using THP-1 .... *8*

Secondary Assay Data **9**

Table S1. Comparison between intracellular anti-leishmanial activity in differing cell lines *9*

Metabolite ID Reports **10**

Table S2. Metabolite ID data for substrate ***23*** *11*

Table S3. Metabolite ID data for substrate ***37*** *13*

Synthetic Chemistry **14**

General Experimental *14*

Compounds from Table 1 .... *15*

Compounds from Table 2 .... *41*

NMR Spectra **63**

Biology Experimental Procedures

Parasite and cell cultures

Trypanosoma cruzi, Tulahuen CL2, beta-galactosidase strain was used. The strain was maintained on MRC-5 (human lung fibroblast) cells in MEM medium, supplemented with 200 mM L-glutamine, 16.5 mM $NaHCO_3$, and 5% inactivated fetal calf serum (FCS).

Leishmania infantum MHOM/MA(BE)/67/ITMAP263 and *Leishmania donovani* MHOM/ET/67/L82 amastigotes were collected from the spleen of an infected donor Golden hamster and used to infect primary peritoneal mouse macrophages (PMM). PMM were collected 2 days after peritoneal stimulation of Swiss mice with a 2% potato starch suspension and maintained in RPMI-1640 medium supplemented with 200 mM L-glutamine and 5% FCS.

T. b. brucei (Squib-427) and *T. b. rhodesiense* (STIB 900) were cultured in Hirumi-9 medium supplemented with 10% FCS.

All cultures and assays were conducted at 37 °C under an atmosphere of 5% CO_2.

Compound solutions/dilutions

Compound stock solutions were prepared in 100% DMSO at 20 mM. The compounds were serially pre-diluted (2-fold or 4-fold) in DMSO followed by a further (intermediate) dilution in demineralized water to assure a final in-test DMSO concentration of <1%. The compounds were tested at 4-fold compound dilutions covering a range of 64 down to 0.00024 µM.

Cytotoxicity assays

Assays were performed in sterile 96-well microtiter plates, each well containing 10 μL of the watery compound dilutions together with 190 μL of MRC-5_{SV2} inoculum ($1.5x10^5$ cells/mL). Cell growth was compared to untreated-control wells (100% cell growth) and medium-control wells (0% cell growth). After 3 days incubation, cell viability was assessed fluorometrically after addition of 50 μL resazurin per well. After 4h at 37°C, fluorescence was measured (λ_{ex} 550 nm, λ_{em} 590 nm). The results were expressed as % reduction in cell growth/viability compared to control wells and an CC_{50} (50% cytotoxic concentration) was determined. Cytotoxic reference compounds included vinblastine or paclitaxel (CC_{50} <0.01μM).

Parasitology assays

For leishmaniasis assays, 3×10^4 PMM were seeded in each well of a 96-well plate. After 24h, 5×10^5 amastigotes/well were added and incubated for 2h at 37°C. After this period, compound dilutions were added, and the plates were further incubated for 5 days at 37°C and 5% CO_2. Total parasite burdens were microscopically assessed after Giemsa staining. In treated wells with high amastigote burdens, an overall estimate of the total burden per well was made without discrimination between the number of infected macrophages and the number of amastigotes per infected cell. In treated wells with low burdens, exact counting was performed. The results were expressed as % reduction in parasite burdens compared to control wells and an IC_{50} was calculated. Miltefosine was included as the reference drug (IC_{50} ~5 μM).

For Chagas disease, assays were performed in sterile 96-well microtiter plates, each well containing 10 μL of the watery compound dilutions together with 190 μL of MRC-5 cell/parasite inoculum ($4x10^3$ cells/well + $4x10^4$ parasites/well). Parasite growth was compared to untreated-infected controls (100% growth) and noninfected controls (0% growth) after 7 days incubation at 37°C and 5% CO_2. Parasite burdens were assessed after adding the substrate CPRG (chlorophenol red β-D-galactopyranoside): 50 μL/well of a stock solution containing 15.2 mg CPRG + 250 μL Nonidet in 100 mL PBS. The change in color was measured spectrophotometrically at 540 nm after 4 hours incubation at 37°C. The results were expressed as % reduction in parasite burdens compared to control wells and an IC_{50} (50% inhibitory concentration) was calculated. Benznidazole was included as the reference drug (IC_{50} ~2-5 μM).

For human African trypanosomiasis, assays were performed in 96-well plates, each well containing 10 μL of the compound dilution and 190 μL of the parasite suspension (1.5×10^4 trypomastigotes/well for *T. b. brucei* and 4×10^3 for *T. b. rhodesiense*). Parasite growth was assessed after 72h at 37°C by adding resazurin. After 6h (*T. b. rhodesiense*) or 24h (*T. b. brucei*), fluorescence was measured (λex 550 nm, λem 590 nm). The results were expressed as % reduction in parasite growth/viability compared to control wells and an IC_{50} was calculated. Suramin was included as the reference drug (IC_{50} ~0.05 μM).

ADME assays

For the experimental determination of LogD, test compounds were prepared at a physiologically relevant pH of 7.4 at 200nM and 2% DMSO in a 50/50 mix of

mobile phase A (5% methanol in 10mM ammonium acetate and adjusted to pH 7.4) and B (100% methanol adjusted to pH 7.4) with an appropriate Internal Standard at 4nM, and injected onto an Ascentis Express RP Amide column, and retention times are compared to a standard curve of 9 commercial drugs covering a LogD range of -1.86 to 6.1. The retention times of each of the 9 standards is plotted against their literature LogD values. The resulting equation for this line, $y = mx + b$, is used to calculate the LogD values for the test compounds. The retention time in minutes of each test compound is substituted for "x" in the equation, and the resultant "y" is the experimental LogD value for that test compound.

For the experimental stability determination of test compounds in liver microsomes in the presence of NADPH a clearance rate is determined. Assay conditions were 0.25 mg/mL liver microsomal protein from the species of interest (mouse and human), 0.5 uM test compound, at pH 7.4 and 37°C. The reagent was purchased commercially, and the work did not involve the use of animals or humans. Samples were taken at 0, 5, 10, 15, 20 and 30 mins in singlet. The reaction was started after the T0 is taken with the addition of NADPH at 0.5uM. Reaction is stopped by addition of 95% acetonitrile/5% methanol containing an internal standard. Time point samples are combined in compound groups of six that have been pre-sorted by mono molecular weight and analyzed by LC/MS/MS. Peak area ratios (analyte peak area/internal standard peak area) are converted to % remaining using the area ratio at time 0 as 100%. Half-life ($t^{1/2} = \ln(2)/k$) and intrinsic clearance ($Cl_{int} = k \times 1000/(0.25)$) in μL/min/mg were calculated from %

remaining versus incubation time. From this plot, the slope (k) was determined. Data was qualified if t½>4X the last time point.

Secondary parasitology assay using THP-1 as host cell

Leishmania infantum (MHOM/MA/67/ITMAP-263) promastigotes were grown in M199 medium (pH 7.4) supplemented with 10% heat-inactivated fetal calf serum (FCS) at 28˚C. Human leukemia THP-1 cells were grown in RPMI-1640 (FCS 10%) at 37˚C and 5% CO_2. Powder samples were diluted to 10 mM in 100% DMSO. In 96-well nontreated plates, compound solutions were diluted 2-fold in 100% DMSO at 6400, 3200, 1600, 800, 400, 200, 100, 50, 25, 12.5 µM (100x the final assay plate concentrations), transferred to another plate at 10% DMSO plus 90% PBS at the concentrations of 640, 320, 160, 80, 40, 20, 10, 5, 2.5, 1.25 µM (10x the final assay plate concentrations) and finally transferred to the assay plate at 1% DMSO in medium at 64, 32, 16, 8, 4, 2, 1, 0.5, 0.25, 0.125 µM (the final assay plate concentrations). All the solutions were visually assessed microscopically, and no turbidity or precipitation were observed. THP-1 cells were seeded at 2 x 10^4/well (RPMI-1640), using 20 ng/mL of phorbol 12-myristate 13-acetate (PMA) for differentiation of the monocytes into macrophages. After 72 hours of incubation (5% CO_2, 37 ˚C), medium was aspirated and late stage promastigotes were seeded (2 × 10^5/well). After 24 hours, medium was withdrawn to remove extracellular parasites, compounds were added in serial dilutions (100 µL/well) and the plates were incubated (5% CO_2, 37 ˚C, 120 hours). Negative control wells (100% parasite growth) and miltefosine as a positive control were included in all plates. After 120 hours, medium was aspirated, and the cells were

fixed in methanol and Giemsa-stained. The number of intracellular amastigotes per macrophage was counted using an inverted microscope. Growth inhibition was expressed as a percentage of the number of amastigotes per macrophage in the negative control wells. IC_{50} values were automatically calculated using dose-response curves fitted with log of inhibitor concentration vs. normalized response between 0 and 100% with variable slope.

Secondary cytotoxicity assay using THP-1

For cytotoxicity tests, THP-1 cells were seeded at 2 x 10^4/well (RPMI-1640, FCS 10%) with 20 ng/mL of PMA. After 72 hours (5% CO_2, 37 ˚C), medium was removed, compounds were added in 2-fold serial dilutions (0.125 – 64 μM) and the plates were incubated (5% CO_2, 37 ˚C, 120 hours). Negative control wells and doxorubicin as a positive control were included in all plates. After 120 hours, 10 μL of Alamar Blue was added to each well and the plates were incubated for 3 hours (5% CO_2, 37 ˚C). The plates were then read with a microplate fluorometer (excitation wavelength of 536 nm and emission wavelength of 588 nm). Growth inhibition was expressed as a percentage of the fluorescence of the negative control wells. IC_{50} values were automatically calculated as described for the intracellular amastigote assay.

Secondary Assay Data

Table S1. Comparison between intracellular anti-leishmanial activity in differing cell lines.

Entry	Structure	Intracellular pIC_{50} activity		pIC_{50} Cytotoxicity	
		L. inf. in PMM	*L. inf.* in THP-1	PMM	THP-1
**Miltefosine**	-	5.06±0.08	5.71±0.06	-	-
**Amphotericin B**	-	-	5.71±0.09	-	-
**23**	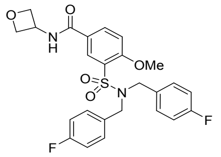	6.10±0.00	5.07±0.04	<4.19	<4.19
**26**	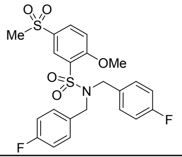	5.89±0.21	5.23±0.01	<4.19	<4.19
**29**	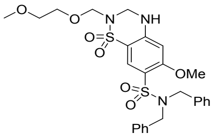	6.18±0.08	4.83±0.06	<4.19	4.40±0.01
**SI51**	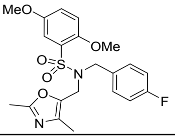	5.63±0.04	4.51±0.03	<4.19	<4.19
**45**	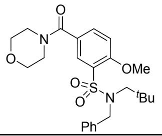	6.00±0.10	4.56±0.05	<4.19	<4.19
**SI52**	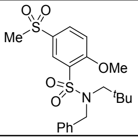	5.56±0.28	4.32±0.01	<4.19	<4.19
**47**	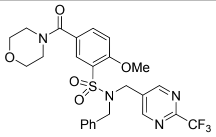	5.35±0.25	<4.19	<4.19	<4.19
**48**	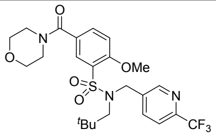	4.34±0.15	<4.19	<4.19	<4.19
**49**	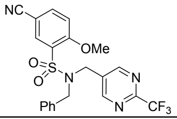	5.41±0.16	4.21±0.02	<4.19	<4.19

Metabolite ID Reports

Metabolite ID report<br>Metabolic soft spot ID	
property	value
Concentration	10μM
Incubation Date	20181009
Incubation Time (h)	1hr
Matrix	S9 Fraction
Species	human, mouse
Project	Neglected Tropical Diseases DNDi_GEN

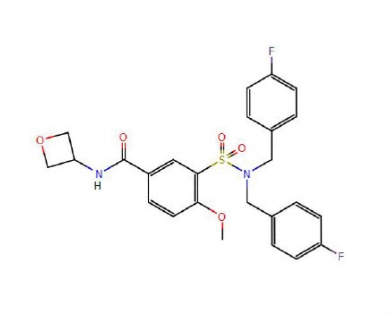

Substrate Name	A-1793948

Notes:

Request purpose: 1) Site of metabolism, 2) Direct conjugation/hydrolysis

Metabolites detected were formed via N-dealkylation and hydroxylation. Amide hydrolysis was not detected.

Species=human

highest value: 2.94E08

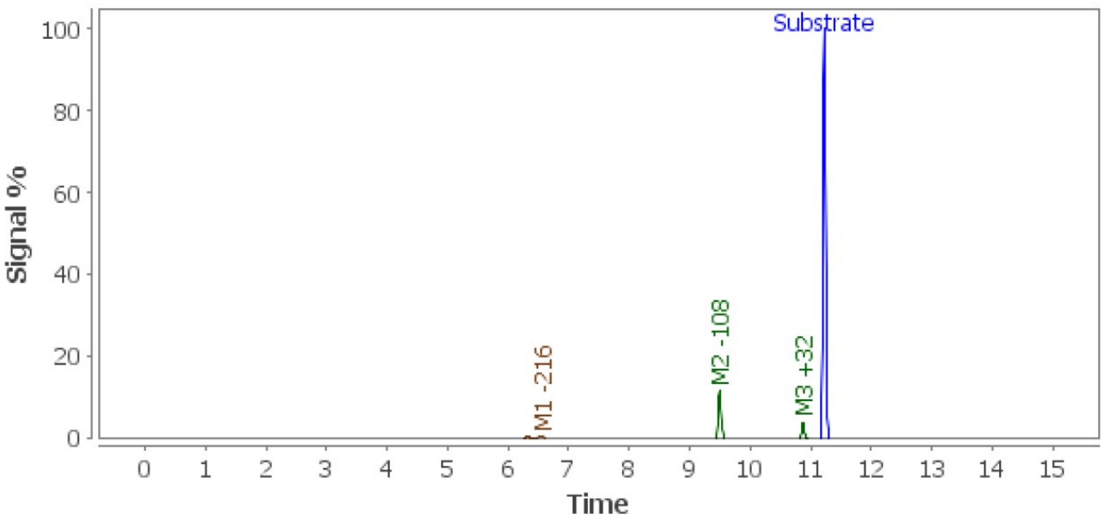

Species=mouse

highest value: 1.72E08

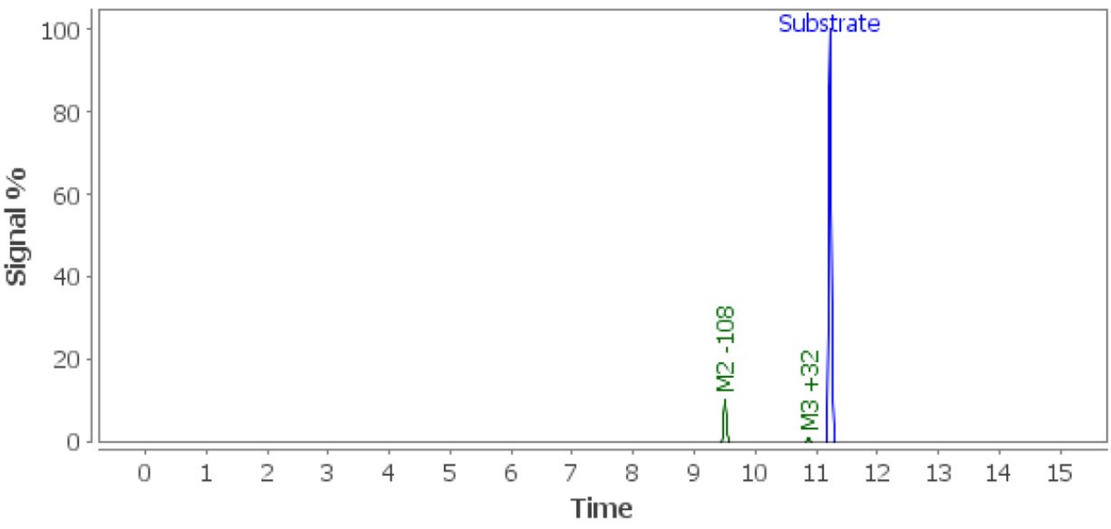

Table S2. Metabolite ID data for substrate **23**

Peak Name	Proposed Structure	Retention Time (min)	m/z	Area%	
				Mouse	Human
Substrate (**23**)	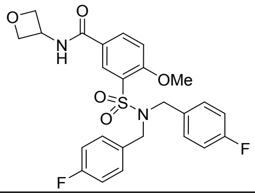	11.15	503.1438	89.1%	84.9%
M1 (-216)	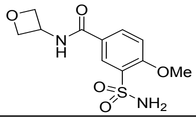	6.56	287.0699	*n.a.*	1.7%
M2 (-108)	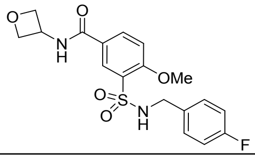	9.50	395.1069	9.9%	10.1%
M3 (+32)	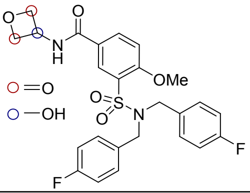	10.88	535.1335	1.0%	3.4%

Metabolite ID report
Metabolic soft spot ID

property	value
Concentration	10μM
Incubation Date	20181009
Incubation Time (h)	1hr
Matrix	S9 Fraction
Species	human, mouse
Project	Neglected Tropical Diseases DNDi_GEN

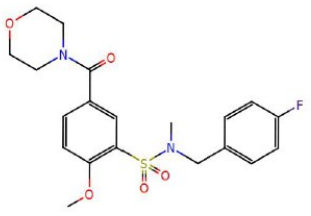

Substrate Name	A-1800675

Notes:

Request purpose: 1) Site of metabolism, 2) Direct conjugation/hydrolysis

Significant turnover to metabolites formed via N-dealkylation (M3, M4) were detected in human. M1 and M2 were formed via N-dealkylation + either N- or O-demethylation. Additional metabolites detected were formed via hydroxylation and amide hydrolysis.

Species=human

highest value: 2.88E08

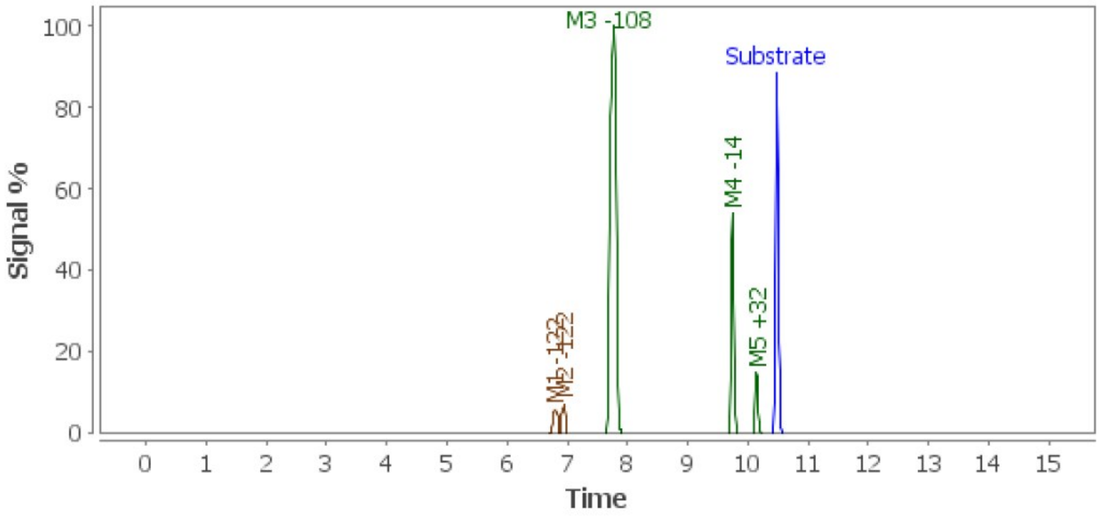

[illegible]

highest value: 1.11E09

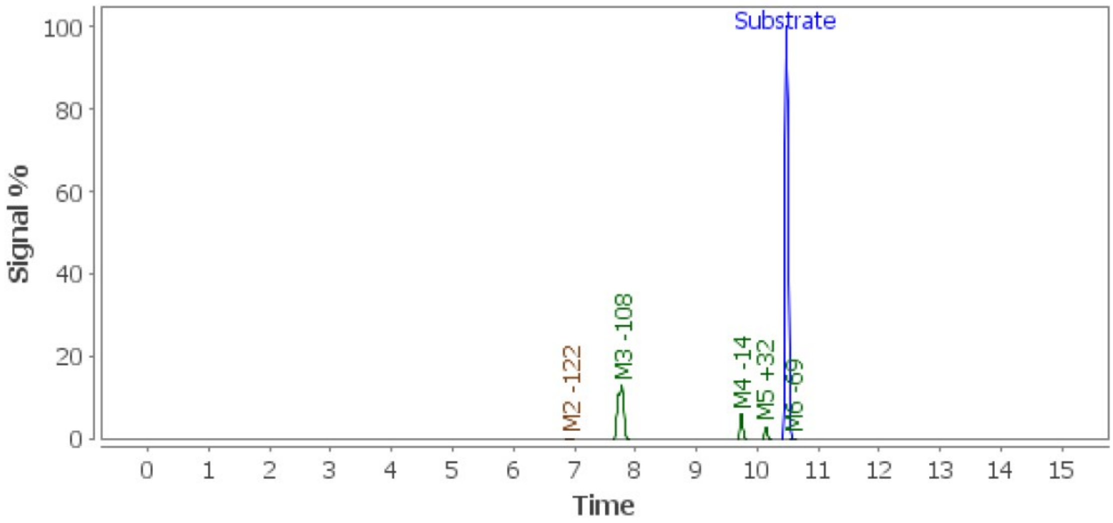

Table S3. Metabolite ID data for substrate **37**

Peak Name	Proposed Structure	Retention Time (min)	m/z	Area%	
				Mouse	Human
Substrate	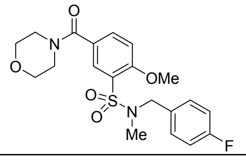	10.50	423.1378	76.1%	22.5%
M1 (-122)	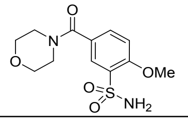	6.79	301.0855	*n.a.*	2.6%
M2 (-122)	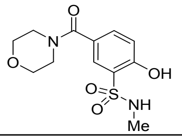	6.95	301.0855	0.1%	2.6%
M3 (-108)	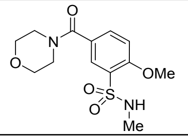	7.78	315.1008	17.7%	54.6%
M4 (-14)	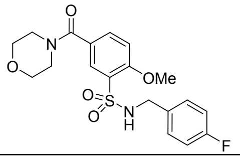	9.74	409.1224	4.1%	14.2%
M5 (+32)	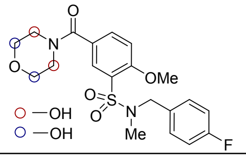	10.14	455.1277	2.0%	3.7%
M6 (-69)	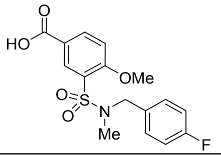	10.60	354.0804	0.1%	*n.a.*

Synthetic Chemistry

General Experimental

Unless specified otherwise for all non-aqueous chemistry, glassware was oven-dried and purged with an inert (Ar) atmosphere. Cryogenic conditions (-78 ˚C) were achieved using solid carbon dioxide/acetone baths. Temperatures of 0 ˚C were obtained by means of an ice bath. Room temperature indicates temperatures in the range of 20-25 ˚C. For the purposes of thin layer chromatography (tlc), Merck silica-aluminium plates were used, with *uv* light (254 nm) and potassium permanganate used for visualisation. For column chromatography, Merck technical grade 60 Å silica gel was used. All NMR data was collected using Bruker Avance III (400 or 500 MHz), or Bruker Avance DPX (250 MHz) instruments. Reference values for residual solvents were taken as δ = 7.26 ($CDCl_3$) and 2.51 ppm (DMSO-*d*6) for ^{1}H NMR; δ = 77.16 ppm ($CDCl_3$) for ^{13}C NMR. Multiplicities for coupled signals were denoted as: s = singlet, d = doublet, t = triplet, q = quartet, m = multiplet, br. = broad, app. = apparent and dd = double doublet *etc.* Coupling constants (*J*) are given in Hz and are uncorrected. Where appropriate, COSY, DEPT, HMBC, HMQC and NOE experiments were carried out to aid assignment. Anhydrous THF was obtained from a sodium-dried solvent still using benzophenone as indicator. Anhydrous dichloromethane, toluene, acetonitrile, triethylamine, diisopropylethylamine, and diisopropylamine were obtained from a CaH_2-dried solvent still.

Compounds from Table 1

General Procedure for Sulfonamide formation

To a solution of sulfonyl chloride (1 mmol, limiting reagent) in CH_2Cl_2 (2 mL per mmol) at 0 ^oC was added diisopropylethylamine (1.5 equiv.), followed by the amine (1.2 equiv.) dropwise. The reaction was stirred until complete, as monitored by tlc (5 min for primary amines, 1-24 h for hindered secondary amines). The reaction was diluted with EtOAc (10 mL), washed with 2M NaOH (5 mL) and 1M HCl (10 mL). The organic was then dried ($MgSO_4$) and concentrated *in vacuo* to give desired product. If final product, the compound was purified by column chromatography to give pure product.

***N,N*-dibenzyl-2,5-dimethoxybenzenesulfonamide (9)**

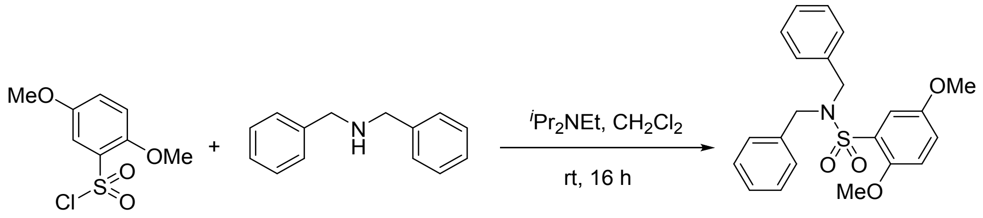

Following the General procedure for Sulfonamide formation 2,5-dimethoxybenzenesulfonyl chloride (160 mg, 0.520 mmol) and N,N-dibenzylamine (124 mg, 0.630 mmol) were reacted for 2 h. After extraction with EtOAc and concentration to dryness in vacuum, the crude product was recrystalized from tert-butyl methyl ether/hexane to give pure N,N-dibenzyl-2,5-dimethoxybenzenesulfonamide 9 (130 mg, 0.303 mmol, 63%) as a colourless crystals; 1H NMR (CDCl3, 400 MHz) δ 7.56 (d, 1H, J=3.2 Hz), 7.1-7.2 (m, 6H), 7.07 (dd, 1H, J=3.2, 9.0 Hz), 7.0 (m, 4H), 6.90 (d, 1H, J=9.0 Hz), 7.0-7.0 (m, 2H),

4.39 (s, 4H), 3.81 (s, 3H), 3.72 (s, 3H); 13C NMR (126 MHz, CDCl3) δ 153.2, 150.8, 136.1, 129.8, 128.5 (2C), 128.3 (2C), 127.5, 120.3, 115.4, 113.6, 56.3, 56.1, 50.3 (2C).

_N,N_-dibenzyl-4-methoxybenzenesulfonamide (11)

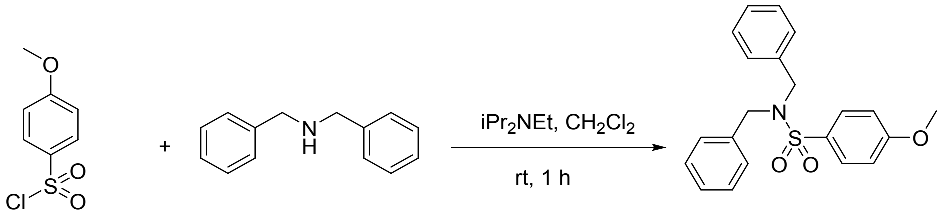

Following the *General procedure for Sulfonamide formation* 4-methoxybenzenesulfonyl chloride (50 mg, 0.240 mmol) and *N,N*-dibenzylamine (57 mg, 0.290 mmol) were reacted for 1 h and purified by column chromatography (10-40% EtOAc in hexane) to give pure N,N-dibenzyl-4-methoxybenzenesulfonamide **11** (80 mg, 0.204 mmol, 85%) □as a colourless solid; ^{1}H NMR ($CDCl_3$, 400 MHz) δ 7.7-7.8 (m, 2H), 7.2-7.3 (m, 6H), 7.0-7.1 (m, 4H), 7.0-7.0 (m, 2H), 4.32 (s, 4H), 3.89 (s, 3H). Data consistent with literature.[1]

2,5-difluoro-_N,N_-bis(4-fluorobenzyl)benzenesulfonamide (12)

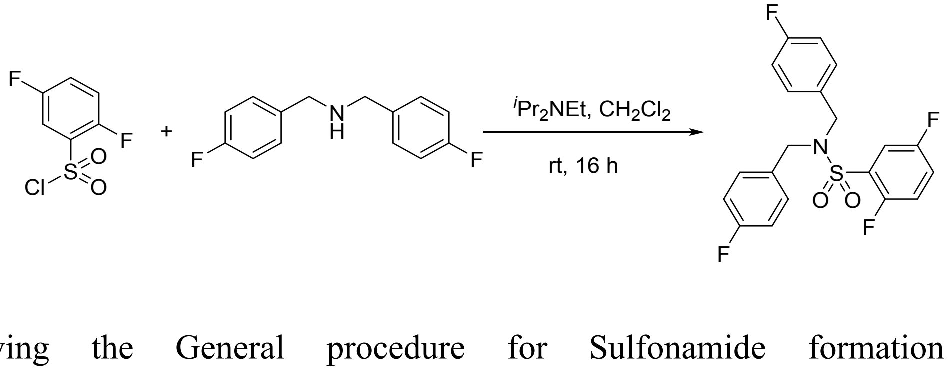

Following the General procedure for Sulfonamide formation 2,5-difluorobenzenesulfonyl chloride (800 mg, 3.760 mmol) and bis(4-

fluorobenzyl)amine (880 mg, 3.800 mmol) were reacted for 1 h and purified by column chromatography (10-40% EtOAc in hexane) to give pure 2,5-difluoro-N,N-bis(4-fluorobenzyl)benzenesulfonamide 12 (800 mg, 1.954 mmol, 52%) as a colourless, crystalline solid; 1H NMR (CDCl3, 500 MHz) δ 7.5-7.6 (m, 2H), 7.2-7.3 (m, 1H), 7.1-7.2 (m, 1H), 7.0-7.0 (m, 4H), 6.8-6.9 (m, 4H), 4.38 (s, 4H); ^{13}C NMR (126 MHz, $CDCl_3$) δ 162.4 (d, *J* = 247.1 Hz), 157.7 (dd, *J* = 248.2, 2.2 Hz), 154,6 (dd, *J* = 251.0, 2.7 Hz), 130.9 (d, *J* = 3.3 Hz), 130.2 (d, *J* = 8.3 Hz), 121.3 (dd, *J* = 24.0, 8.4 Hz), 118.4 (dd, *J* = 24.8, 7.7 Hz), 117.5 (d, *J* = 27.1 Hz), 115.4 (d, *J* = 21.6 Hz), 50.2 (d, *J* = 2.2 Hz).

_N,N_-Dibenzyl-2-cyanobenzenesulfonamide (13)

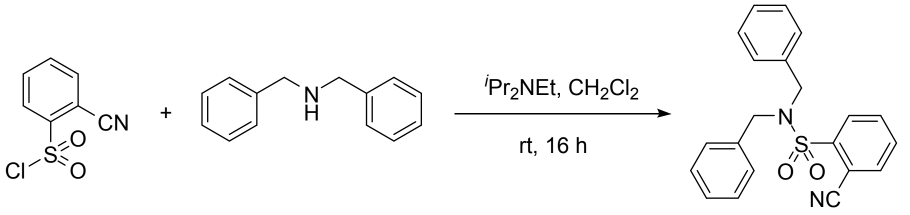

Following the *General procedure for Sulfonamide formation* 2-cyanobenzenesulfonyl chloride (48 mg, 0.240 mmol) and *N,N*-dibenzylamine (57 mg, 0.290 mmol) were reacted for 16 h and purified by column chromatography (10-40% EtOAc in hexane) to give pure *N,N*-dibenzyl-2-cyanobenzenesulfonamide **13** (68 mg, 0.188 mmol, 78%) □as a colourless solid; ^{1}H NMR ($CDCl_3$, 250 MHz) δ 8.0-8.2 (m, 1H), 7.8-8.0 (m, 1H), 7.7-7.7 (m, 2H), 7.2-7.3 (m, 6H), 7.09 (dd, 4H, *J*=2.9, 6.6 Hz), 4.52 (s, 4H); HRMS $C_{21}H_{19}N_2O_2S^+$ calcd. 363.1162, found 363.1151.

N,N-Dibenzyl-2-(methylsulfonyl)benzenesulfonamide (14)

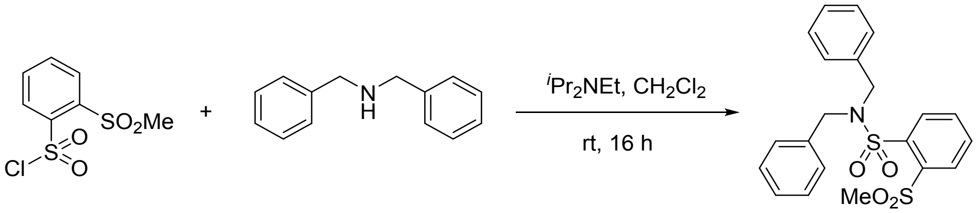

Following the *General procedure for Sulfonamide formation* 2-(methylsulfonyl)benzenesulfonyl chloride (61 mg, 0.240 mmol) and *N,N*-dibenzylamine (57 mg, 0.290 mmol) were reacted for 16 h and purified by column chromatography (10-40% EtOAc in hexane) to give pure *N,N*-dibenzyl-2-(methylsulfonyl)benzenesulfonamide **14** (79 mg, 0.190 mmol, 79%) □as a colourless solid; ^{1}H NMR ($CDCl_3$, 250 MHz) δ 8.42 (dd, 1H, *J*=1.5, 7.9 Hz), 8.18 (dd, 1H, *J*=1.4, 7.8 Hz), 7.75 (dt, 1H, *J*=1.5, 7.6 Hz), 7.65 (dt, 1H, *J*=1.5, 7.6 Hz), 7.2-7.3 (m, 6H), 7.06 (dd, 4H, *J*=2.7, 6.7 Hz), 4.50 (s, 4H), 3.50 (s, 3H); HRMS $C_{21}H_{22}NO_4S_2^+$ calcd. 416.0985, found 416.0997.

N,N-dibenzyl-2-(trifluoromethoxy)benzenesulfonamide (15)

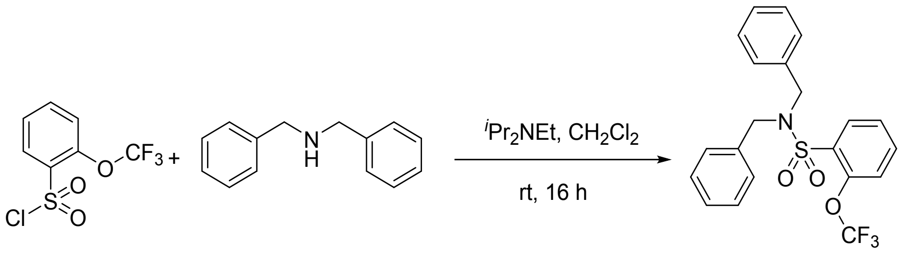

Following the *General procedure for Sulfonamide formation* 2-(trifluoromethoxy)benzenesulfonyl chloride (35 mg, 0.134 mmol) and *N,N*-dibenzylamine (32 mg, 0.160 mmol) were reacted for 1 h and purified by column chromatography (10-40% EtOAc in hexane) to give *N,N*-dibenzyl-5-chloro-2-

methoxybenzenesulfonamide **15** (41 mg, 0.097 mmol, 73%) as a colourless solid. ^{1}H NMR (500 MHz, $CDCl_3$) δ 8.07 (dd, *J* = 7.9, 1.7 Hz, 1H), 7.61 (ddd, *J* = 8.4, 7.6, 1.7 Hz, 1H), 7.36 (dtd, *J* = 11.3, 7.6, 4.2 Hz, 2H), 7.25 – 7.17 (m, 6H), 7.10 – 6.94 (m, 4H), 4.41 (s, 4H); ^{19}F NMR (470 MHz, $CDCl_3$) δ -56.0 (s); ^{13}C NMR (126 MHz, $CDCl_3$) δ 146.1 (*C*), 135.5 (2C, *C*), 134.2 (*C*H), 133.1 (*C*), 131.5 (*C*H), 128.52 (4C, *C*H), 128.50 (4C, *C*H), 127.8 (2C, *C*H), 126.2 (*C*H), 120.3 (q, *J* = 260.2 Hz, *C*), 119.5 (*C*H), 50.36 (2C, *C*H_2); HRMS $C_{21}H_{19}F_3NO_3S^+$ calcd. 422.1032, found 422.1042.

5-Carbamoyl-2-methoxybenzenesulfonyl chloride (SI01)

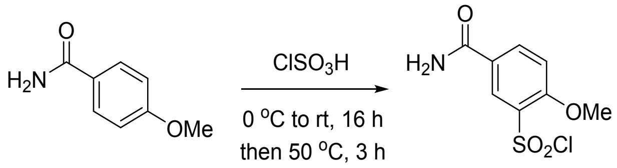

Following the literature procedure,[2] to a pre-cooled flask containing chlorosulfonic acid (6 mL) at 0 °C was added portionwise 4-methoxybenzamide (1.00g, 6.62 mmol). The reaction was stirred at 0 °C for a further 15 min and then allowed to warm to rt and stirred overnight. The reaction was then heated to 50 °C and stirred for 3 h. The reaction was then cooled to 0 °C and then poured onto ice. The resultant precipitate was filtered to give pure 5-carbamoyl-2-methoxybenzenesulfonyl chloride **SI01** (1.32 g, 5.29 mmol, 80%) as an off-white solid; ^{1}H NMR (250 MHz, DMSO-*d6*) δ 8.24 (d, *J* = 2.5 Hz, 1H), 7.85 (dd, *J* = 8.4, 2.5 Hz, 1H), 7.01 (d, *J* = 8.6 Hz, 1H), 3.82 (s, 3H). Data consistent with literature.[2]

5-cyano-2-methoxybenzenesulfonyl chloride (SI02)

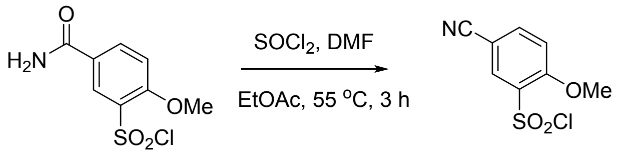

Following the literature procedure,[2] to a solution of 5-carbamoyl-2-methoxybenzenesulfonyl chloride (500 mg, 2.00 mmol) in EtOAc (10 mL) was added DMF (1 drop) followed by thionyl chloride (730 μL, 10.00 mmol) and the reaction was heated to 55 °C and stirred until complete, as monitored by tlc. The reaction was then cooled to rt and concentrated *in vacuo*. The resultant residue was re-dissolved in EtOAc (20 mL) and washed with water (10 mL), sat. brine (10 mL) and dried ($MgSO_4$) to give crude product which was purified by column chromatography (10-20% EtOAc/hexanes) to give pure 5-cyano-2-methoxybenzenesulfonamide **SI02** (381 mg, 1.64 mmol, 82%) as a pale yellow oil. ^{1}H NMR (250 MHz, $CDCl_3$) δ 8.29 (d, *J* = 2.2 Hz, 1H), 7.98 (dd, *J* = 8.8, 2.1 Hz, 1H), 7.25 (d, *J* = 8.7 Hz, 1H), 4.17 (s, 3H). Data consistent with literature.[2]

N,N-dibenzyl-5-cyano-2-methoxybenzenesulfonamide (17)

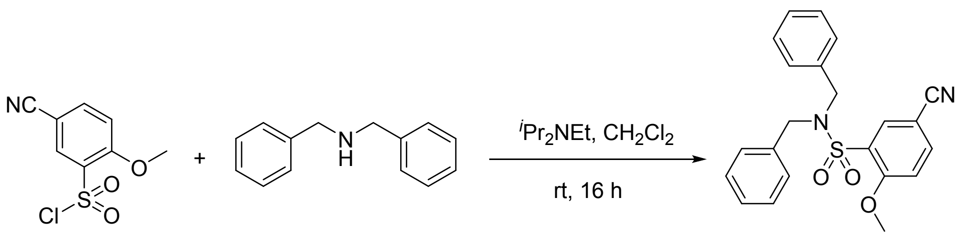

Following the *General procedure for Sulfonamide formation* 5-cyano-2-methoxybenzenesulfonyl chloride **SI02** (35 mg, 0.150 mmol) and *N,N*-dibenzylamine (32 mg, 0.160 mmol) were reacted for 1 h and purified by column

chromatography (10-40% EtOAc in hexane) to give *N,N*-dibenzyl-5-cyano-2-methoxybenzenesulfonamide **17** (48 mg, 0.122 mmol, 82%) as a colourless solid; ^{1}H NMR (400 MHz, $CDCl_3$) δ 8.24 (d, *J* = 2.1 Hz, 1H), 7.78 (td, *J* = 8.9, 2.2 Hz, 1H), 7.33 – 7.21 (m, 6H), 7.11 – 7.05 (m, 4H), 6.99 (dd, *J* = 16.0, 7.7 Hz, 1H), 4.46 (s, 4H), 3.87 (s, 3H); ^{13}C NMR (126 MHz, $CDCl_3$) δ 159.5 (*C*), 138.0 (*C*H), 135.5 (2C, *C*), 135.0 (*C*H), 131.0 (*C*), 128.51 (4C, *C*H), 128.45 (4C, *C*H), 127.9 (2C, *C*H), 117.6 (*C*), 112.8 (*C*H), 104.3 (*C*), 56.5 (CH_3), 50.7 (2C, CH_2); HRMS $C_{22}H_{21}N_2O_3S^+$ calcd. 393.1267, found 393.1245.

5-Cyano-*N,N*-bis(4-fluorobenzyl)-2-hydroxybenzenesulfonamide (16)

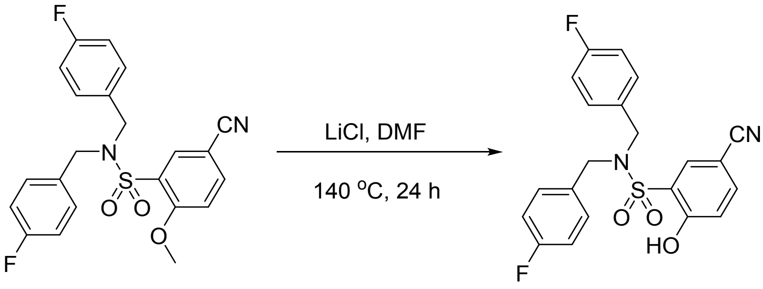

Based on a literature procedure.[2] To a solution of 5-cyano-*N,N*-bis(4-fluorobenzyl)-2-methoxybenzenesulfonamide (100 mg, 0.233 mmol)[a] in DMF (0.5 mL,) was added LiCl (22 mg, 0.500 mmol). The reaction mixture was then heated to 140°C and stirred for 24 h. The reaction was then cooled to rt and the excess solvent removed *in vacuo*. The mixture was then partitioned between EtOAc (10 mL) and water (5 mL). The biphasic mixture was separated and the organic phase was washed with more water (2 x 5 mL), dried ($MgSO_4$) and purified by column chromatography (10-40% EtOAc in hexanes) to give pure *N,N*-

[a] 5-cyano-*N,N*-bis(4-fluorobenzyl)-2-methoxybenzenesulfonamide was made in an analogous procedure to *N,N*-dibenzyl-5-cyano-2-methoxybenzenesulfonamide **17**

dibenzyl-5-cyano-2-hydroxybenzenesulfonamide **16** (56 mg, 0.135 mmol, 58%) as a colourless solid; ^{1}H NMR ($CDCl_3$, 500 MHz) δ 9.44 (br s, 1H), 7.79 (d, 1H, *J*=1.9 Hz), 7.7-7.7 (m, 1H), 7.13 (d, 1H, *J*=8.8 Hz), 7.07 (dd, 4H, *J*=5.3, 8.5 Hz), 6.9-7.0 (m, 4H), 4.35 (s, 4H); HRMS $C_{21}H_{19}N_2O_3S^+$ calcd. 379.1111, found 379.1102.

N,N-dibenzyl-5-fluoro-2-methoxybenzenesulfonamide (18)

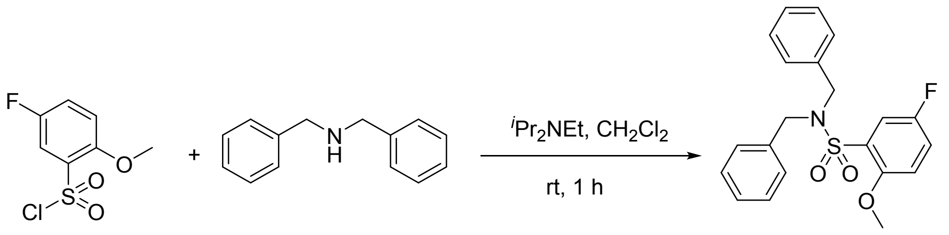

Following the *General procedure for Sulfonamide formation* 5-fluoro-2-methoxybenzenesulfonyl chloride (30 mg, 0.134 mmol) and *N,N*-dibenzylamine (32 mg, 0.160 mmol) were reacted for 1 h and purified by column chromatography (10-40% EtOAc in hexane) to give *N,N*-dibenzyl-5-fluoro-2-methoxybenzenesulfonamide **18** (33 mg, 0.086 mmol, 64%) as a colourless solid; ^{1}H NMR (400 MHz, $CDCl_3$) δ 7.77 (dd, *J* = 8.0, 3.2 Hz, 1H), 7.33 – 7.15 (m, 7H), 7.07 (dt, *J* = 4.6, 3.2 Hz, 4H), 6.92 (dd, *J* = 9.1, 3.9 Hz, 1H), 4.44 (s, 4H), 3.78 (s, 3H); ^{13}C NMR (126 MHz, $CDCl_3$) δ 153.7 (d, *J* = 171.7 Hz, *C*F), 152.9 (*C*O), 135.8 (2C, *C*), 130.6 (d, *J* = 5.9 Hz, *C*), 128.5 (4C, *C*H), 128.4 (4C, *C*H), 127.6 (2C, *C*H), 120.5 (d, *J* = 23.5 Hz, *C*H), 118.0 (d, *J* = 26.4 Hz, *C*H), 113.2 (d, *J* = 7.3 Hz, *C*H), 56.4 (CH_3), 50.4 (2C, CH_2); HRMS $C_{21}H_{21}FNO_3S^+$ calcd. 386.1221, found 386.1236.

N,N-dibenzyl-5-chloro-2-methoxybenzenesulfonamide (19)

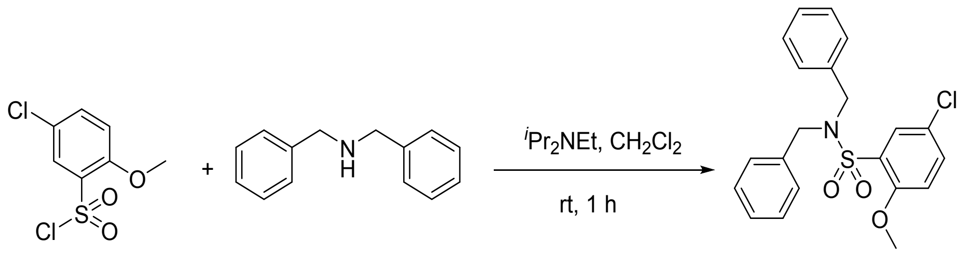

Following the *General procedure for Sulfonamide formation* 5-Chloro-2-methoxybenzenesulfonyl chloride (32 mg, 0.134 mmol) and *N,N*-dibenzylamine (32 mg, 0.160 mmol) were reacted for 1 h and purified by column chromatography (10-40% EtOAc in hexane) to give *N,N*-dibenzyl-5-chloro-2-methoxybenzenesulfonamide **19** (45 mg, 0.112 mmol, 84%) as a colourless solid. ^{1}H NMR (400 MHz, $CDCl_3$) δ 7.97 (d, *J* = 2.7 Hz, 1H), 7.47 (dd, *J* = 8.8, 2.7 Hz, 1H), 7.28 – 7.18 (m, 6H), 7.09 – 7.00 (m, 4H), 6.88 (d, *J* = 8.8 Hz, 1H), 4.41 (s, 4H), 3.76 (s, 3H). ^{13}C NMR (126 MHz, $CDCl_3$) δ 155.2 (*C*O), 135.8 (2C, *C*), 133.9 (*C*H), 130.8 (*C*), 130.7 (*C*H), 128.5 (4C, *C*H), 128.4 (4C, *C*H), 127.7 (2C, *C*H), 125.6 (*C*), 113.4 (*C*H), 56.2 (*C*H_3), 50.4 (*C*H_2); HRMS $C_{21}H_{21}ClNO_3S^+$ calcd. 402.0925, found 402.0919.

3-(*N,N*-dibenzylsulfamoyl)-4-methoxybenzamide (21)

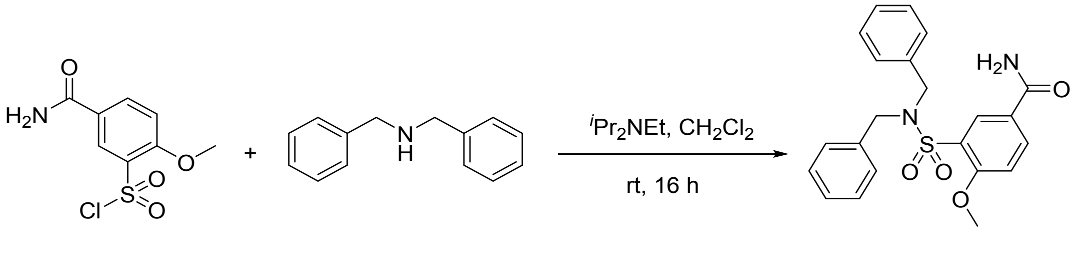

Following the *General procedure for Sulfonamide formation* 5-carbamoyl-2-methoxybenzenesulfonyl chloride (500 mg, 2.00 mmol) and *N,N*-dibenzylamine

(592 mg, 3.00 mmol) were reacted for 1 h and purified by column chromatography (10-80% EtOAc in hexane) to give 3-(*N,N*-dibenzylsulfamoyl)-4-methoxybenzamide **21** (192 mg, 0.468 mmol, 23%) as a colourless solid; ^{1}H NMR (400 MHz, Acetone) δ 8.52 (d, *J* = 2.3 Hz, 1H), 8.22 (dd, *J* = 8.7, 2.3 Hz, 1H), 7.68 (br. s, 1H), 7.32 – 7.20 (m, 7H), 7.11 (dt, *J* = 5.0, 4.0 Hz, 4H), 6.68 (br. s, 1H), 4.46 (s, 4H), 3.95 (s, 3H); HRMS $C_{22}H_{23}N_2O_4S^+$ calcd. 411.1373, found 411.1359.

3-(*N,N*-dibenzylsulfamoyl)-4-methoxybenzoic acid (20)

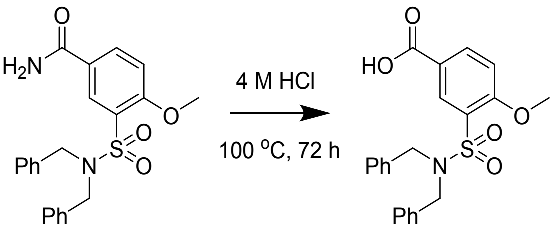

To a suspension of 3-(N,N-dibenzylsulfamoyl)-4-methoxybenzamide (115 mg, 280 μmol) in 1,4-Dioxane (2 mL) was added 4M HCl (600 μL) and the mixture was heated to 90 °C and stirred for 24 h. Reaction allowed to cool to rt and diluted with water (5 mL). Extracted with EtOAc (3 x 10 mL), dried (MgSO4) and concentrated *in vacuo* to give crude product. The crude product was combined with the crude product from 07-PK-604 and purified by column chromatography (1:19:80 to 1:59:40 AcOH:EtOAc:hexanes) to give 3-(N,N-dibenzylsulfamoyl)-4-methoxybenzoic acid **20** (71 mg, 62 %) as a colourless solid; ^{1}H NMR (500 MHz, DMSO) δ 8.36 (d, *J* = 2.2 Hz, 1H), 8.17 (dd, *J* = 8.7, 2.2 Hz, 1H), 7.30 (d, *J* = 8.8 Hz, 1H), 7.27 – 7.16 (m, 6H), 7.10 – 7.02 (m, 4H), 4.38 (s, 4H), 3.90 (s, 3H); ^{13}C NMR (126 MHz, DMSO) δ 166.4 (*C*), 160.0 (*C*), 136.6 (2C, *C*), 136.4 (*C*H), 132.0 (*C*H), 128.8 (*C*), 128.7 (4C, *C*H), 128.5 (4C, *C*H), 127.9 (2C, *C*H), 123.2 (*C*),

113.6 (*C*H), 57.1 (*C*H_3), 51.1 (2C, □*C*□H□2□); HRMS $C_{22}H_{22}NO_5S^+$ calcd. 412.1213, found 412.1233.

N,N-dibenzyl-2-methoxy-5-(morpholine-4-carbonyl)benzenesulfonamide (22)

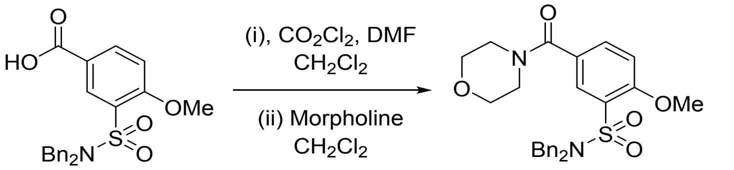

To a solution of 3-(*N,N*-dibenzylsulfamoyl)-4-methoxybenzoic acid (100 mg, 0.243 mmol) in CH_2Cl_2 (2 mL) at 0 °C was added oxalyl chloride (30 µL, 0.365 mmol), followed by DMF (1 drop). The reaction was allowed to warm to rt and stirred for 1 h and then concentrated *in vacuo.* The residue was then re-dissolved in CH_2Cl_2 (2 mL) and morpholine (200 µL) was added. The reaction was stirred for 1 h and then quenched with 1M HCl (5 mL). The mixture was extracted with EtOAc (10 mL), dried ($MgSO_4$) and concentrated *in vacuo* to give crude product which was purified by column chromatography (50-80% EtOAc/hexanes) to give pure *N,N*-dibenzyl-2-methoxy-5-(morpholine-4-carbonyl)benzenesulfonamide **22** (87 mg, 0.180 mmol, 74%) as a colourless solid; ^{1}H NMR ($CDCl_3$, 500 MHz) δ 8.07 (d, 1H, *J* = 2.2 Hz), 7.70 (dd, 1H, *J* = 2.2, 8.6 Hz), 7.2-7.3 (m, 6H), 7.0-7.1 (m, 5H), 4.43 (s, 4H), 3.83 (s, 3H), 3.82 (br s, 8H); HRMS $C_{26}H_{29}N_2O_5S^+$ calcd. 481.1792, found 481.1792.

3-(*N,N*-bis(4-fluorobenzyl)sulfamoyl)-4-methoxy-N-(oxetan-3-yl)benzamide (23)

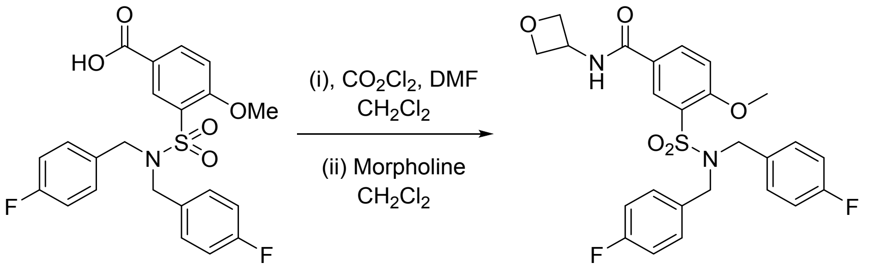

To a solution of 3-(*N,N*-bis(4-fluorobenzyl)sulfamoyl)-4-methoxybenzoic acid (58 mg, 0.130 mmol) in CH_2Cl_2 (2 mL) at 0 ^oC was added oxalyl chloride (18 µL, 0.200 mmol), followed by DMF (1 drop). The reaction was allowed to warm to rt and stirred for 1 h and then concentrated *in vacuo.* The residue was then re-dissolved in CH_2Cl_2 (2 mL) and oxetan-3-amine (27 µL, 0.384 mmol) was added. The reaction was stirred for 1 h and then quenched with 1M HCl (5 mL). The mixture was extracted with EtOAc (10 mL), dried ($MgSO_4$) and concentrated *in vacuo* to give crude product which was purified by column chromatography (50-80% EtOAc/hexanes) to give pure 3-(*N,N*-bis(4-fluorobenzyl)sulfamoyl)-4-methoxy-N-(oxetan-3-yl)benzamide **23** (47 mg, 0.094 mmol, 72%) as a colourless solid; ^1H NMR (400 MHz, $CDCl_3$) δ = 8.32 (d, *J* = 2.4 Hz, 1H), 8.20 - 8.12 (m, 1H), 7.08 (d, *J* = 8.3 Hz, 1H), 7.04 - 6.97 (m, 4H), 6.96 - 6.87 (m, 4H), 6.74 (br d, *J* = 5.9 Hz, 1H), 5.25 (sxt, *J* = 6.8 Hz, 1H), 5.03 (t, *J* = 7.1 Hz, 2H), 4.67 (t, *J* = 6.6 Hz, 2H), 4.38 (s, 4H), 3.90 (s, 3H); HRMS $C_{25}H_{25}F_2N_2O_5S^+$ calcd. 503.1447, found 503.1449.

N,N-bis(4-fluorobenzyl)-2-methoxy-5-(morpholinomethyl)benzenesulfonamide (24)

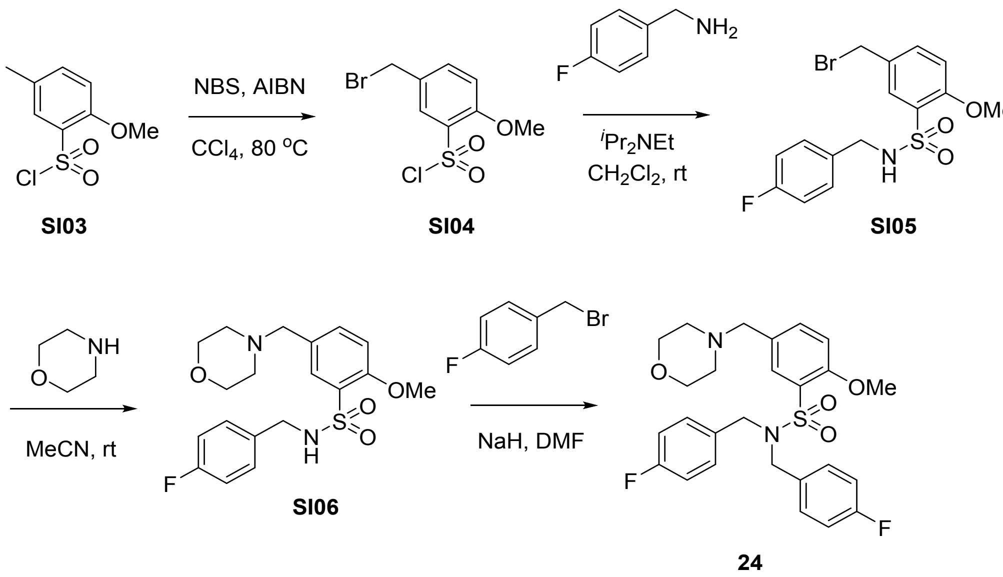

Based on a literature procedure, to a solution of 2-methoxy-5-methylbenzenesulfonyl chloride (1.00 g, 4.53 mmol) in CCl_4 (15 mL) was added AIBN (150 mg, 0.91 mmol) and *N*-bromosuccinimide (887 mg, 4.98 mmol). The mixture was bubbled with N_2 for 15 min and then heated to reflux and stirred for 16 h under an N_2 atmosphere. The reaction was then concentrated *in vacuo* and purified by column chromatography (1-20% EtOAc in hexanes) to give a 4:1 mixture of desired bromo **SI04** and bis-bromo products (471 mg). The mixture was dissolved in DCM (3 mL) and cooled to 0 °C. Triethylamine (280 μL, 2.03 mmol) was added followed by a dropwise addition of (4-fluorophenyl)methanamine (150 μL, 1.35 mmol). The reaction was stirred for The reaction was stirred at rt for 1 h and then partitioned with sat. NH_4Cl (5 mL) and separated. The organic phase was dried ($MgSO_4$) and concentrated in vacuo to give crude sulfonamide mixture which was purified by column chromatography (10-60% EtOAc in hexanes) to

give pure mono-bromo sulfonamide **SI05** (402 mg, 1.04 mmol, 23% over two steps).

Sulfonamide **SI05** (136 mg, 0.350 mmol) was dissolved in MeCN (2 mL) and morpholine (61 μL, 0.701 mmol) was added. The reaction was stirred for 2 h and then concentrated *in vacuo*. The residue was partitioned between EtOAc (5 mL) and water (5 mL), separated and the organic phase was dried ($MgSO_4$) and concentrated in vacuo to give crude morpholine **SI06**.

To a solution of morpholine **SI06** (80 mg, 0.20 mmol) in DMF (1 mL) at 0 °C was added NaH (60 wt%, 12 mg, 0.30 mmol) and TBAI (7.5 mg, 0.02 mmol). The mixture was stirred at 0 °C for 1 h and then 4-fluorobenzyl bromide (28 μL, 0.22 mmol) was added. The reaction was allowed to warm to rt and stirred for 2 h. Water (5 mL) was added and the reaction was extracted with EtOAc (2 x 5 mL). The combined organics were dried ($MgSO_4$) and concentrated *in vacuo* to give crude product which was purified by column chromatography (10-80% EtOAc in hexanes) to give pure *N,N*-bis(4-fluorobenzyl)-2-methoxy-5-(morpholinomethyl)-benzenesulfonamide **24** (72 mg, 0.143 mmol, 71 %) as a colourless solid; ^{1}H NMR (400 MHz, $CDCl_3$) δ = 7.94 (d, J = 2.1 Hz, 1H), 7.54 (dd, J = 1.8, 8.4 Hz, 1H), 7.05 - 6.94 (m, 5H), 6.93 - 6.85 (m, 4H), 4.36 (s, 4H), 3.84 (s, 3H), 3.75 - 3.67 (m, 4H), 3.48 (s, 2H), 2.51 - 2.34 (m, 4H); HRMS $C_{26}H_{28}F_2N_2O_4S^+$ calcd. 503.1811, found 503.1819.

_N,N_-bis(4-fluorobenzyl)-2-methoxy-5-morpholinobenzenesulfonamide (25)

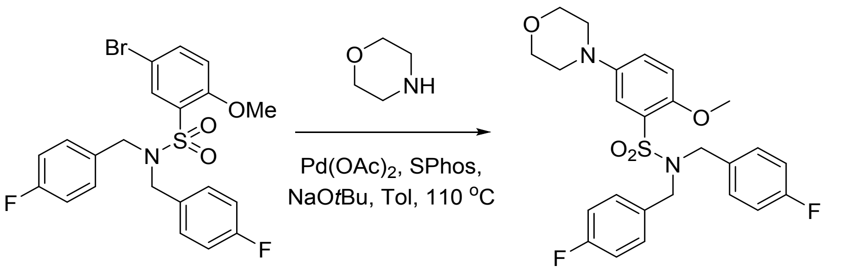

To an oven-dried MW fitted with a stir bar under nitrogen was added $Pd(OAc)_2$ (2.0 mg, 0.008 mmol), SPhos (7.0 mg, 0.017 mmol), NaO^tBu and 5-bromo-N,N-bis(4-fluorobenzyl)-2-methoxybenzenesulfonamide (80 mg, 0.166 mmol). The flask was evacuated and back-filled with N_2 three times and then toluene (1 mL) and morpholine (29 μL, 0.330 mmol) was added. The reaction mixture was bubbled with N_2 for 15 min and then heated to 110 °C and for 4 h. The reaction was then cooled to rt, diluted with EtOAc (5 mL), washed with sat. NH_4Cl (4 mL), dried ($MgSO_4$) and concentrated *in vacuo* to give crude product. The crude product was purified by column chromatography (10-60% EtOAc in hexanes) to give pure *N,N*-bis(4-fluorobenzyl)-2-methoxy-5-morpholinobenzenesulfonamide **25** (32 mg, 0.065 mmol, 39% yield) as an off-white solid; ^1H NMR (400 MHz, $CDCl_3$) δ = 7.57 (d, *J* = 3.1 Hz, 1H), 7.10 (dd, *J* = 3.0, 9.0 Hz, 1H), 7.05 - 6.97 (m, 4H), 6.95 (d, *J* = 9.0 Hz, 1H), 6.93 - 6.86 (m, 4H), 4.35 (s, 4H), 3.92 - 3.84 (m, 4H), 3.80 (s, 3H), 3.12 (dd, *J* = 3.9, 5.6 Hz, 4H); HRMS $C_{25}H_{27}F_2N_2O_4S^+$ calcd. 489.1654, found 489.1648.

N^1,N^1-dibenzyl-6-methoxybenzene-1,3-disulfonamide (27)

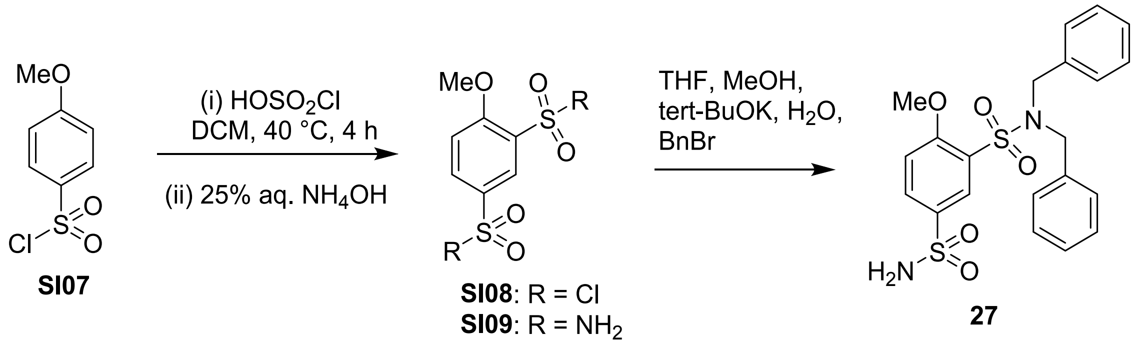

To a solution of **SI07** (2.0 g, 10.0 mmol) in dry dichloromethane (5 mL), neat chlorosulfonic acid (4.7 g, 40.0 mmol) was carefully added (warning: strong HCl evolution!) at room temperature. The mixture was refluxed for 4 hours. The cold reaction mixture was then hydrolysed portion wise on crushed ice (warning: strongly exothermic reaction!). Dichloromethane (25 mL) was added to the ice-cold hydrosylate. The mixture was transferred to a separatory funnel and, after phase separation, the organic layer was washed with water (3 x 10 mL). The wet organic layer was transferred to a round-bottomed flask and cooled down to 0 °C. Concentrated (ca. 25%) aqueous NH_4OH solution was carefully added under vigorous stirring until **SI08** could no longer be detect by means of TLC ($CHCl_3$/MeOH 20:3). The reaction mixture was concentrated and dried in high vacuum to give **SI09** as a greyish solid (1.3 g, 4,88 mmol, 49%).

SI09 (500 mg, 1.88 mmol) was dissolved in a blend of tetrahydrofuran (10 mL) and methanol (10 mL). Solid potassium tert-butoxide (112 mg, 1.0 mmol) was added to give a clean solution. After a few minutes, a white precipitated separated which dissolved upon the addition of water[a)] (10 mL). Neat benzyl bromide (160 mg, 0.95 mmol) was added and the mixture was stirred at room temperature for 5

hours. Extra portions of solid potassium tert-butoxide (112 mg, 1.0 mmol) and benzyl bromide (160 mg, 0.95 mmol) were added and stirring was continued at room temperature for another 5 hours. The reaction progress was monitored by means of silica covered TLC plates ($CHCl_3$/MeOH 20:3). At this point, some unreacted starting material was detected together with 3 major products on the TLC plate. Equimolar amounts of potassium tert-butoxide and benzyl bromide were added until the starting material was completely consumed (4 major products show on the TLC plate). The reaction mixture was concentrated in vacuum and the crude product was extracted with ethyl acetate. The product mixture was separated by column chromatography ($CHCl_3$/MeOH 30:1) to give the pure title product **27** [b)] as a white solid (105 mg, 0.23 mmol, 13%); ^{1}H NMR ($CDCl_3$, 250 MHz) δ 8.48 (d, 1H, J = 2.4 Hz), 8.06 (dd, 1H, J = 2.4, 8.6 Hz), 7.1-7.2 (m, 6H), 7.0-7.2 (m, 4H), 7.0 (d, 1H, J = 8.6 Hz), 4.98 (s, 2H, *NH₂*), 4.42 (s, 4H) 3.83 (s, 3H); ^{13}C NMR (126 MHz, $CDCl_3$) δ 159.3, 135.5, 134.2, 132.7, 130.1, 129.7, 128.45 (2C), 128.43 (2C), 127.3, 112.4, 56.5, 50.6.

[a)] The reaction did not take place in the absence of water. Powdered potassium tert-butoxide was used as a base because it could be handled more conveniently than KOH pellets.

[b)] **27** corresponds to the second spot on the TLC plate (from bottom to top) and the correct regiochemistry was assigned by means of a NOESY experiment.

N,*N*-dibenzyl-2-methoxy-5-(methylsulfinyl)benzenesulfonamide (28)

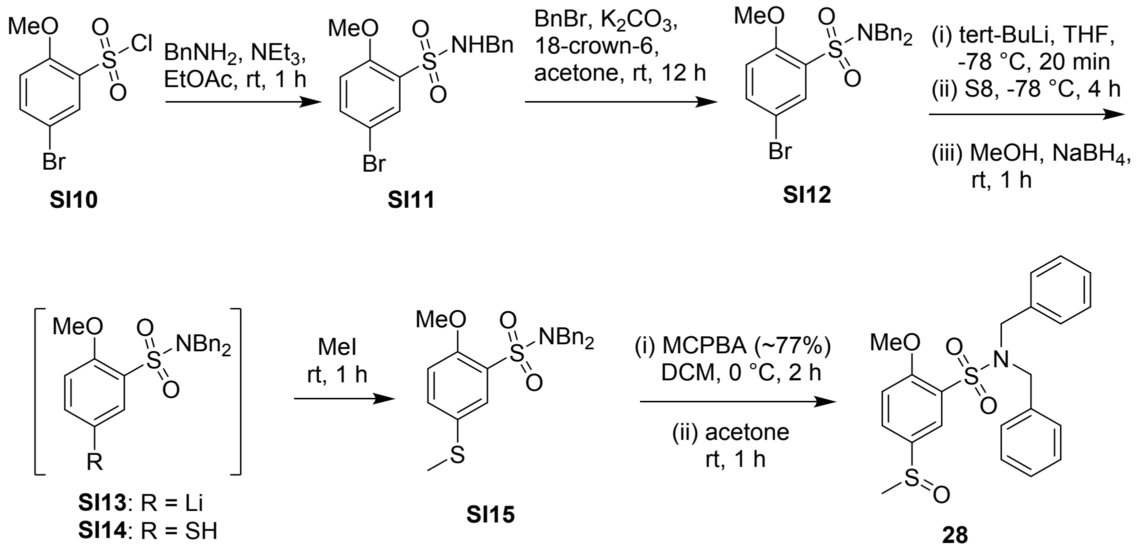

To a solution of **SI10** (1.0 g, 3.50 mmol) in ordinary ethyl acetate (20 mL) was added a mixture of benzylamine (430 mg, 4.0 mmol) and triethylamine (404 mg, 4.0 mmol) at 0 °C. The mixture was stirred for 1 hour and then diluted with ethyl acetate (30 mL). The reaction mixture was washed with water (1 x 10 mL), 1N HCl solution (2 x 10 mL), saturated $NaHCO_3$ solution (1 x 10 mL), and brine. The organic phase was dried over $MgSO_4$, filtered, and concentrated to dryness in vacuum. The solid evaporation residue was resuspended in diethyl ether (5 mL), filtered, and dried in vacuum to give pure **SI11** (1,20 g, 3,37 mmol, 96%) as a white, amorphous solid.

To a solution of **SI11** (550 mg, 1,540 mmol) in ordinary acetone (20 mL) was added solid potassium carbonate (1.51 g, 11.0 mmol), 18-crown-6 ether (10 mg), and benzyl bromide (290 mg, 1.70 mmol). The slurry was vigorously stirred at room temperature for 12 hours. Acetone was evaporated in vacuum and the evaporation residue was partitioned between ethyl acetate (40 mL) and water (15

mL). After phase separation, the organic layer was washed with water (3 x 10 mL) and brine. The organic phase was dried over magnesium sulphate, filtered, and concentrated to dryness in vacuum to give pure **SI12** (690 mg, 1.54 mmol, 100%) as an off-white, crystalline solid.

One-pot procedure: Dry tetrahydrofurane (15 mL) was cooled down to -78 °C to receive the addition of a 1.7 M hexane solution of tert-butyllithium (2.35 mL, ca. 4.0 mmol) under an argon atmosphere. A solution of **SI12** (690 mg, 1.54 mmol) in dry tetrahydrofurane (10 mL) was added within 10 minutes, whereupon a. reddish solution resulted (**SI13**). This solution was stirred at -78 °C for 20 minutes before finely divided sulphur powder (80.0 mg, 2.5 mmol) was added. The slurry was stirred at the same temperature for 4 hours. The reaction was quenched upon careful addition of methanol (20 mL). The reaction mixture was warmed up to 0 °C (ice bath) to receive the addition of solid sodium borohydride (378 mg, 10 mmol). The slurry was stirred at room temperature for 1 hour. **SI14** thus obtained was reacted with excess methyl iodide (709 mg, 5.0 mmol) *in situ* (2 hours, room temperature). The reaction mixture was concentrated in vacuum. The evaporation residue was partitioned between ethyl acetate (30 mL) and water (10 mL). The organic layer was washed with water (10 mL), brine, dried over magnesium sulphate, filtered, and concentrated in vacuum. The crude product was purified by column chromatography (EtOAc/hexanes 1:4) to give pure **SI15** (170 mg, 0.410 mmol, 27%) as a colourless oil.

SI15 (80.0 mg, 0.193 mmol) was dissolved in dichloromethane (5 mL) and the solution was cooled down to 0 °C. A slight excess of technical grade (~77%) 3-

chloro perbenzoic acid (45 mg, ~0.20 mmol) was dissolved in dichloromethane (5 mL). Four milliliters of the oxidant solution were transferred to the reaction vessel within 10 minutes and the mixture was stirred for ca. 2 hours at 0 °C, whilst the reaction progress was monitored by means of TLC ($CHCl_3$/methanol 20:1). Small portions of the remaining oxidant solution were then added until TCL monitoring accused the complete consumption of **SI15** (mitigate the risk of over oxidation!). Acetone (2 mL) was added and the mixture was stirred at room temperature for 1 hour. The reaction mixture was concentrated in vacuum and the evaporation residue was dissolved in ethyl acetate. The organic solution was washed with saturated $NaHCO_3$ solution (3 x 10 mL) and brine; dried over magnesium sulphate, filtered, and concentrated in vacuum. The crude product was purified by column chromatography ($CHCl_3$/methanol 40:1) to give the pure title compound **28** (75 mg, 0.174 mmol, 90%) as colourless oil (solidified on standing at room temperature); ^{1}H NMR ($CDCl_3$, 500 MHz) δ 8.10 (d, 1H, *J* = 2.3 Hz), 7.94 (dd, 1H, *J* = 2.3, 8.6 Hz), 7.1-7.2 (m, 6H), 7.10 (d, 1H, *J* = 8.6 Hz), 7.0 (m, 4H), 4.47 (d, 2H, *J* = 15.5 Hz), 4.37 (d, 2H, *J* = 15.5 Hz), 3.82 (s, 3H), 2.71 (s, 3H); ^{13}C NMR (126 MHz, $CDCl_3$) δ 158.6, 137.2, 135.6, 130.7, 129.6, 128.4 (3C), 127.7, 126.7, 113.2, 56.3, 50.5, 44.1.

N,N-dibenzyl-6-methoxy-2-((2-methoxyethoxy)methyl)-3,4-dihydro-2*H*-benzo[*e*][1,2,4]thiadiazine-7-sulfonamide 1,1-dioxide (29)

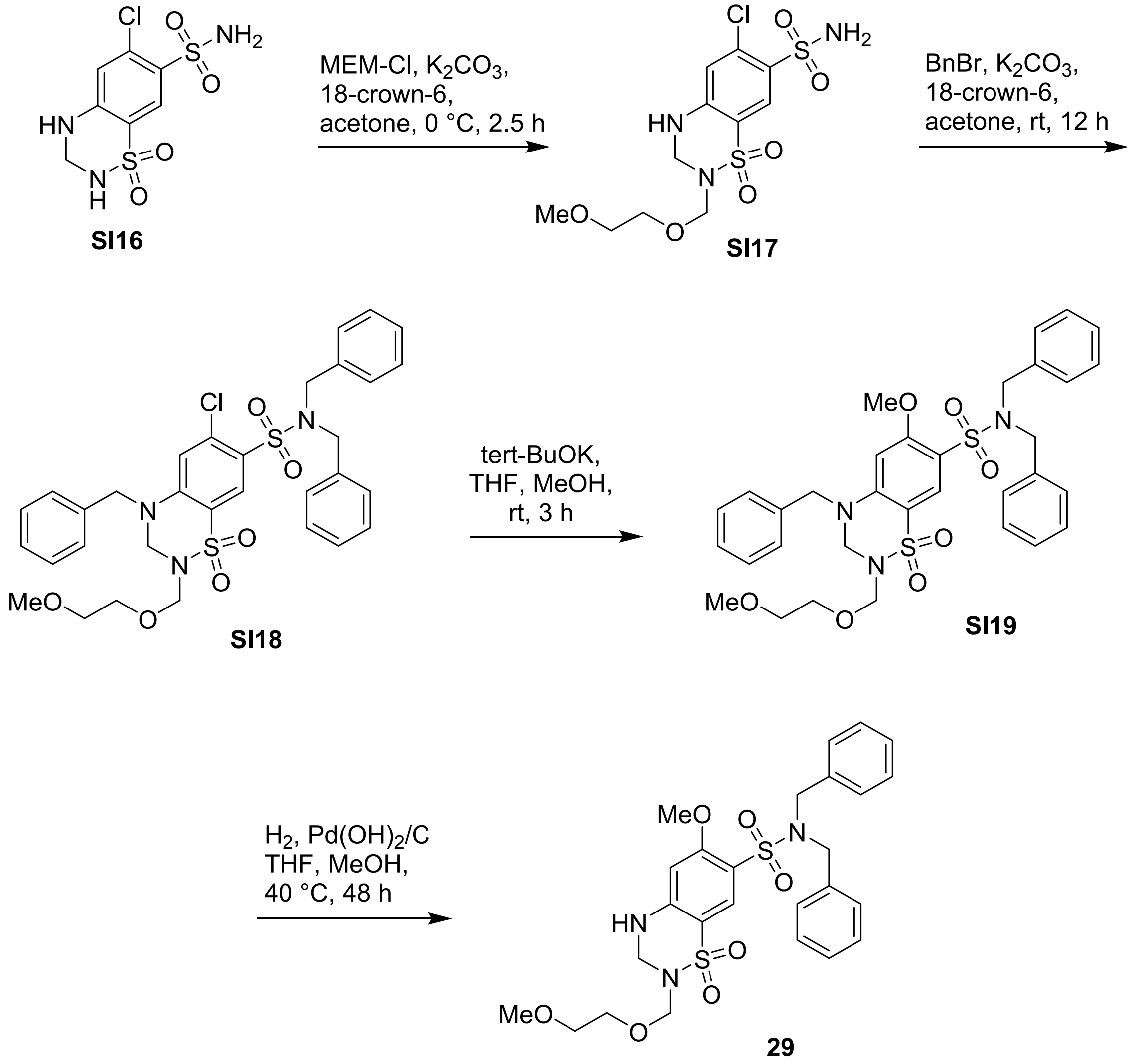

To an ice-cold solution of commercial hydrochlorothiazide (**SI16**, 1.0 g, 3.40 mmol) in acetone (20 mL) was added solid potassium carbonate (2.50 g, 18.0 mmol), 18-crown-6 ether (10.0 mg, 0.037 mmol), and neat MEM-chloride (250 mg, 2.0 mmol). The slurry was vigorously stirred at 0 °C for 1 hour. Then, extra MEM-chloride (190 mg, 1.52 mmol) was added and stirring was continued at 0 °C for 1.5 hour. The reaction mixture was concentrated in vacuum and the evaporation

residue was partitioned between ethyl acetate (40 mL) and water (10 mL). After phase separation, the organic layer was washed with water (3 x 10 mL) and brine. The organic phase was dried over magnesium sulfate, filtered, and concentrate to in vacuum. The tacky evaporation residue was resuspended in a 1:1 (v/v) blend of DCM/diethyl ether (15 mL) and stirred for a while. The slurry was filtered (paper filter, gravity). The solid was collected and dried in vacuum to give **SI17** (1.10 g, 2.850 mmol, 84%) as a white powder.

To a solution of **SI17** (1.10 g, 2.850 mmol) in acetone (30 mL) was added solid potassium carbonate (4.00 g, 28.9 mmol), 18-crown-6 ether (10.0 mg, 0.037 mmol), and benzyl bromide (1.71 g, 10.0 mmol). The slurry was vigorously stirred at room temperature for 12 hours. The reaction mixture was concentrated in vacuum and the evaporation residue was partitioned between ethyl acetate (40 mL) and water (10 mL). After phase separation, the organic layer was washed with water (3 x 10 mL) and brine. The organic phase was dried over magnesium sulfate, filtered, and concentrate to in vacuum. The crude product was purified by column chromatography (EtOAc/hexanes 1:2) to give pure **SI18** (540 mg, 0.820 mmol, 29%).

To a solution of **SI18** (530 mg, 0.810 mmol) in dry THF (10 mL) was added methanol (1.0 mL) and potassium tert-butoxide (90.0 mg, 0.810 mmol). The mixture was stirred at room temperature for 3h. The reaction mixture was concentrated in vacuum and the product was extracted with ethyl acetate. The crude product was purified by column chromatography ($CHCl_3$/methanol 30:1) to

give **SI19** as a tacky solid. The solid was rinsed with diethyl ether and dried in vacuum to give pure **SI19** (500 mg, 0.760 mmol, 95%) as an off-white solid.

SI19 (480 mg, 0.740 mmol) was dissolved in a blend of tetrahydrofuran (10 mL) and methanol (10mL). Palladium hydroxide (20 % on charcoal, 60.0 mg). The reaction mixture was warmed up to 40 °C and stirred under a hydrogen gas atmosphere (rubber balloon) for 48 hours. The reaction mixture was filtered through a celite pad and the clear filtrated was concentrated in vacuum. The crude product was purified by column chromatography ($CHCl_3$/methanol 400:15) to give the pure title compound **29** as a white solid); ^1H NMR ($CDCl_3$, 250 MHz) δ 8.25 (s, 1H), 7.2 (m, 6H), 7.0 - 7.1 (m, 4H), 6.12 (s, 1H), 5.40 (*b*t, 1H), 4.99 (d, 2H, J = 2.9 Hz), 4.67 (s, 2H), 4.36 (s, 4H), 3.7 -3.8 (m, 2H), 3.70 (s, 3H), 3.4 -3.5 (m, 2H), 3.36 (s, 3H); ^{13}C NMR (126 MHz, $CDCl_3$) δ 160.0, 147.5, 135.9, 129.6, 128.42 (2C), 128.37 (2C), 127.6, 119.9, 113.7, 97.7, 78.2, 71.4, 67.9, 58.9, 57.0, 56.1, 50.4.

N,N-dibenzyl-2-methoxypyridine-3-sulfonamide (30)

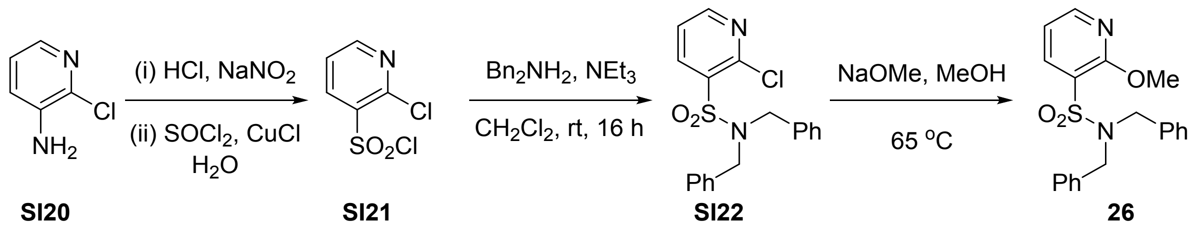

Sulfonyl chloride **SI21** was synthesized following a literature procedure.[4] To a solution of ice/water (25 mL), at 0 °C, was added dropwise thionyl chloride (4.2 mL, 57.90 mmol), keeping the temperature of the mixture below 5 °C. The solution

was then allowed to warm to rt overnight. Copper(I) chloride (15 mg, 0.15 mmol) was added to the mixture, and the resultant greenish solution was cooled to −5 °C using an acetone/ice bath. To a separate flask containing 3-amino-2-chloropyridine **SI20** (1.73 g, 13.46 mmol) was added cautiously hydrochloric acid (36% w/w, 13.5 mL) with stirring, maintaining the temperature of the mixture below 30 °C with ice cooling. After complete addition the reaction mixture was cooled to −5 °C using an ice/acetone bath and a solution of sodium nitrite (1.0 g, 14.50 mmol) in water (4.0 mL) was added dropwise over 45 min, maintaining the temperature of the reaction mixture between −5 to 0 °C, the resultant slurry was cooled to −5 °C and stirred for 10 min. The diazo slurry was then added dropwise (by Pasteur pipette) to the SO_2/CuCl solution over 1h30, keeping the reaction temperature between −5 to 0 °C (the diazo slurry was maintained at −5 °C throughout the addition). After the addition was complete, the resultant precipitate was stirred at 0 °C for a further 1h30 and the filtered. The solid was washed with water (2 × 15 mL) and dried under vacuum to give 2-chloropyridine-3-sulfonyl chloride **SI21** (1.76 g, 8.30 mmol, 62%) as a cream coloured solid.

Following the *General procedure for Sulfonamide formation* of 2-chloropyridine-3-sulfonyl chloride **SI21** (225 mg, 1.06 mmol) and *N,N*-dibenzylamine (209 mg, 1.06 mmol) were reacted for 16 h and purified by column chromatography (10-80% EtOAc in hexane) to give *N,N*-dibenzyl-2-chloropyridine-3-sulfonamide **SI22** (359 mg, 0.963 mmol, 91%) as a colourless solid.

To a solution of *N,N*-dibenzyl-2-chloropyridine-3-sulfonamide **SI22** (300 mg, 0.805 mmol) in MeOH (10 mL) was added sodium methoxide (87 mg, 1.61 mmol)

and the reaction was heated to reflux and stirred for 16 h. The reaction was then concentrated *in vacuo* and partitioned between EtOAc (10 mL) and water (10 mL). The mixture was separated, and the organic phase was dried ($MgSO_4$) and concentrated *in vacuo*. The crude product was purified by column chromatography (10-60% EtOAC in hexane) to give *N,N*-dibenzyl-2-methoxypyridine-3-sulfonamide **30** (223 mg, 0.605 mmol, 75%) as a colourless solid. ^1H NMR (400 MHz, $CDCl_3$) δ = 8.32 (dd, *J* = 1.9, 5.0 Hz, 1H), 8.24 (dd, *J* = 1.8, 7.6 Hz, 1H), 7.26 - 7.19 (m, 6H), 7.09 - 7.03 (m, 4H), 7.00 (dd, *J* = 4.9, 7.6 Hz, 1H), 4.45 (s, 4H), 3.94 (s, 3H); ^{13}C NMR (101 MHz, $CDCl_3$) δ = 159.5 (*C*O), 150.9 (*C*H), 140.0 (*C*H), 135.7 (2C, *C*), 128.4 (8C, *C*H), 127.7 (2C, *C*H), 124.4 (*C*SO_2), 116.6 (*C*H), 54.0 (*C*H_3), 50.6 (2C, *C*H_2); HRMS $C_{20}H_{21}N_2O_3S^+$ calcd. 369.1267, found 369.1271.

_N,N_-bis(4-fluorobenzyl)-1-methyl-2-oxo-1,2-dihydropyridine-3-sulfonamide (31)

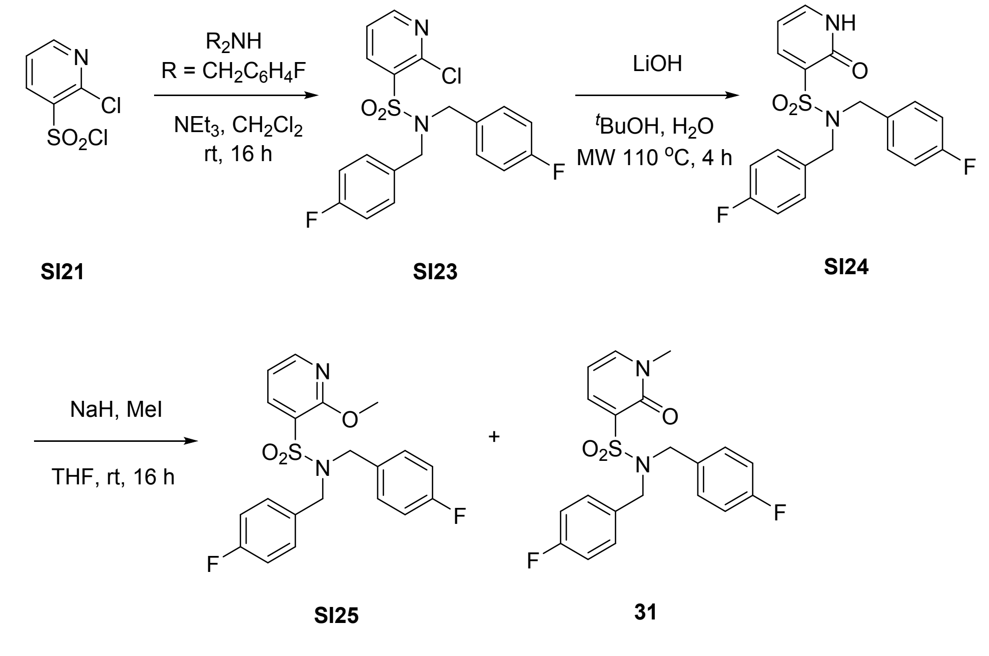

Following the *General procedure for Sulfonamide formation* of 2-chloropyridine-3-sulfonyl chloride **SI21** (500 mg, 2.36 mmol) and bis(4-fluorobenzyl)amine (500 mg, 2.14 mmol) were reacted for 16 h and purified by column chromatography (10-80% EtOAc in hexane) to give 2-chloro-*N,N*-bis(4-fluorobenzyl)pyridine-3-sulfonamide **SI23** (544 mg, 1.33 mmol, 62%) as a colourless solid.

To a MW vial containing a solution of 2-Chloro-*N,N*-bis(4-fluorobenzyl)pyridine-3-sulfonamide **SI23** (300 mg, 0.73 mmol) in ^{t}BuOH/water (3 mL) was added LiOH (123 mg, 5.14 mmol) and the reaction was heated in the microwave for 4 h at 110 ^{o}C. The reaction was concentrated *in vacuo* and then extracted with EtOAc (3 x 5 mL). The combined organics were dried ($MgSO_4$) and concentrated *in vacuo* to give crude *N,N*-bis(4-fluorobenzyl)-2-oxo-1,2-dihydropyridine-3-sulfonamide **SI24** (258 mg, 0.661 mmol, 90%) which was used in the next step without further purification.

To a solution of *N,N*-bis(4-fluorobenzyl)-2-oxo-1,2-dihydropyridine-3-sulfonamide **SI24** (118 mg, 0.302 mmol) in THF (2 mL) at 0 ^{o}C was added NaH (60 wt%, 36 mg, 0.907 mmol) and the mixture was stirred at 0 ^{o}C for a further 15 min and then for 1 h at rt. Iodomethane (100 µL, 1.60 mmol) was added and the reaction was allowed to stir at rt for 16 h. The reaction was then partitioned between EtOAc (10 mL) and water (5 mL). The mixture was separated and the organic phase was dried ($MgSO_4$) and concentrated *in vacuo* to give crude product which was purified by column chromatography (20-100% EtOAc in hexanes) to elute *N,N*-bis(4-fluorobenzyl)-2-methoxypyridine-3-sulfonamide **SI25** (16 mg, 0.040 mmol, 13%); ^{1}H NMR (400 MHz, $CDCl_3$) δ = 8.35 (dd, *J* = 1.9, 5.0 Hz, 1H),

8.23 (dd, J = 1.8, 7.6 Hz, 1H), 7.09 - 6.97 (m, 5H), 6.96 - 6.85 (m, 4H), 4.39 (s, 4H), 3.99 (s, 3H); and pure *N,N*-bis(4-fluorobenzyl)-1-methyl-2-oxo-1,2-dihydropyridine-3-sulfonamide **31** (73 mg, 0.181 mmol, 60%) as a colourless solid; ^{1}H NMR (400 MHz, $CDCl_3$) δ = 8.12 (dd, J = 2.1, 7.2 Hz, 1H), 7.53 (dd, J = 2.0, 6.7 Hz, 1H), 7.14 (dd, J = 5.4, 8.5 Hz, 4H), 6.90 (t, J = 8.7 Hz, 4H), 6.24 (t, J = 6.9 Hz, 1H), 4.52 (s, 4H), 3.58 (s, 3H); HRMS $C_{20}H_{18}F_2N_2O_3SNa^+$ calcd. 427.0898, found 427.0907.

Compounds from Table 2

N,N-bis(4-fluorobenzyl)-2,5-dimethoxybenzenesulfonamide (32)

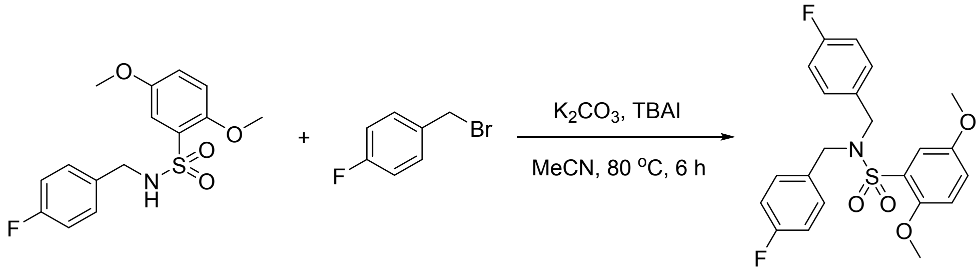

SI26 **32**

To a solution of *N*-(4-fluorobenzyl)-2,5-dimethoxybenzenesulfonamide **SI26** (40 mg, 0.123 mmol) in MeCN (1 mL) was added 4-fluorobenzyl bromide (20 μL, 0.150 mmol), tetrabutylammonium iodide (4 mg, 0.010 mmol), and potassium carbonate (41 mg, 0.300 mmol). The reaction was heated to 80 °C and stirred at this temperature until the reaction was complete (6 h), as observed by tlc. The reaction was allowed to cool rt and water (5 mL) was added. The mixture was extracted with EtOAc (2 x 5 mL), dried ($MgSO_4$) and concentrated *in vacuo* to give crude product which was purified by column chromatography (10-40% EtOAc in hexanes) to give pure *N,N*-bis(4-fluorobenzyl)-2,5-

dimethoxybenzenesulfonamide **32** (51 mg, 0.115 mmol, 93%) as a colourless solid; ^{1}H NMR (400 MHz, $CDCl_3$) δ = 7.56 (d, *J* = 3.1 Hz, 1H), 7.10 (dd, *J* = 3.2, 8.9 Hz, 1H), 7.05 - 6.97 (m, 4H), 6.97 - 6.85 (m, 5H), 4.36 (s, 4H), 3.83 (s, 3H), 3.79 (s, 3H); ^{19}F NMR (470 MHz, $CDCl_3$) δ = -114.74 (s, 1F); ^{13}C NMR (101 MHz, $CDCl_3$) δ = 162.2 (d, *J* = 245.9 Hz, *C*F); 153.1 (*C*O), 150.7 (*C*O), 131.8 (2C, d, *J* = 3.0 Hz, *C*), 130.2 (4C, d, *J* = 8.0 Hz, *C*H), 129.5 (CSO_2), 120.3 (*C*H), 115.6 (*C*H), 115.2 (4C, d, *J* = 22.1 Hz, *C*H), 113.6 (C*H*), 56.4 (CH_3), 56.1 (CH_3), 49.9 (2C, CH_2); HRMS $C_{22}H_{22}F_2NO_4S^+$ calcd. 434.1232, found 434.1236.

_N_-(4-fluorobenzyl)-_N_-(4-fluorophenyl)-2,5-dimethoxybenzenesulfonamide (33)

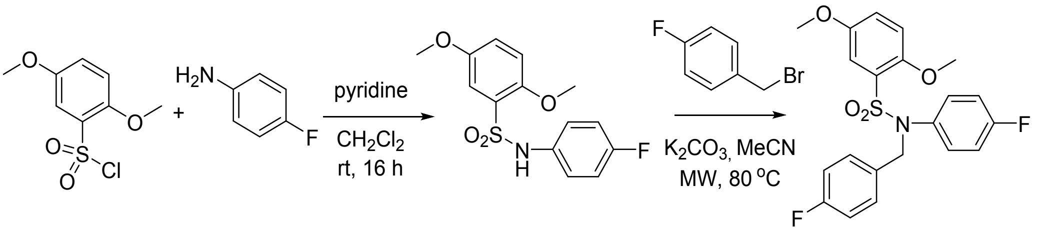

SI27 33

To a solution of 4-fluoroaniline (50 mg, 0.450 mmol) in CH_2Cl_2 (2 mL) was added pyridine (150 μL, 1.80 mmol) followed by 2,5-dimethoxybenzenesulfonyl chloride (128 mg, 0.540 mmol) and the reaction was stirred at rt for 16 h. The reaction was concentrated *in vacuo* and then re-dissolve with EtOAc (10 mL) and then washed with 1 M HCl (2 x 10 mL) and 2 M NaOH (5 mL). The organic was then dried ($MgSO_4$) and concentrated *in vacuo* to give crude product which was purified by column chromatography (10-80% EtOAc in hexanes) to give *N*-(4-fluorophenyl)-2,5-dimethoxybenzenesulfonamide **SI27** (40 mg, 0.128 mmol, 29%). The sulfonamide **SI27** was re-dissolved in MeCN (1 mL) and transferred to

a MW vial. To the reaction solution was added 4-fluorobenzyl bromide (50 μL, 0.402 mmol), followed by potassium carbonate (53 mg, 0.384 mmol) and the reaction was heated in the MW at 80 °C for 1 h. The reaction was then concentrated *in vacuo* and partitioned between EtOAc (5 mL) and water (5 mL). The mixture was separated and the organic phase was dried ($MgSO_4$) and concentrated *in vacuo* to give crude product which was purified by column chromatography (10-60% EtOAc in hexanes) to give pure *N*-(4-fluorobenzyl)-*N*-(4-fluorophenyl)-2,5-dimethoxybenzenesulfonamide **33** (48 mg, 0,114 mmol, 89%) as an off-white solid; ^{1}H NMR (400 MHz, $CDCl_3$) δ = 7.26 - 7.18 (m, 3H), 7.10 - 7.04 (m, 1H), 7.03 - 6.99 (m, 1H), 6.95 (ddd, *J* = 1.7, 6.8, 8.9 Hz, 4H), 6.90 - 6.81 (m, 2H), 4.90 (s, 2H), 3.97 (s, 3H), 3.71 (s, 3H); HRMS $C_{21}H_{20}F_2NO_4S^+$ calcd. 420.1076, found 420.1070.

N-((2,4-dimethoxypyrimidin-5-yl)methyl)N-(4-fluorobenzyl)-2,5-dimethoxybenzenesulfonamide (34)

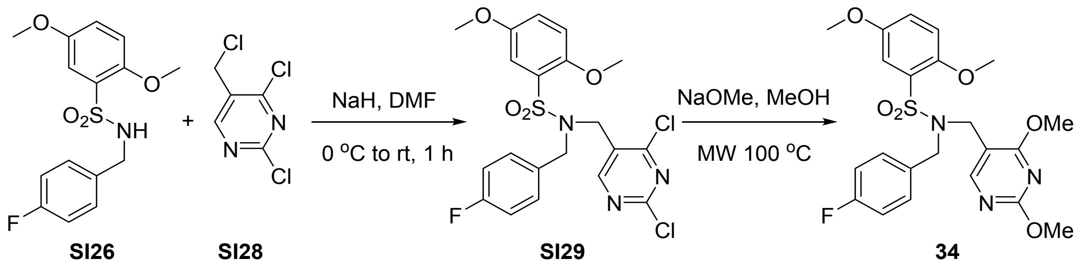

To a solution of *N*-(4-fluorobenzyl)-2,5-dimethoxybenzenesulfonamide **SI26** (150 mg, 0.461 mmol) in DMF (2 mL) at 0 °C was added NaH (60 wt%, 20 mg, 0.507 mmol) and the reaction was stirred at this temperature for 15 min and then allowed to warm to rt and stirred for 1 h. The reaction was cooled to 0 °C again and a solution of 2,4-dichloro-5-(chloromethyl)pyrimidine **SI28** (100 mg, 0.507 mmol)[5]

in DMF (0.5 mL) was added in one portion. The reaction was stirred at 0 °C for a further 1 h and then quenched with water (10 mL). The reaction was extracted with EtOAc (2 x 10 mL) and the combined organics were dried ($MgSO_4$) and concentrated *in vacuo*. The crude product was then purified by column chromatography (10-80% EtOAc in hexanes) to give *N*-((2,4-dichloropyrimidin-5-yl)methyl)-*N*-(4-fluorobenzyl)-2,5-dimethoxybenzenesulfonamide **SI29** (133 mg, 0.273 mmol, 59%). This was then re-dissolved in MeOH (2 mL) and transferred to a MW vial. Sodium methoxide (150 mg, 2.78 mmol) was added and the reaction was heated to 100 °C for 2 h. The reaction was concentrated *in vacuo* and partitioned between EtOAc (10 mL) and water (5 mL). The mixture was separated and the organic phase was washed with sat. brine (5 mL), dried ($MgSO_4$) and concentrated *in vacuo*. The crude product was then purified by column chromatography (20-100% EtOAc in hexanes) to give pure *N*-((2,4-dimethoxypyrimidin-5-yl)methyl)N-(4-fluorobenzyl)-2,5-dimethoxybenzenesulfonamide **34** (84 mg, 0.176 mmol, 64%) as an off-white solid; ^{1}H NMR (500 MHz, $CDCl_3$) δ = 8.00 (s, 1H), 7.37 (d, *J* = 3.0 Hz, 1H), 7.20 (dd, *J* = 5.3, 8.5 Hz, 2H), 7.03 (dd, *J* = 3.1, 9.0 Hz, 1H), 6.96 (t, *J* = 8.6 Hz, 2H), 6.90 (d, *J* = 9.1 Hz, 1H), 4.51 (s, 2H), 4.26 (s, 2H), 3.93 (s, 3H), 3.85 (s, 3H), 3.80 (s, 3H), 3.79 (s, 3H); HRMS $C_{22}H_{25}FN_3O_6S^+$ calcd. 478.1443, found 478.1455.

N-(4-fluorobenzyl)-2,5-dimethoxy-N-((1-methyl-1H-pyrazol-5-yl)methyl)benzenesulfonamide (35)

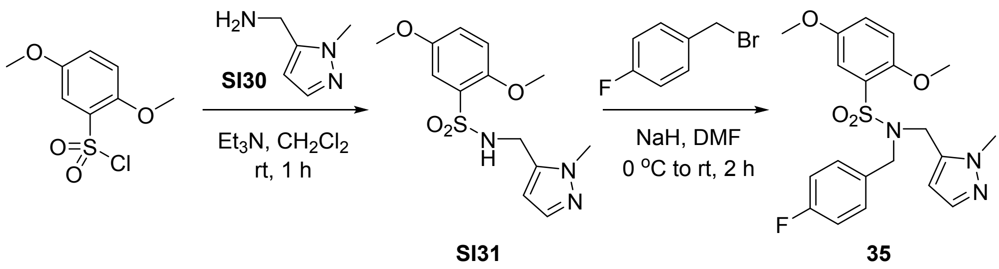

To a solution of (1-methyl-1H-pyrazol-5-yl)methanamine **SI30** (110 mg, 1.00 mmol)[6] in CH_2Cl_2 (5 mL) at 0 °C was added triethylamine (140 µL, 2.00 mmol) followed by 2,5-dimethoxybenzenesulfonyl chloride (237 mg, 1.00 mmol) and the reaction was stirred for 1 h at rt. The reaction was diluted with more in CH_2Cl_2 (5 mL) and washed with water (5 mL), dried ($MgSO_4$) and concentrated *in vacuo* to give crude 2,5-dimethoxy-*N*-((1-methyl-1H-pyrazol-5-yl)methyl)benzenesulfon-amide **SI31** (289 mg, 0.928 mmol, 93%) which was used in the next step without further purification.

To a solution of 2,5-dimethoxy-*N*-((1-methyl-1H-pyrazol-5-yl)methyl)benzene-sulfonamide **SI31** (100 mg, 0.321 mmol) in DMF (2 mL) at 0 °C was added NaH (60 wt%, 20 mg, 0.507 mmol) and the reaction was stirred at this temperature for 15 min and then allowed to warm to rt and stirred for 1 h. The reaction was cooled to 0 °C again and 4-fluorobenzyl bromide (50 µL, 0.400 mmol) was added. The reaction was stirred at 0 °C for a further 2 h and then quenched with water (10 mL). The reaction was extracted with EtOAc (2 x 10 mL) and the combined organics were dried ($MgSO_4$) and concentrated *in vacuo*. The crude product was then purified by column chromatography (20-100% EtOAc in hexanes) to give pure *N*-

(4-fluorobenzyl)-2,5-dimethoxy-N-((1-methyl-1H-pyrazol-5-yl)methyl)benzenesulfonamide **35** (77 mg, 0.183 mmol, 57%) as an off-white solid; ^{1}H NMR (500 MHz, $CDCl_3$) δ = 7.52 (d, *J* = 3.1 Hz, 1H), 7.31 (d, *J* = 1.7 Hz, 1H), 7.11 (dd, *J* = 3.1, 9.1 Hz, 1H), 7.01 - 6.87 (m, 5H), 5.95 (d, *J* = 1.7 Hz, 1H), 4.46 (s, 2H), 4.35 (s, 2H), 3.85 (s, 3H), 3.83 (s, 3H), 3.66 (s, 3H); HRMS $C_{20}H_{23}FN_3O_4S^+$ calcd. 420.1388, found 420.1369.

N-(4-fluorobenzyl)-2,5-dimethoxy-*N*-((3-methyl-1,2,4-oxadiazol-5-yl)methyl)benzenesulfonamide (36)

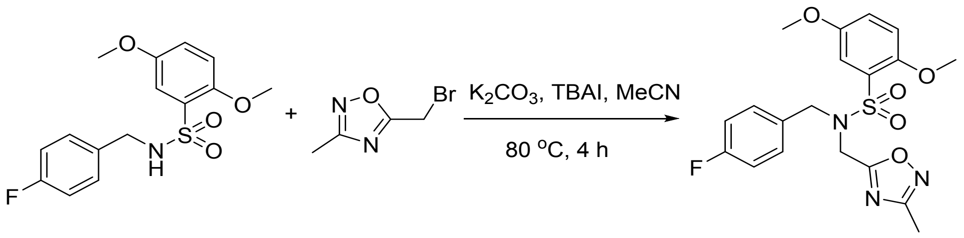

SI26 **SI32** **36**

To a solution of 5-(bromomethyl)-3-methyl-1,2,4-oxadiazole **SI32** (39 mg, 0.220 mmol) in MeCN (1 mL) was added N-(4-fluorobenzyl)-2,5-dimethoxybenzenesulfonamide **SI26** (60 mg, 0.184 mmol), potassium carbonate (51 mg, 0.369 mmol) and tetrabutylammonium iodide (6.8 mg, 18 μmol). The reaction was heated to reflux and stirred for 4 h. Afterwards, the reaction was allowed to cool to rt and diluted with water (2 mL). The reaction was extracted with EtOAc (2 x 5 mL), dried (MgSO4) and concentrated to give crude product which was purified by column chromatography (20-40% EtOAc in hexanes) to *N*-(4-fluorobenzyl)-2,5-dimethoxy-*N*-((3-methyl-1,2,4-oxadiazol-5-yl)methyl)

benzenesulfonamide **36** (41 mg, 0.097 mmol, 53%) as a pale yellow oil; ^{1}H NMR (400 MHz, $CDCl_3$) δ 7.48 (d, *J* = 3.1 Hz, 1H), 7.25 – 7.19 (m, 2H), 7.07 (dd, *J* = 9.0, 3.1 Hz, 1H), 6.98 (t, *J* = 8.6 Hz, 2H), 6.90 (d, *J* = 9.0 Hz, 1H), 4.62 (s, 2H), 4.58 (s, 2H), 3.81 (s, 3H), 3.79 (s, 3H), 2.27 (s, 3H); ^{13}C NMR (101 MHz, $CDCl_3$) δ 174.8 (*C*), 167.1 (*C*), 162.6 (d, *J* = 247.0 Hz, *C*), 153.0 (*C*), 150.8 (*C*), 130.9 (2C, d, *J* = 3.3 Hz, *C*), 130.6 (2C, d, *J* = 8.2, *C*H), 128.1 (*C*), 120.8 (*C*H), 115.57 (2C, d, *J* = 21.5 Hz, *C*H), 115.56 (*C*H), 113.5 (*C*H), 56.4 (CH_3), 56.1 (CH_3), 51.2 (CH_2), 41.5 (CH_2), 11.4 (CH_3); HRMS $C_{19}H_{21}N_3O_5SF^+$ calcd. 422.1180, found 422.180.

_N_-(4-fluorobenzyl)-2,5-dimethoxy-_N_-methylbenzenesulfonamide (37)

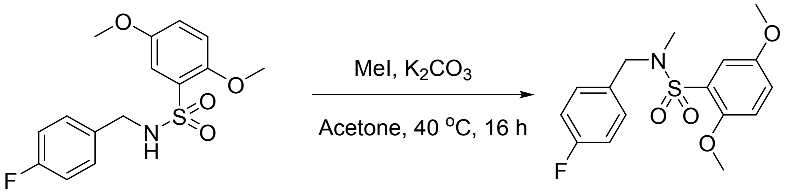

To a solution of *N*-(4-fluorobenzyl)-2,5-dimethoxybenzenesulfonamide (50 mg, 0.154 mmol) in acetone (1 mL) was added iodomethane (50 μL, 0.750 mmol), and potassium carbonate (41 mg, 0.300 mmol). The reaction was heated to 40 °C and stirred at this for 16 h. The reaction was allowed to cool rt and water (5 mL) was added. The mixture was extracted with EtOAc (2 x 5 mL), dried ($MgSO_4$) and concentrated *in vacuo* to give crude product which was purified by column chromatography (10-40% EtOAc in hexanes) to give pure *N*-(4-fluorobenzyl)-2,5-dimethoxy-*N*-methylbenzenesulfonamide **37** (36 mg, 0.106 mmol, 69%) as a colourless oil; ^{1}H NMR (400 MHz, $CDCl_3$) δ = 7.52 (d, *J* = 3.2 Hz, 1H), 7.34 - 7.28 (m, 2H), 7.08 (dd, *J* = 3.1, 9.0 Hz, 1H), 7.05 - 6.95 (m, 3H), 4.32 (s, 2H), 3.90 (s, 3H), 3.82 (s, 3H), 2.72 (s, 3H).

N-benzyl-*N*-ethyl-2,5-dimethoxybenzenesulfonamide (38)

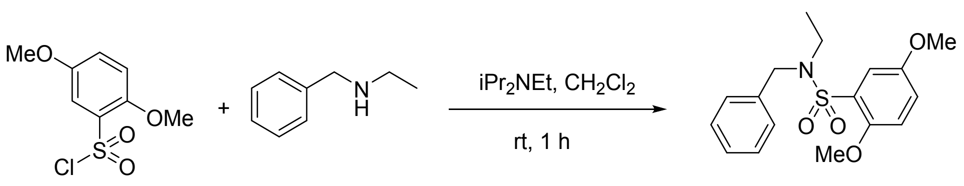

Following the *General procedure for Sulfonamide formation* 2,5-dimethoxybenzenesulfonyl chloride (230 mg, 1.00 mmol) and *N*-benzylethanamine (135 mg, 1.00 mmol) were reacted for 1 h. After extraction with EtOAc and concentration to dryness in vacuum, the crude product was purified by column chromatography [short path silica gel (ca. 4 grams), neat chloroform] to give pure *N*-benzyl-*N*-ethyl-2,5-dimethoxybenzenesulfonamide **38** (280 mg, 0.835 mmol, 83%) as a pale, viscous liquid; ^{1}H NMR ($CDCl_3$, 400 MHz) δ 7.52 (d, 1H, *J* = 3.2 Hz), 7.2-7.3 (m, 6H), 7.04 (dd, 1H, *J* = 3.2, 9.0 Hz), 7.00 (m, 4H), 6.93 (d, 1H, *J* = 9.0 Hz), 4.49 (s, 2H), 3.86 (s, 3H), 3.80 (s, 3H), 3.25 (q, 2H, *J* = 7.2 Hz), 0.91 (t, 2H, *J* = 7.2 Hz); ^{13}C NMR (126 MHz, $CDCl_3$) δ 152.9, 150.7, 137.2, 129.6, 128.4 (2C), 128.1 (2C), 127.4, 120.0, 115.7, 113.6, 56.5, 56.0, 51.0, 41.9, 13.1.

N-benzyl-2,5-dimethoxy-*N*-(2-methoxyethyl)benzenesulfonamide (39)

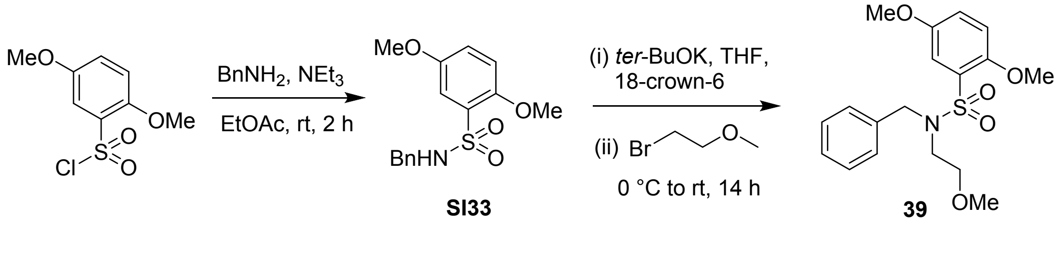

A solution of 2,5-dimethoxybenzensulfonyl chloride (710 mg, 3.00 mmol) in ordinary ethyl acetate (10 mL) was added of a mixture of triethylamine (404 mg, 4.00 mmol), benzylamine (320 mg, 3.00 mmol), and ethyl acetate (5 mL) at 0 °C.

The ice bath was removed, and the clear solution was stirred for 2 hours at room temperature. The reaction mixture was diluted with ethyl acetate (25 mL), washed with 1N HCl solution, saturated $NaHCO_3$ solution, and brine. The organic phase was dried over $MgSO_4$, filtered, and concentrated *in vacuo* to give **SI33** (920, 3.00 mmol, 100%) as an oil which solidified to an off-white solid on standing.

Sulfonamide **SI33** (160 mg, 0.520 mmol) was then dissolved in dry THF (6 mL) under an argon atmosphere. The solution was cooled down to 0 °C to receive the addition of solid potassium tert-butoxide (76.0 mg, 0.600 mmol) and 18-crown-6 ether (*ca.* 5 mg). The slurry was stirred for 2 hours at 0 °C. Neat 1-bromo-2-methoxyethane (80.0 mg, 0.60 mmol) was added, the ice bath was removed, and the mixture was stirred overnight. THF was evaporated in vacuum. The crude product was extracted with ethyl acetate and purified by column chromatography (20-40% EtOAc/hexanes) to give *N*-benzyl-2,5-dimethoxy-*N*-(2-methoxyethyl)benzenesulfonamide **39** as a pale oil (180 mg, 0.490 mmol, 95%); ^{1}H NMR ($CDCl_3$, 400 MHz) δ 7.52 (d, 1H, J = 3.2 Hz), 7.2-7.3 (m, 5H), 7.04 (dd, 1H, J = 3.2, 9.0 Hz), 6.92 (m, 1H), 4.55 (s, 2H), 3.84 (s, 3H), 3.79 (s, 3H), 3.39 (t, 2H, J = 6.0 Hz), 3.28 (t, 2H, J = 6.0 Hz), 3.11 (s, 3H); ^{13}C NMR (126 MHz, $CDCl_3$) δ 153.1, 150.9, 137.1, 129.5, 128.4 (2C), 128.3 (2C), 127.5, 120.1, 115.7, 113.6, 71.2, 58.6, 56.5, 56.1, 52.8, 46.7.

N-(4-fluorobenzyl)-N-(2-methoxyethyl)-2-(trifluoromethoxy)benzenesulfonamide (40)

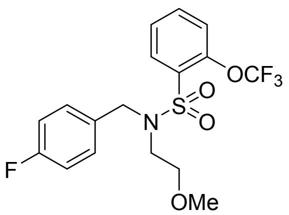

Following the *General procedure for Sulfonamide formation* 2-trifluoromethoxybenzenesulfonyl chloride (50 mg, 0.192 mmol) and *N*-(4-fluorobenzyl)-2-methoxyethan-1-amine (37 mg, 0.200 mmol) were reacted for 16 h and purified by column chromatography (10-60% EtOAc in hexane) to give pure *N*-(4-fluorobenzyl)-*N*-(2-methoxyethyl)-2-(trifluoromethoxy)benzenesulfonamide **40** (73 mg, 0.179 mmol, 93%) □as a pale yellow oil; ^{1}H NMR (250 MHz, $CDCl_3$) δ = 8.06 (dd, *J* = 1.7, 7.9 Hz, 1H), 7.66 - 7.49 (m, 1H), 7.45 - 7.17 (m, 6H), 4.59 (s, 2H), 3.43 - 3.32 (m, 2H), 3.29 - 3.22 (m, 2H), 3.05 (s, 3H); HRMS $C_{17}H_{19}F_4NO_4S^+$ calcd. 408.0887, found 408.0891.

_N_-(4-fluorobenzyl)-2-methoxy-_N_-(2-methoxyethyl)-5-(morpholine-4-carbonyl)benzenesulfonamide (42)

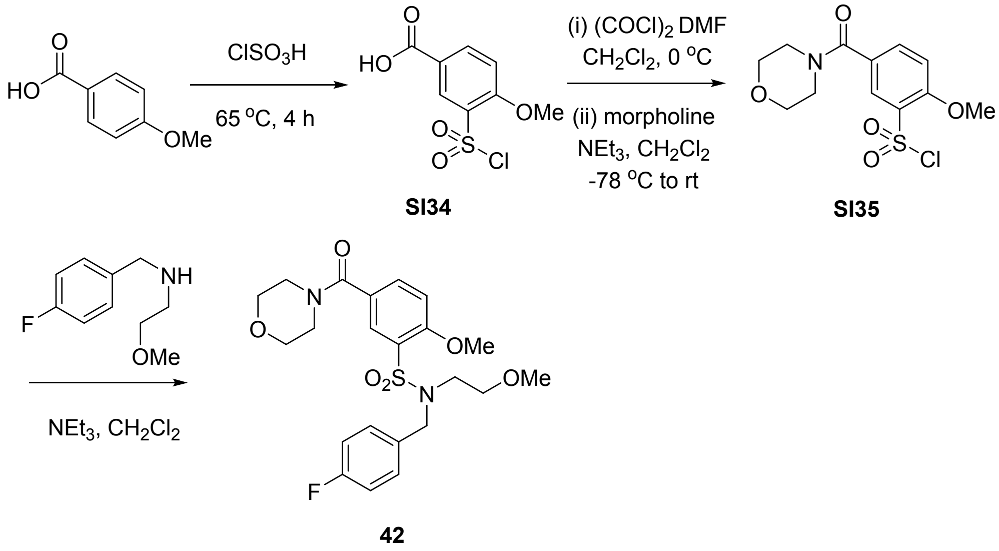

4-Methoxybenzoic acid (2.21 g, 14.53 mmol) was added portionwise to a solution of chlorosulfonic acid (7.7 mL, 116.2 mmol) at 0 °C. The reaction was stirred at 0 °C for 15 min and then warmed to 65 °C and stirred for 4 h. The solution was then cooled to 0 °C and added slowly to ice/water (50 mL), forming a precipitate, and then stirred for 15 min at rt. The white suspension was then extracted with EtOAc (60 mL). The organic phase was dried ($MgSO_4$) and concentrated in vacuo to give 3-(chlorosulfonyl)-4-methoxybenzoic acid **SI34** (2.36 g, 9.42 mmol, 65%) as a light pink solid. The solid was dissolved in CH_2Cl_2 (40 mL) and cooled to 0 °C. Oxalyl chloride (1.2 mL, 14.13 mmol) was added dropwise, followed by 1 drop of DMF. The reaction was allowed to warm to rt and stirred for 3 h and then concentrated *in vacuo*. The crude acyl chloride was re-dissolved in CH_2Cl_2 (40 mL) and cooled to -78 °C. Triethylamine (2.6 mL, 18.84 mmol) was added and the reaction stirred for a further 5 min at -78 °C. To this was then added dropwise

morpholine (0.81 mL, 9.42 mmol) and the reaction was stirred for 30 min at -78 °C and then allowed to warm to rt over 1 h. The reaction was washed with water (2 x 10 mL), dried ($MgSO_4$) and concentrated *in vacuo* to give crude amide which was purified by column chromatography to give pure 2-methoxy-5-(morpholine-4-carbonyl)benzenesulfonyl chloride **SI35** (2.65 g, 8.29 mmol, 88%) as a colourless solid.

Following the *General procedure for Sulfonamide formation* 2-methoxy-5-(morpholine-4-carbonyl)benzenesulfonyl chloride **SI35** (50 mg, 0.156 mmol) and *N*-(4-fluorobenzyl)-2-methoxyethan-1-amine (32 mg, 0.175 mmol) were reacted for 16 h and purified by column chromatography (20-100% EtOAc in hexane) to give pure *N*-(4-fluorobenzyl)-2-methoxy-*N*-(2-methoxyethyl)-5-(morpholine-4-carbonyl)benzenesulfonamide **42** (57 mg, 0.122 mmol, 78%) □as an off-white solid; ^{1}H NMR (250 MHz, $CDCl_3$) δ = 8.03 (d, *J* = 2.2 Hz, 1H), 7.68 (dd, *J* = 2.1, 8.5 Hz, 1H), 7.31 - 7.18 (m, 2H), 7.06 (d, *J* = 8.5 Hz, 1H), 7.03 - 6.93 (m, 2H), 4.55 (s, 2H), 3.96 (s, 3H), 3.72 (br m, 8H), 3.44 - 3.33 (m, 2H), 3.33 - 3.23 (m, 2H), 3.11 (s, 3H); HRMS $C_{22}H_{28}FN_2O_6S^+$ calcd. 467.1647, found 467.1644.

5-cyano-*N*-(4-fluorobenzyl)-2-methoxy-*N*-(tetrahydro-2H-pyran-4-yl)benzenesulfonamide (43)

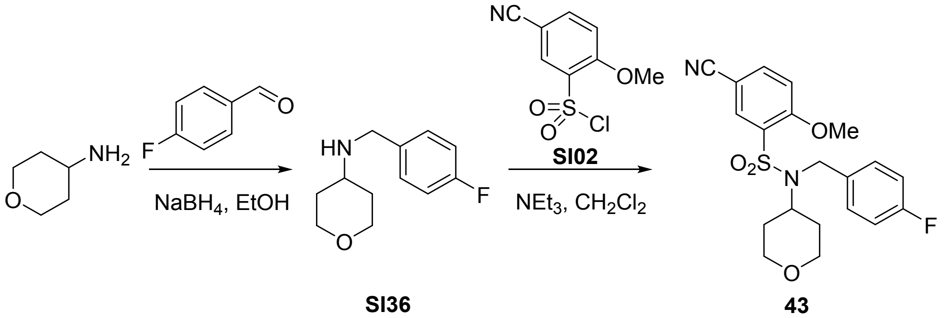

To a solution of 4-fluorobenzaldehyde (210 µL, 1.98 mmol) in EtOH (4 mL) was added 4-aminomethyltetrahydropyran (205 µL, 1.98 mmol). The reaction was heated to 80 °C, stirred for 2 h and then cooled to 0 °C. Sodium borohydride (150 mg, 3.96 mmol) was added portionwise and the reaction was stirred at 0 °C for 1 h and then allowed to warm to rt and stirred overnight. The reaction was concentrated *in vacuo* and then partitioned between Et_2O (10 mL) and water (10 mL). The mixture was separated and the organic phase was washed with sat. brine (10 mL), dried ($MgSO_4$) and concentrated *in vacuo* to give crude *N*-(4-fluorobenzyl)tetrahydro-2H-pyran-4-amine **SI36** (221 mg, 1.06 mmol, 53%) as a pale yellow oil.

Following the *General procedure for Sulfonamide formation* 2-methoxy-5-cyano-benzenesulfonyl chloride **SI02** (50 mg, 0.216 mmol) and *N*-(4-fluorobenzyl)tetrahydro-2H-pyran-4-amine **SI36** (52 mg, 0.250 mmol) were reacted for 16 h and purified by column chromatography (20-100% EtOAc in hexane) to give pure 5-cyano-*N*-(4-fluorobenzyl)-2-methoxy-*N*-(tetrahydro-2H-pyran-4-yl)benzenesulfonamide **43** (41 mg, 0.101 mmol, 47%) □as an off-white solid; ^{1}H NMR (400 MHz, $CDCl_3$) δ = 8.16 (d, *J* = 2.1 Hz, 1H), 7.79 (dd, *J* = 2.1, 8.7 Hz, 1H), 7.36 - 7.29 (m, 2H), 7.07 (d, *J* = 8.7 Hz, 1H), 7.02 - 6.94 (m, 2H), 4.51 (s, 2H), 4.05 (s, 3H), 4.02 - 3.93 (m, 1H), 3.90 (dd, *J* = 4.5, 11.6 Hz, 2H), 3.32 (dt, *J* = 1.8, 11.9 Hz, 2H), 1.66 (dq, *J* = 4.6, 12.3 Hz, 2H), 1.45 (br dd, *J* = 2.0, 12.3 Hz, 2H); HRMS $C_{20}H_{22}FN_2O_4S^+$ calcd. 405.1279, found 405.12.

N-(4-fluorobenzyl)-N-isobutyl-2-methoxy-5-(morpholine-4-carbonyl)benzenesulfonamide (44)

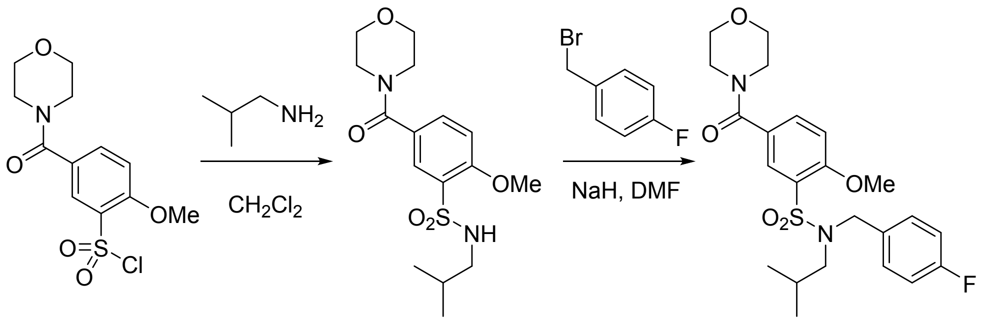

SI35 **SI37** **44**

To a solution of isobutylamine (50 μL, 0.503 mmol) in CH_2Cl_2 (1 mL) at 0 °C was added 2-methoxy-5-(morpholine-4-carbonyl)benzenesulfonyl chloride **SI35** (50 mg, 0.156 mmol) and the reaction was stirred for 1 h. The reaction was diluted with EtOAc (5 mL) and washed with 1 M HCl (2 mL) and then sat. brine (5 mL). The organic phase was dried ($MgSO_4$) and concentrated *in vacuo* to give crude *N*-isobutyl-2-methoxy-5-(morpholine-4-carbonyl)benzenesulfonamide **SI37** (53 mg, 0.149 mmol, 95%) as a pale yellow solid which was used in the next step without further purification. The isobutylsulfonamide **SI37** was re-dissolved in DMF (1 mL), cooled to 0 °C and NaH (60wt%, 10 mg, 0.260 mmol) was added. The mixture was stirred for 1 h and then 4-fluorobenzyl bromide (20 μL, 0.161 mmol) was added and the reaction was allowed to stir for 2 h. The reaction was quenched with water (5 mL) and extracted with EtOAc (10 mL). The organic was washed with sat. brine (5 mL), dried ($MgSO_4$) and concentrated *in vacuo* to give crude product which was purified by column chromatography (20-100% EtOAc in hexanes) to give pure *N*-(4-fluorobenzyl)-N-isopentyl-2-methoxy-5-(morpholine-4-carbonyl)benzenesulfonamide **44** (38 mg, 0.082 mmol, 55%) as an off-white

solid; ^{1}H NMR (400 MHz, $CDCl_3$) δ = 8.03 (d, *J* = 2.1 Hz, 1H), 7.68 (dd, *J* = 2.1, 8.5 Hz, 1H), 7.22 (dd, *J* = 5.4, 8.6 Hz, 2H), 7.05 (d, *J* = 8.6 Hz, 1H), 6.98 (t, *J* = 8.6 Hz, 2H), 4.44 (s, 2H), 3.95 (s, 3H), 3.72 (br m, 8H), 2.98 (d, *J* = 7.6 Hz, 2H), 1.75 - 1.62 (m, 1H), 0.73 (d, *J* = 6.6 Hz, 6H); HRMS $C_{23}H_{30}FN_2O_5S^+$ calcd. 465.1854, found 465.1861.

N-(4-fluorobenzyl)-2-methoxy-5-(morpholine-4-carbonyl)-N-((6-(trifluoromethyl)pyridin-3-yl)methyl)benzenesulfonamide (46)

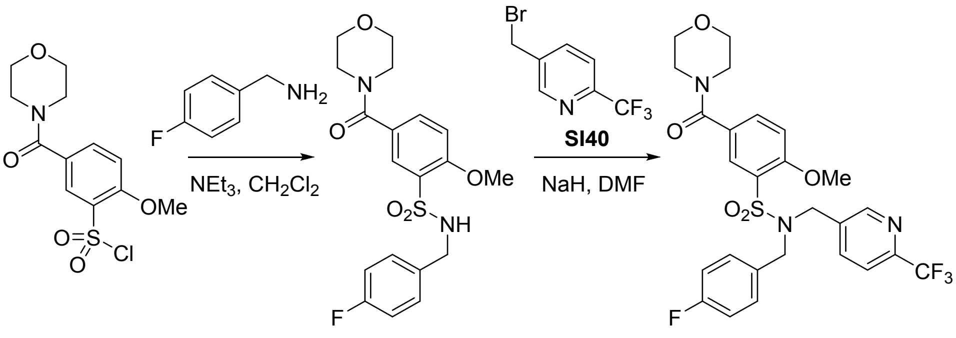

SI35 SI39 46

To a solution of 2-methoxy-5-(morpholine-4-carbonyl)benzenesulfonyl chloride **SI35** (300 mg, 0.94 mmol) in CH_2Cl_2 (10 mL) at 0 °C was added triethylamine (195 µL, 1.41 mmol), followed by 4-fluorobenzylamine (160 µL, 1.41 mmol). The reaction was stirred at 0 °C for a further 15 min and then allowed to warm to rt and stirred for 4 h. The reaction was quenched 2 M NaOH and stirred for 15 min. The mixture was separated, and the resultant organic phase was washed with 1 M HCl (10 mL), dried ($MgSO_4$), and concentrated in vacuo to give crude N-(4-fluorobenzyl)-2-methoxy-5-(morpholine-4-carbonyl)benzenesulfonamide **SI39** (318 mg, 0.78 mmol, 83%) as an off-white solid which was used in the next step without further purification.

To a suspension of sodium hydride (60wt%, 12 mg, 0.313 mmol) in DMF (0.7 mL) at 0 °C was added N-(4-fluorobenzyl)-2-methoxy-5-(morpholine-4-carbonyl)benzenesulfonamide **SI39** (61 mg, 0.149 mmol) and the reaction was stirred for 15 min at 0 °C and a further 45 min at rt. The reaction was then cooled to 0 °C again and 5-(bromomethyl)-2-(trifluoromethyl)pyridine **SI40** (48 mg, 0.2 mmol) was added in one portion. The reaction was stirred at 0 °C for 15 min and then allowed to warm to rt and stirred for a further 1 h. Cooled to 0 °C again and water (10 mL) added. The mixture was extracted with EtOAc (2 x 5 mL). The combined organics were washed with sat. brine (5 mL), dried ($MgSO_4$) and concentrated *in vacuo* to give crude product which was purified by column chromatography (20-100% EtOAc in hexanes) to give pure *N*-(4-fluorobenzyl)-2-methoxy-5-(morpholine-4-carbonyl)-*N*-((6-(trifluoromethyl)pyridin-3-yl)methyl) benzenesulfonamide **46** (49 mg, 0.086 mmol, 58%) as an off-white solid; ^{1}H NMR (250 MHz, $CDCl_3$) δ = 8.37 (br d, *J* = 1.8 Hz, 1H), 8.10 (d, *J* = 2.1 Hz, 1H), 7.71 (dd, *J* = 1.9, 8.4 Hz, 2H), 7.60 - 7.48 (m, 1H), 7.09 (d, *J* = 8.6 Hz, 1H), 7.04 - 6.96 (m, 2H), 6.94 - 6.83 (m, 2H), 4.50 (s, 2H), 4.37 (s, 2H), 3.95 (s, 3H), 3.89 - 3.41 (m, 8H); HRMS $C_{26}H_{26}F_4N_3O_5S^+$ calcd. 568.1524, found 568.1529.

N-benzyl-2-methoxy-5-(morpholine-4-carbonyl)-*N*-((2-(trifluoromethyl)pyrimidin-5-yl)methyl)benzenesulfonamide (47)

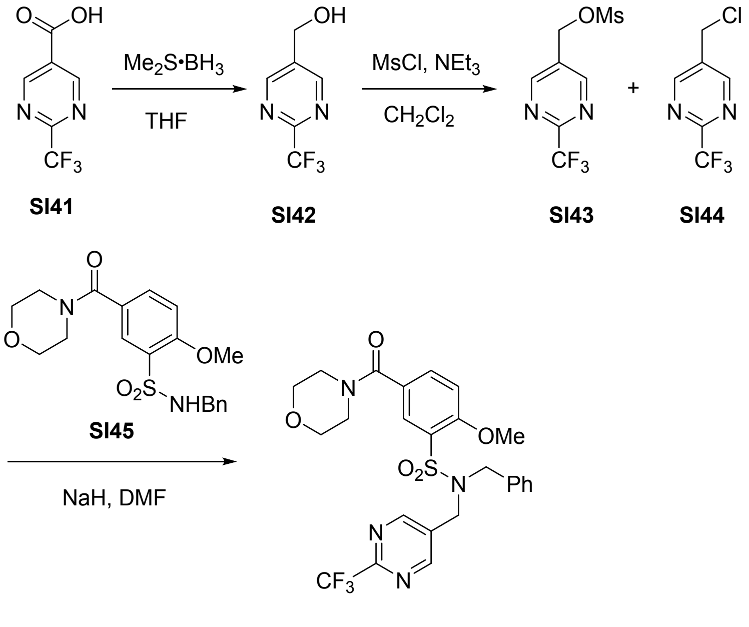

To a solution of 2-(trifluoromethyl)pyrimidine-5-carboxylic acid **SI41** (250 mg, 1.30 mmol) in THF (5 mL) at 0 °C was added dropwise borane dimethyl sulfide complex (2 M in THF, 1.3 mL, 2.60 mmol) over 5 min. The reaction was stirred at 0 °C for a further 15 min and then allowed to warm to rt and stirred overnight. The reaction was cooled to 0 °C and MeOH (3 mL) was added cautiously. The reaction was allowed to warm to rt and stirred for 30 min and then concentrated. The residue was partitioned between EtOAc (10 mL) and 0.5 M NaOH (5 mL) and sat. brine (1 mL). The mixture was separated and the organic phase was dried ($MgSO_4$) and concentrated in vacuo to give crude (2-(trifluoromethyl)pyrimidin-5-yl)methanol **SI42** (70wt% in EtOAc based on NMR, 120 mg, 0.48 mmol, 37%). The crude alcohol was re-dissolved in CH_2Cl_2 (2 mL) at 0 °C triethylamine (133 μL, 0.96 mmol) and methanesulfonyl chloride (56 μL, 0.72 mmol) were added.

The reaction was stirred at 0 °C for 5 min and then allowed to warm to rt and stirred for 2 h. Quenched with sat. $NaHCO_3$ (5 mL), separated and re-extracted with more DCM (2 x 5 mL). The combined organics were dried ($MgSO_4$) and concentrated in vacuo to give crude product. The organic phase was dried ($MgSO_4$) and concentrated in vacuo to give a 1:1 mixture of (2-(trifluoromethyl)pyrimidin-5-yl)methyl methanesulfonate **SI43** and 5-(chloromethyl)-2-(trifluoromethyl) pyrimidine **SI44** (105 mg, ~0.45 mmol) which was used in the next step without further purification.

To a suspension of sodium hydride (60wt%, 12 mg, 0.313 mmol) in DMF (0.7 mL) at 0 °C was added N-benzyl-2-methoxy-5-(morpholine-4-carbonyl)benzene sulfonamide **SI45** (60 mg, 0.154 mmol) and the reaction was stirred for 15 min at 0 °C and a further 45 min at rt. The reaction was then cooled to 0 °C again and a solution of the mesylate/chloride mixture of **SI43** and **SI44** (1:1, 40 mg, 0.20 mmol) in DMF (0.3 mL) was added in one portion. The reaction was stirred at 0 °C for 15 min and then allowed to warm to rt and stirred for a further 1 h. The reaction was cooled to 0 °C again and water (10 mL) added. The mixture was extracted with EtOAc (2 x 5 mL). The combined organics were washed with sat. brine (5 mL), dried ($MgSO_4$) and concentrated *in vacuo* to give crude product which was purified by column chromatography (40 EtOAc in hexanes to 1% MeOH in EtOAc) to give pure *N*-benzyl-2-methoxy-5-(morpholine-4-carbonyl)-*N*-((2-(trifluoromethyl)pyrimidin-5-yl)methyl)benzenesulfonamide **47** (38 mg, 0.069 mmol, 45%) as an off-white solid; ^{1}H NMR (250 MHz, DMSO-d6) δ = 8.68 (s, 2H), 7.87 (d, *J* = 2.1 Hz, 1H), 7.72 (dd, *J* = 2.2, 8.5 Hz, 1H), 7.28 (d, *J* = 8.6

Hz, 1H), 7.22 - 7.13 (m, 5H), 4.61 (s, 2H), 4.50 (s, 2H), 3.97 (s, 3H), 3.71 - 3.40 (m, 8H); HRMS $C_{25}H_{26}F_3N_4O_5S^+$ calcd. 551.1571, found 551.1565.

2-Methoxy-5-(morpholine-4-carbonyl)-*N*-neopentyl-*N*-((6-(trifluoromethyl)pyridin-3-yl)methyl)benzenesulfonamide (48)

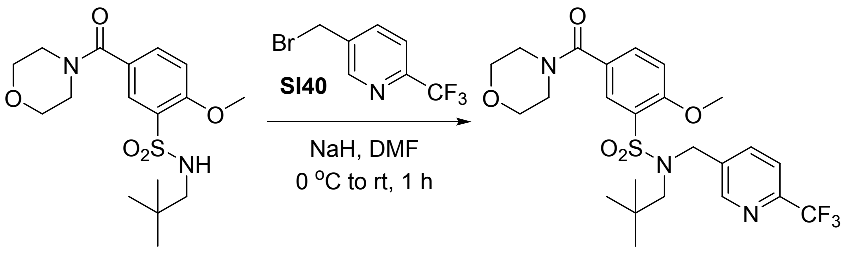

SI38 48

To a suspension of sodium hydride (60wt%, 12 mg, 0.313 mmol) in DMF (0.7 mL) at 0 °C was added 2-methoxy-5-(morpholine-4-carbonyl)-N-neopentylbenzene-sulfonamide **SI38** (60 mg, 0.162 mmol) and the reaction was stirred for 15 min at 0 °C and a further 45 min at rt. The reaction was then cooled to 0 °C again and 5-(bromomethyl)-2-(trifluoromethyl)pyridine **SI40** (48 mg, 0.2 mmol) was added in one portion. The reaction was stirred at 0 °C for 15 min and then allowed to warm to rt and stirred for a further 1 h. The reaction was cooled to 0 °C again and water (10 mL) added. The mixture was extracted with EtOAc (2 x 5 mL). The combined organics were washed with sat. brine (5 mL), dried ($MgSO_4$) and concentrated *in vacuo* to give crude product which was purified by column chromatography (20-100% EtOAc in hexanes) to give pure 2-methoxy-5-(morpholine-4-carbonyl)-*N*-neopentyl-*N*-((6-(trifluoromethyl)pyridin-3-yl)methyl)benzenesulfonamide **48** (55 mg, 0.104 mmol, 64%) as an off-white solid; ^1H NMR (250 MHz, $CDCl_3$) δ = 8.61 - 8.48 (m, 1H), 8.00 (d, *J* = 2.1 Hz, 1H), 7.93 - 7.85 (m, 1H), 7.69 - 7.57 (m,

2H), 7.02 (d, J = 8.5 Hz, 1H), 4.71 (s, 2H), 3.98 (s, 3H), 3.87 - 3.44 (br m, 8H), 3.15 (s, 2H), 0.87 (s, 9H).

***N*-Benzyl-5-cyano-2-methoxy-*N*-((2-(trifluoromethyl)pyrimidin-5-yl)methyl)benzenesulfonamide (49)**

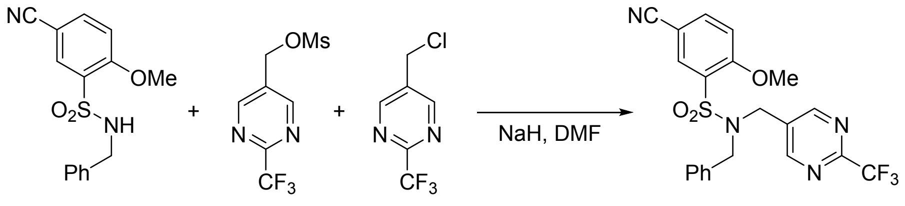

SI46 **SI43** **SI44** **49**

To a suspension of sodium hydride (60wt%, 10 mg, 0.260 mmol) in DMF (0.5 mL) at 0 °C was added N-benzyl-5-cyano-2-methoxybenzenesulfonamide **SI46** (40 mg, 0.132 mmol) and the reaction was stirred for 15 min at 0 °C and a further 45 min at rt. The reaction was then cooled to 0 °C again and a solution of the mesylate/chloride mixture of **SI43** and **SI44** (1:1, 40 mg, 0.20 mmol) in DMF (0.3 mL) was added in one portion. The reaction was stirred at 0 °C for 15 min and then allowed to warm to rt and stirred for a further 1 h. The reaction was cooled to 0 °C again and water (10 mL) added. The mixture was extracted with EtOAc (2 x 5 mL). The combined organics were washed with sat. brine (5 mL), dried ($MgSO_4$) and concentrated *in vacuo* to give crude product which was purified by column chromatography (40% EtOAc in hexanes to 1% MeOH in EtOAc) to give pure *N*-Benzyl-5-cyano-2-methoxy-*N*-((2-(trifluoromethyl)pyrimidin-5-yl)methyl)benzenesulfonamide **49** (32 mg, 0.069 mmol, 52%) as an off-white solid; ^1H NMR (250 MHz, $CDCl_3$) δ = 8.61 (s, 2H), 8.32 (d, J = 2.1 Hz, 1H), 7.89

(dd, $J = 2.1, 8.7$ Hz, 1H), 7.25 - 7.18 (m, 3H), 7.15 (d, $J = 8.7$ Hz, 1H), 7.08 - 6.98 (m, 2H), 4.53 (s, 2H), 4.40 (s, 2H), 4.03 (s, 3H).

5-Cyano-2-methoxy-*N,N*-bis((6-(trifluoromethyl)pyridin-3-yl)methyl)benzenesulfonamide (50)

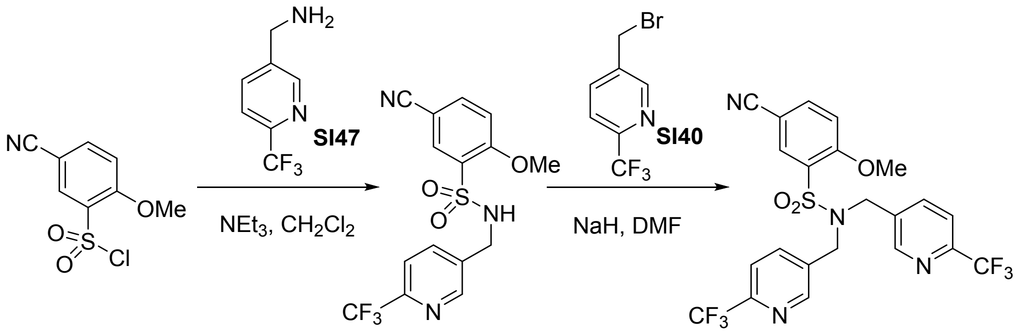

SI02 SI48 50

To a solution of (6-(trifluoromethyl)pyridin-3-yl)methanamine **SI47** (100 mg, 0.568 mmol) in CH_2Cl_2 (3 mL) at 0 °C was added triethylamine (210 µL, 1.50 mmol) followed by 5-cyano-2-methoxybenzenesulfonyl chloride **SI02** (130 mg, 0.561 mmol). The reaction was stirred at 0 °C for a further 15 min and then allowed to warm to rt and stirred for 4 h. The reaction was quenched 2 M NaOH and stirred for 15 min. The mixture was extracted with EtOAc (2 x 5 mL) and the combined organics were washed with 1 M HCl (5 mL), dried ($MgSO_4$), and concentrated in vacuo to give crude 5-cyano-2-methoxy-N-((6-(trifluoromethyl)pyridin-3-yl)methyl)benzenesulfonamide **SI48** (201 mg, 0.541 mmol, 96%) as a yellow solid which was used in the next step without further purification.

To a suspension of sodium hydride (60wt%, 15 mg, 0.391 mmol) in DMF (0.7 mL) at 0 °C was added 5-cyano-2-methoxy-N-((6-(trifluoromethyl)pyridin-3-yl)methyl)benzenesulfonamide **SI48** (70 mg, 0.189 mmol) and the reaction was

stirred for 15 min at 0 °C and a further 45 min at rt. The reaction was then cooled to 0 °C again and a solution of 5-(bromomethyl)-2-(trifluoromethyl)pyridine **SI40** (48 mg, 0.2 mmol) in DMF (0.3 mL) was added in one portion. The reaction was stirred at 0 °C for 15 min and then allowed to warm to rt and stirred for a further 1 h. The reaction was cooled to 0 °C again and water (10 mL) added. The mixture was extracted with EtOAc (2 x 5 mL). The combined organics were washed with sat. brine (5 mL), dried ($MgSO_4$) and concentrated *in vacuo* to give crude product which was purified by column chromatography (40% EtOAc in hexanes to 1% MeOH in EtOAc) to give pure 5-Cyano-2-methoxy-*N,N*-bis((6-(trifluoromethyl)pyridin-3-yl)methyl)benzenesulfonamide **50** (46 mg, 0.087 mmol, 46%) as an off-white solid; ^1H NMR (250 MHz, $CDCl_3$) δ = 8.41 (d, *J* = 1.8 Hz, 2H), 8.27 (d, *J* = 2.1 Hz, 1H), 7.88 (dd, *J* = 2.1, 8.7 Hz, 1H), 7.71 (dd, *J* = 2.1, 8.1 Hz, 2H), 7.55 (d, *J* = 8.1 Hz, 2H), 7.15 (d, *J* = 8.8 Hz, 1H), 4.56 (s, 4H), 4.05 (s, 3H); HRMS $C_{22}H_{17}F_6N_4O_3S^+$ calcd. 531.0920, found 531.0921.

1 H. Jiang, X. Tang, Z. Xu, H. Wang, K. Han, X. Yang, Y. Zhou, Y. L. Feng, X. Y. Yu and Q. Gui, *Org. Biomol. Chem.*, **2019**, *17*, 2715–2720.

2 A. F. Kornahrens, A. B. Cognetta, D. M. Brody, M. L. Matthews, B. F. Cravatt and D. L. Boger, *J. Am. Chem. Soc.*, **2017**, 139, 7052–7061.

3 W. Zhu and D. Ma, *J. Org. Chem.*, **2005**, 70, 2696–2700.

4 P. J. Hogan and B. G. Cox, *Org. Process Res. Dev.*, **2009**, 13, 875–879.

5 H. Cho, I. Shin, E. Ju, S. Choi, W. Hur, H. Kim, E. Hong, N. D. Kim, H. G. Choi, N. S. Gray and T. Sim, *J. Med. Chem.*, **2018**, 61, 8353–8383.

6 Gardell, S.; Pinkerton, A. B.; Sergienko, E.; Sessions, H. Small Molecule Activators of Nicotinamide Phosphoribosyltransferase (Nampt) and Uses Thereof. WO2018132372A1, July 19, **2018**.

NMR Spectra

DESSOY MAD1631 CDCl3 400 MHz set13madH1

Acquisition Time (sec)	2.0447	***Comment***	DESSOY MAD1631 CDCl3 400 MHz set13madH1			***Date***	13 Sep 2017 18:04:02
File Name	\\nmrsparc.iqm.unicamp.br\espectros\avance400\2017\set17\Reserva\Luiz Carlos\set13madH1_001001r					***Frequency (MHz)***	400.18
Nucleus	1H	***Number of Transients***	16	***Original Points Count***	16384	***Points Count***	65536
Pulse Sequence	zg30	***Solvent***	CHLOROFORM-D	***Sweep Width (Hz)***	8012.82	***Temperature (degree C)***	25.167

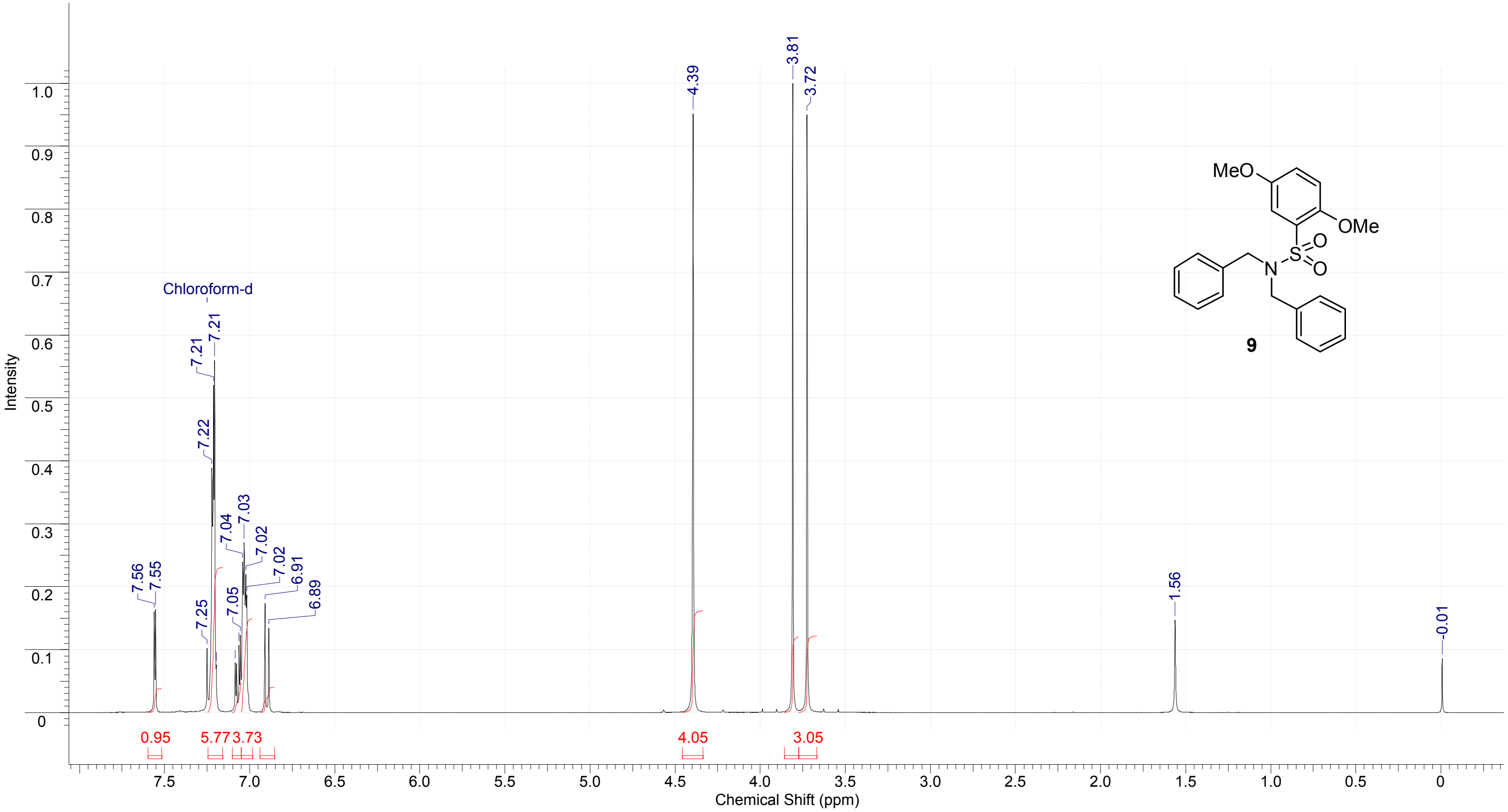

Dessoy - MAD 1631 - CDCl3 - Avance 500 MHz - set18madC1 - 13C

Acquisition Time (sec)	0.4981	***Comment***	Dessoy - MAD 1631 - CDCl3 - Avance 500 MHz - set18madC1 - 13C	***Date***	18 Sep 2017 14:13:00
File Name	\\nmrsparc.iqm.unicamp.br\espectros\avance500\2017\set17\Reserva\Luiz Carlos\set18madC1_001001r			***Frequency (MHz)***	125.69
Nucleus	13C	***Number of Transients*** 512	***Original Points Count*** 16384	***Points Count***	32768
Pulse Sequence	zgpg30	***Solvent*** CHLOROFORM-D	***Sweep Width (Hz)*** 32894.74	***Temperature (degree C)***	27.156

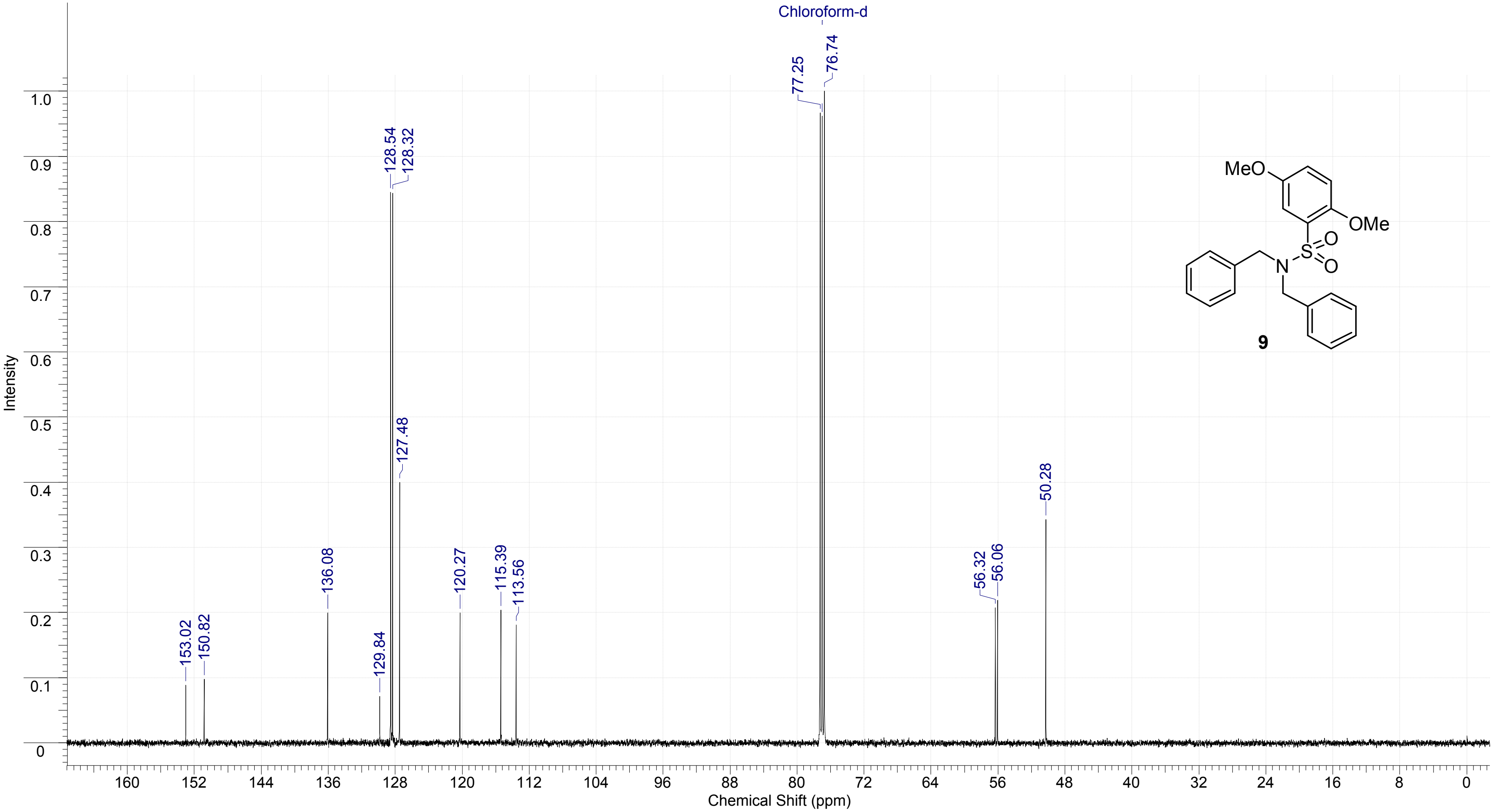

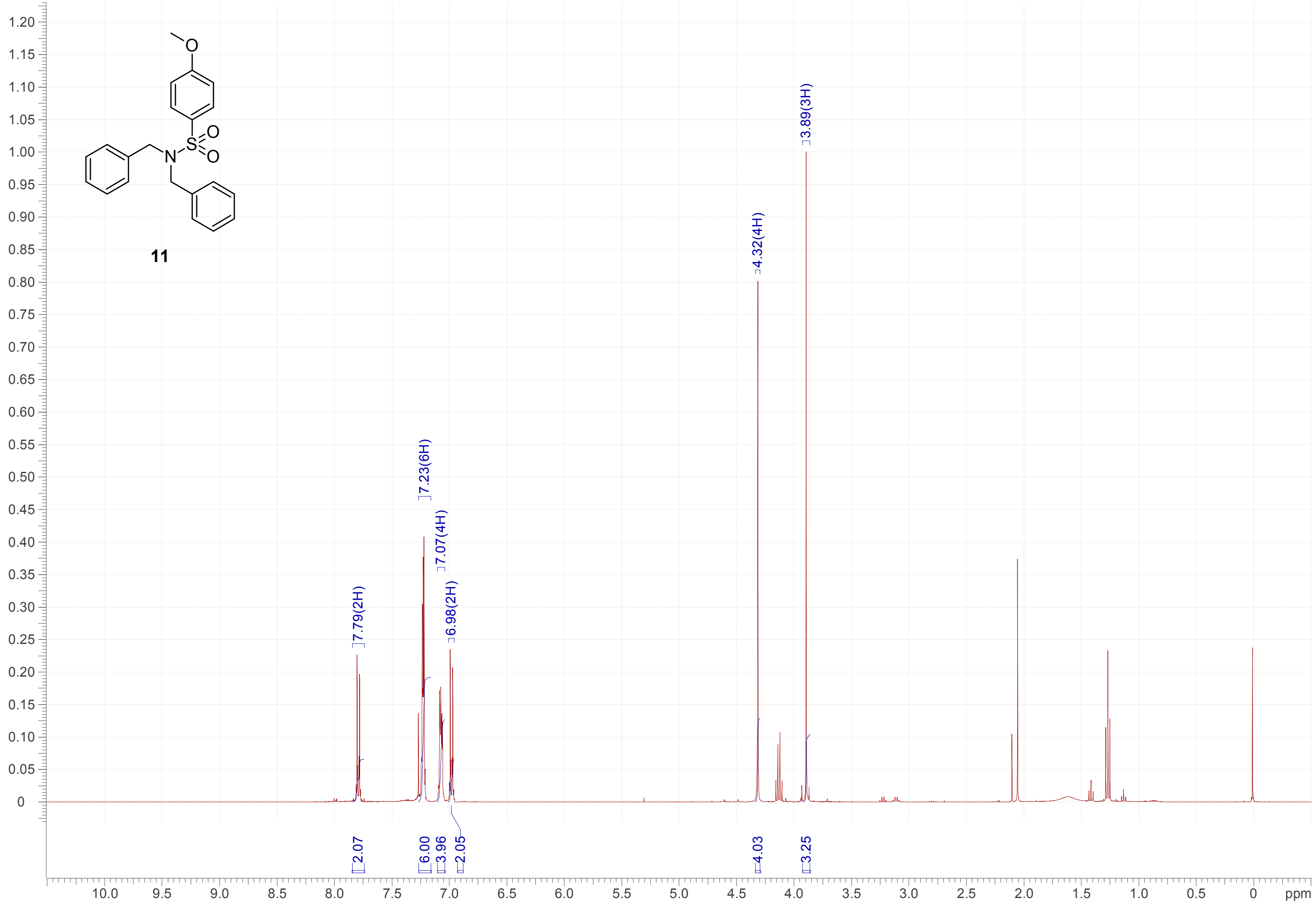

11
7.79(2H)
7.23(6H)
7.07(4H)
6.98(2H)
4.32(4H)
3.89(3H)
2.07
6.00
3.96
2.05
4.03
3.25
ppm

Dessoy - MAD1746 - CDCl3 - Avance 400 MHz - mai23madH1 - 1H

Acquisition Time (sec)	2.0447	***Comment***	Dessoy - MAD1746 - CDCl3 - Avance 400 MHz - mai23madH1 - 1H			***Date***	23 May 2018 17:46:58
File Name	\\nmrsparc.iqm.unicamp.br\espectros\avance400\2018\mai18\Reserva\Luiz Carlos\mai23madH1_001001r					***Frequency (MHz)***	400.18
Nucleus	1H	***Number of Transients***	16	***Original Points Count***	16384	***Points Count***	65536
Pulse Sequence	zg30	***Solvent***	CHLOROFORM-D	***Sweep Width (Hz)***	8012.82	***Temperature (degree C)***	25.360

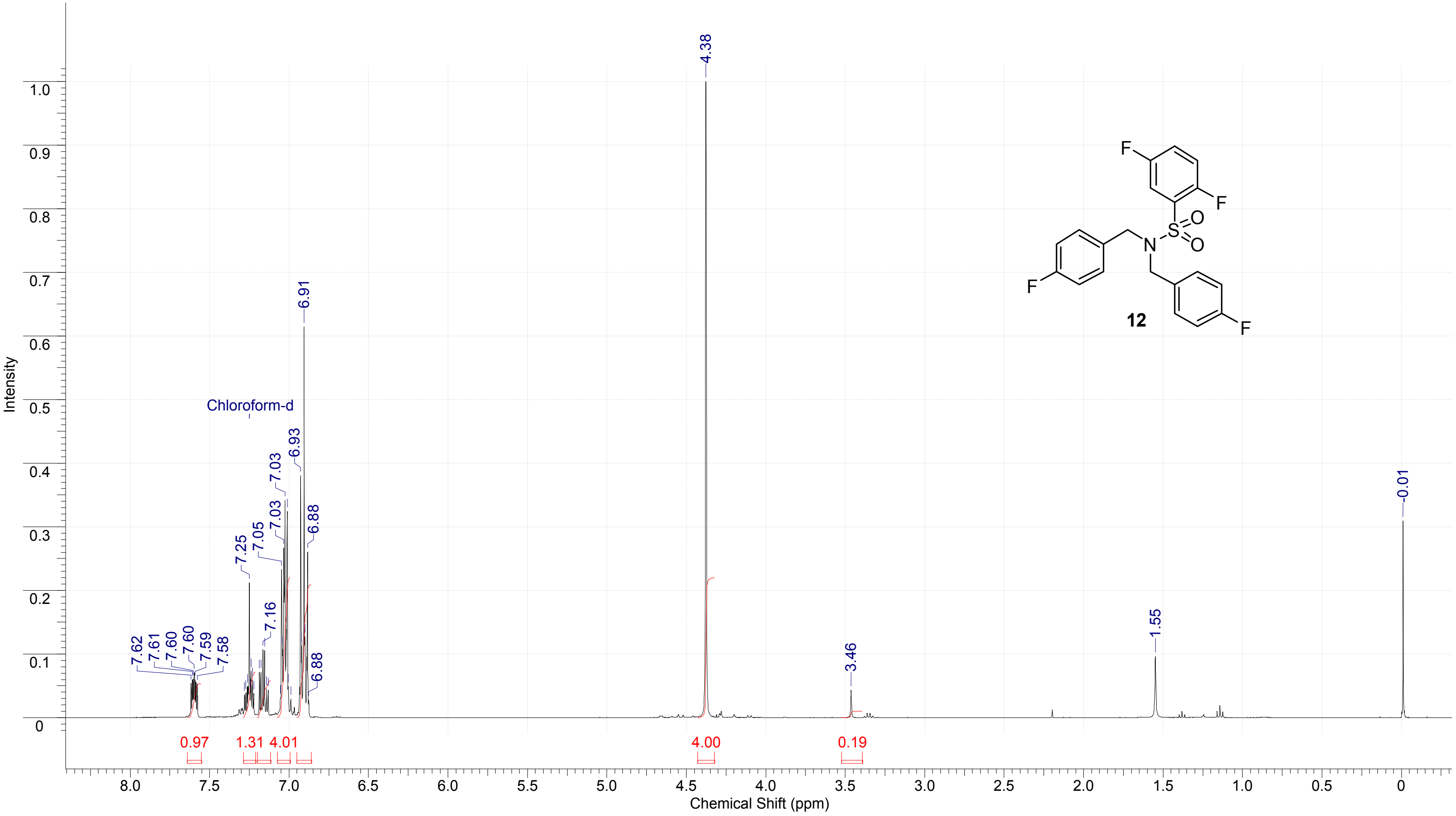

Dessoy - MAD1746 - CDCl3 - Av 600 MHz - mai25madC1

Acquisition Time (sec)	0.4522	***Comment***	Dessoy - MAD1746 - CDCl3 - Av 600 MHz - mai25madC1			***Date***	25 May 2018 08:38:02
File Name	\\nmrsparc.iqm.unicamp.br\espectros\avance600\2018\mai18\Sala\Luiz Carlos\mai25madC1_001001r					***Frequency (MHz)***	150.91
Nucleus	13C	***Number of Transients***	1024	***Original Points Count***	16384	***Points Count***	65536
Pulse Sequence	zgpg30	***Solvent***	CHLOROFORM-D	***Sweep Width (Hz)***	36231.88	***Temperature (degree C)***	25.152

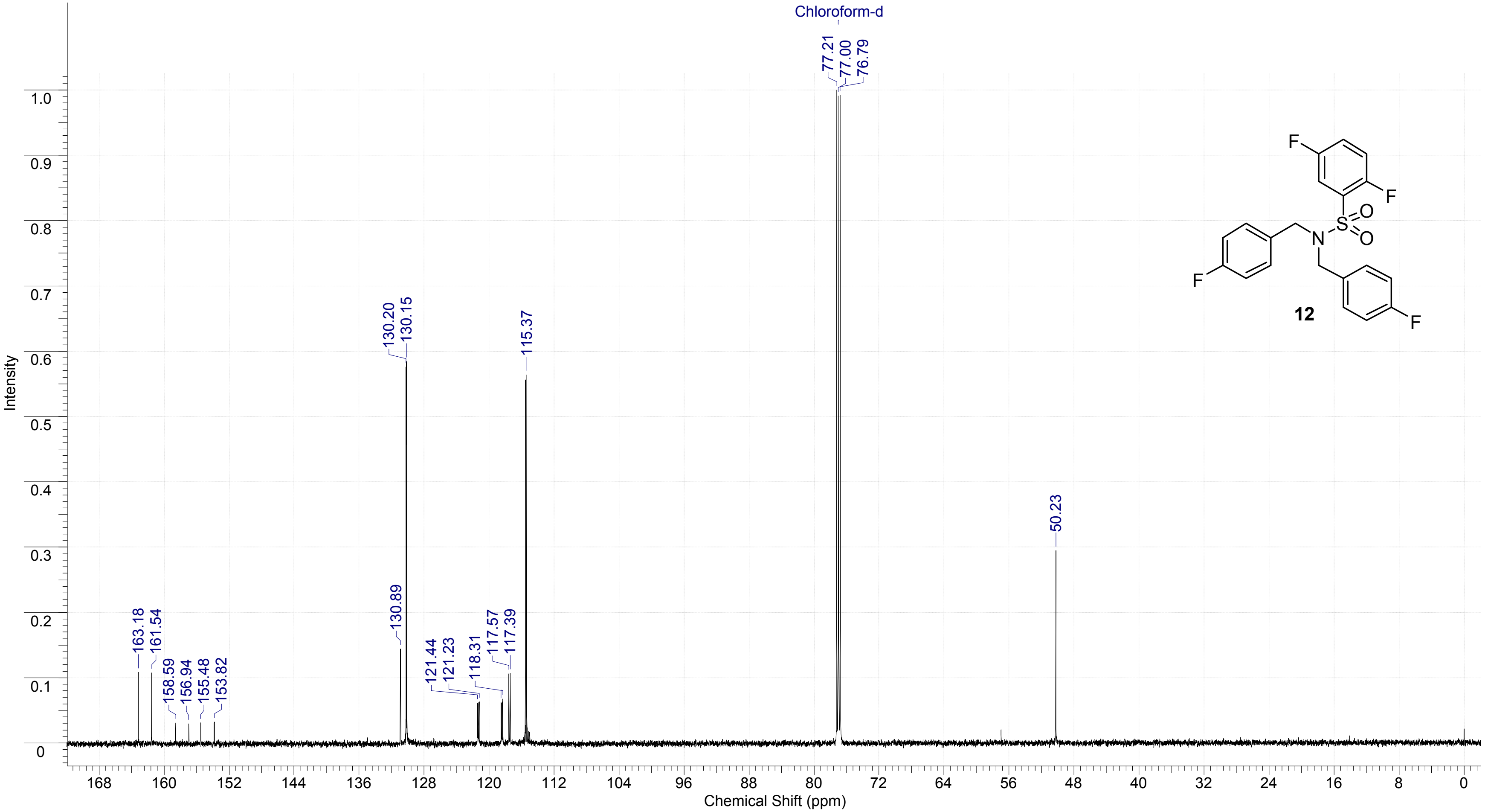

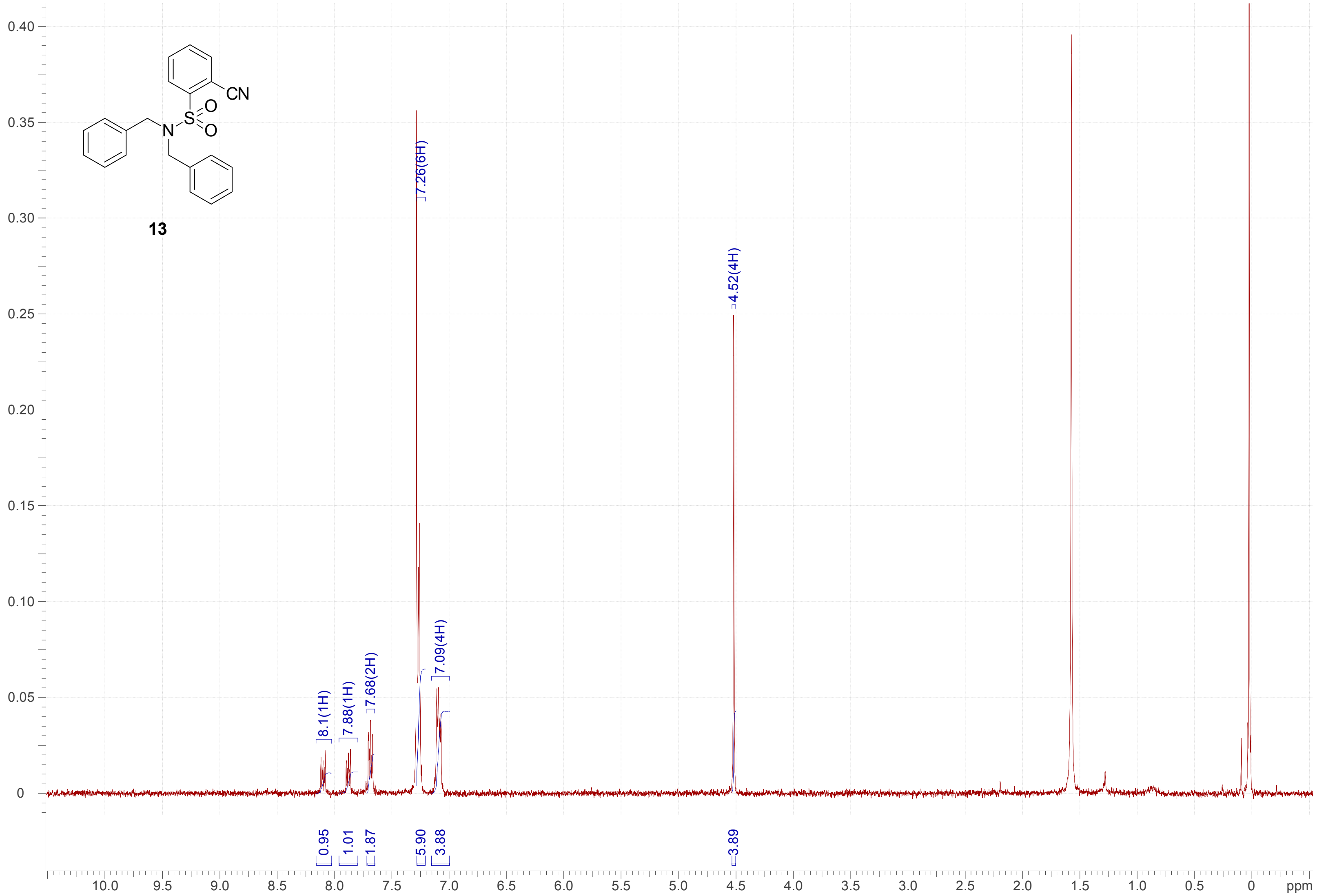

CN
S
O
O
N
13
7.26(6H)
4.52(4H)
8.1(1H)
7.88(1H)
7.68(2H)
7.09(4H)
0.95
1.01
1.87
5.90
3.88
3.89
0.40
0.35
0.30
0.25
0.20
0.15
0.10
0.05
0
10.0 9.5 9.0 8.5 8.0 7.5 7.0 6.5 6.0 5.5 5.0 4.5 4.0 3.5 3.0 2.5 2.0 1.5 1.0 0.5 0 ppm

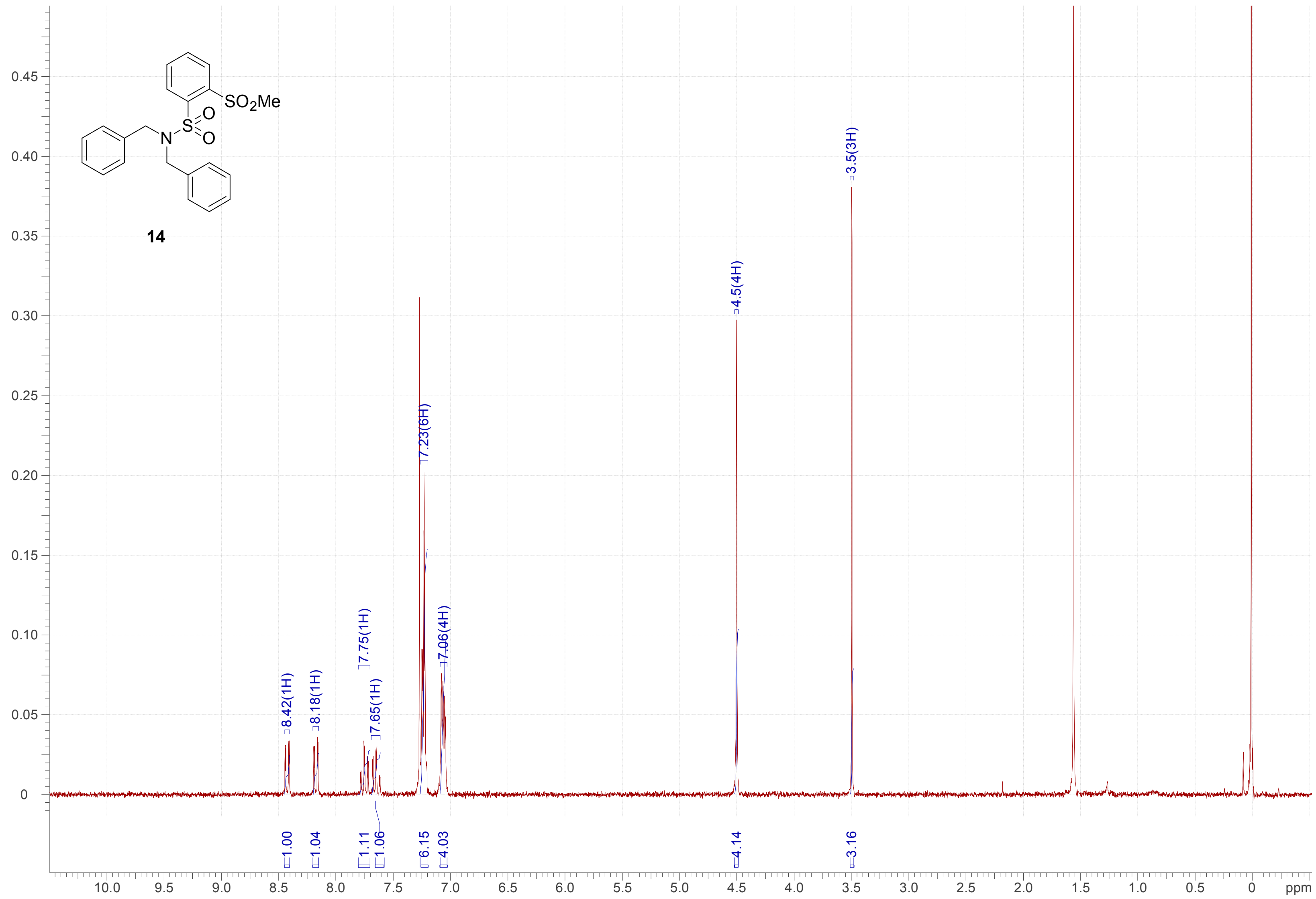

SO2Me
O
S
O
N
14
8.42(1H)
8.18(1H)
7.75(1H)
7.65(1H)
7.23(6H)
7.06(4H)
4.5(4H)
3.5(3H)
1.00
1.04
1.11
1.06
6.15
4.03
4.14
3.16
0.45
0.40
0.35
0.30
0.25
0.20
0.15
0.10
0.05
0
10.0
9.5
9.0
8.5
8.0
7.5
7.0
6.5
6.0
5.5
5.0
4.5
4.0
3.5
3.0
2.5
2.0
1.5
1.0
0.5
0
ppm

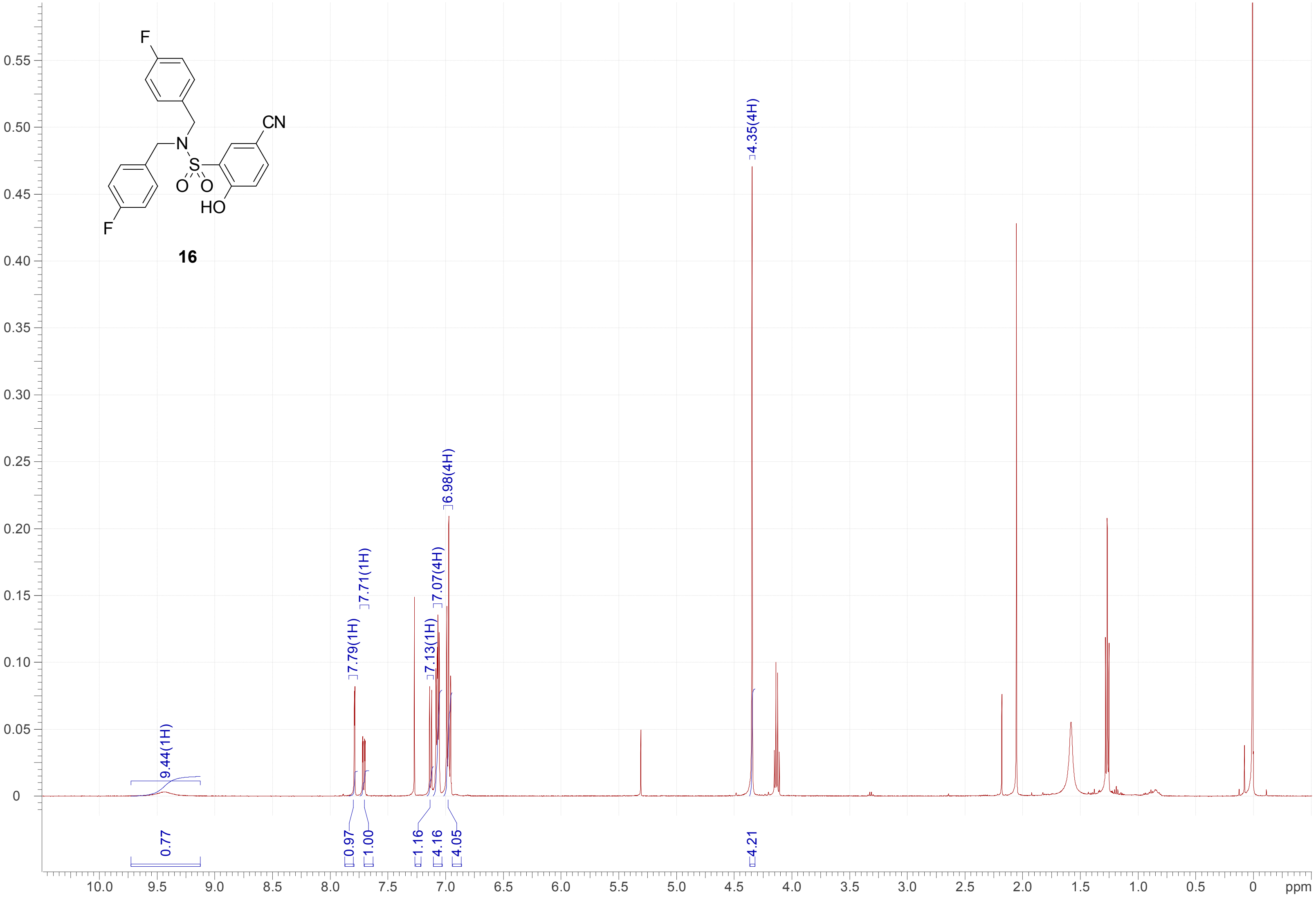

16
CN
HO
F
N
S
O
9.44(1H)
7.79(1H)
7.71(1H)
7.13(1H)
7.07(4H)
6.98(4H)
4.35(4H)
0.77
0.97
1.00
1.16
4.16
4.05
4.21
ppm

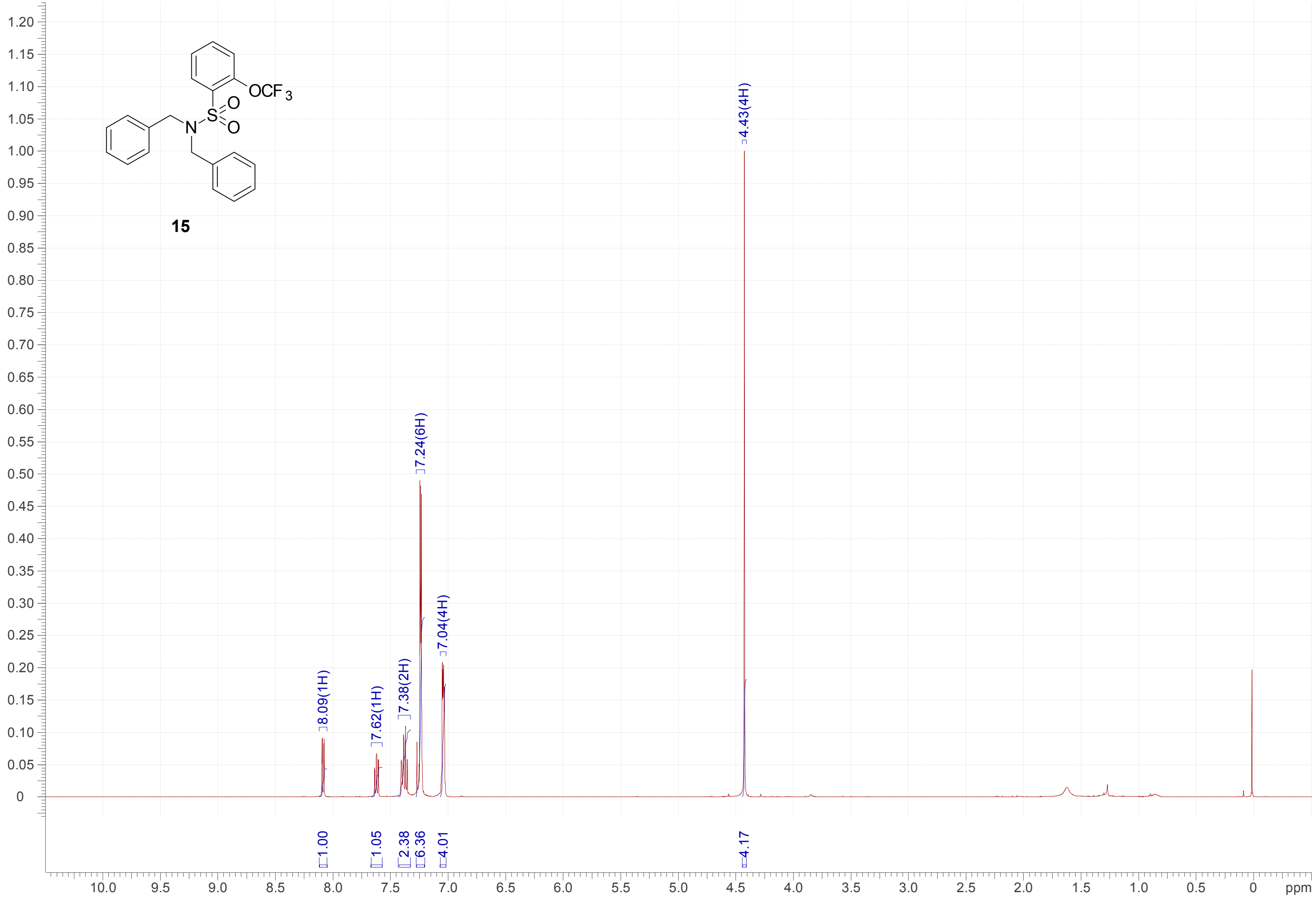

OCF3
S
O
O
N
15
8.09(1H)
7.62(1H)
7.38(2H)
7.24(6H)
7.04(4H)
4.43(4H)
1.00
1.05
2.38
6.36
4.01
4.17
ppm

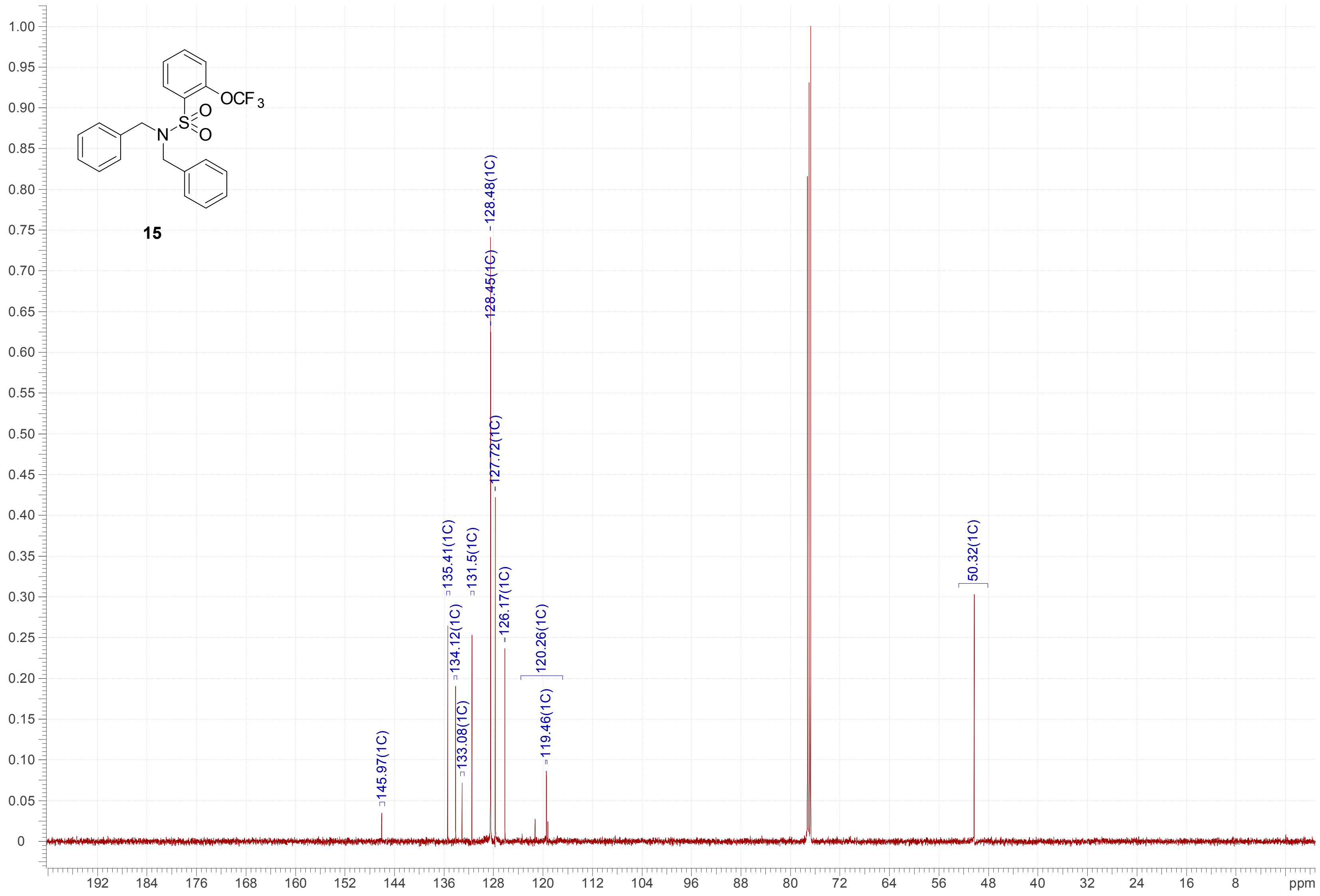

OCF3
15
145.97(1C)
135.41(1C)
134.12(1C)
133.08(1C)
131.5(1C)
128.48(1C)
128.45(1C)
127.72(1C)
126.17(1C)
120.26(1C)
119.46(1C)
50.32(1C)
ppm

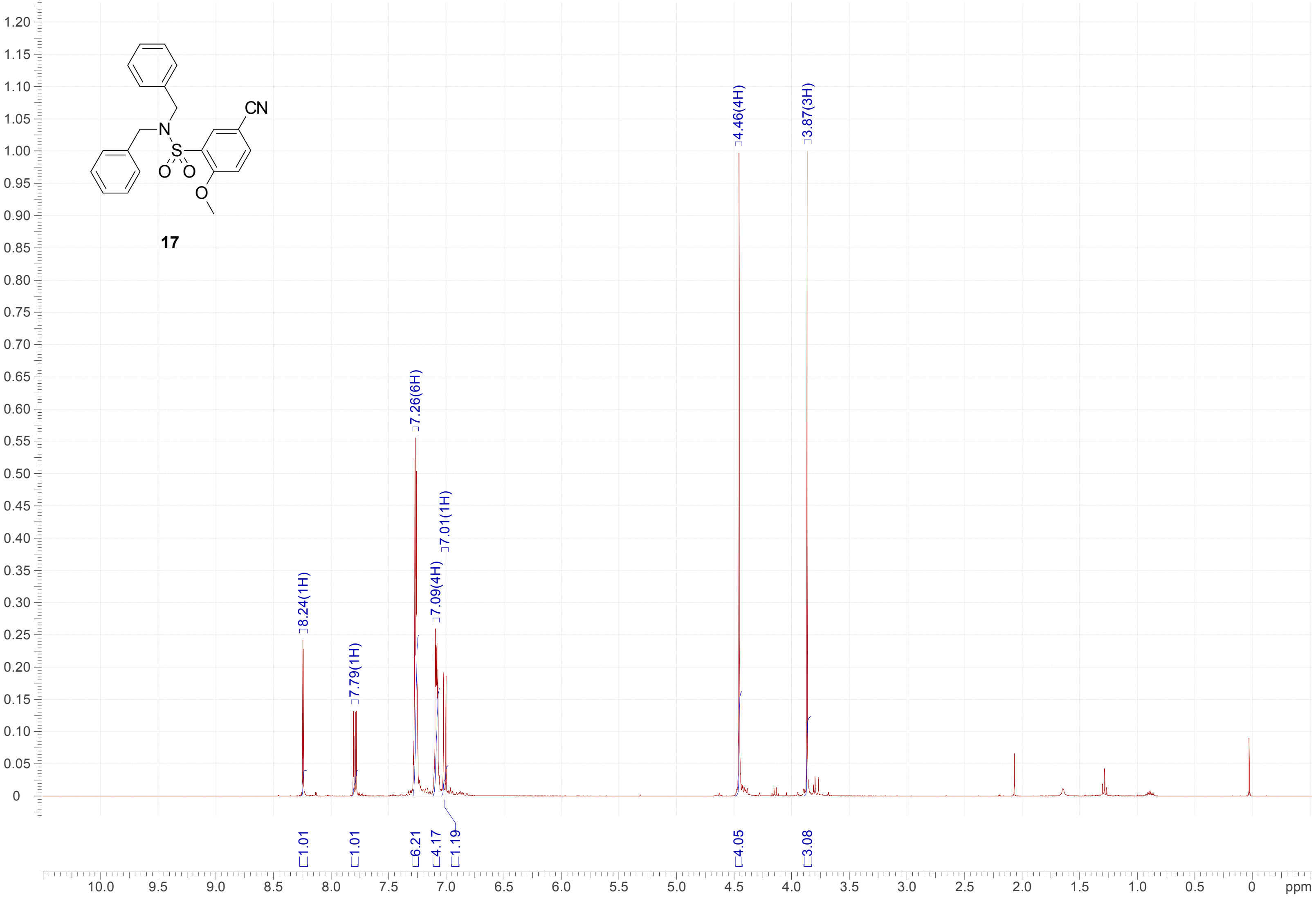

CN
N
S
O
O
O
17
8.24(1H)
7.79(1H)
7.26(6H)
7.09(4H)
7.01(1H)
4.46(4H)
3.87(3H)
1.01
1.01
6.21
4.17
1.19
4.05
3.08
ppm

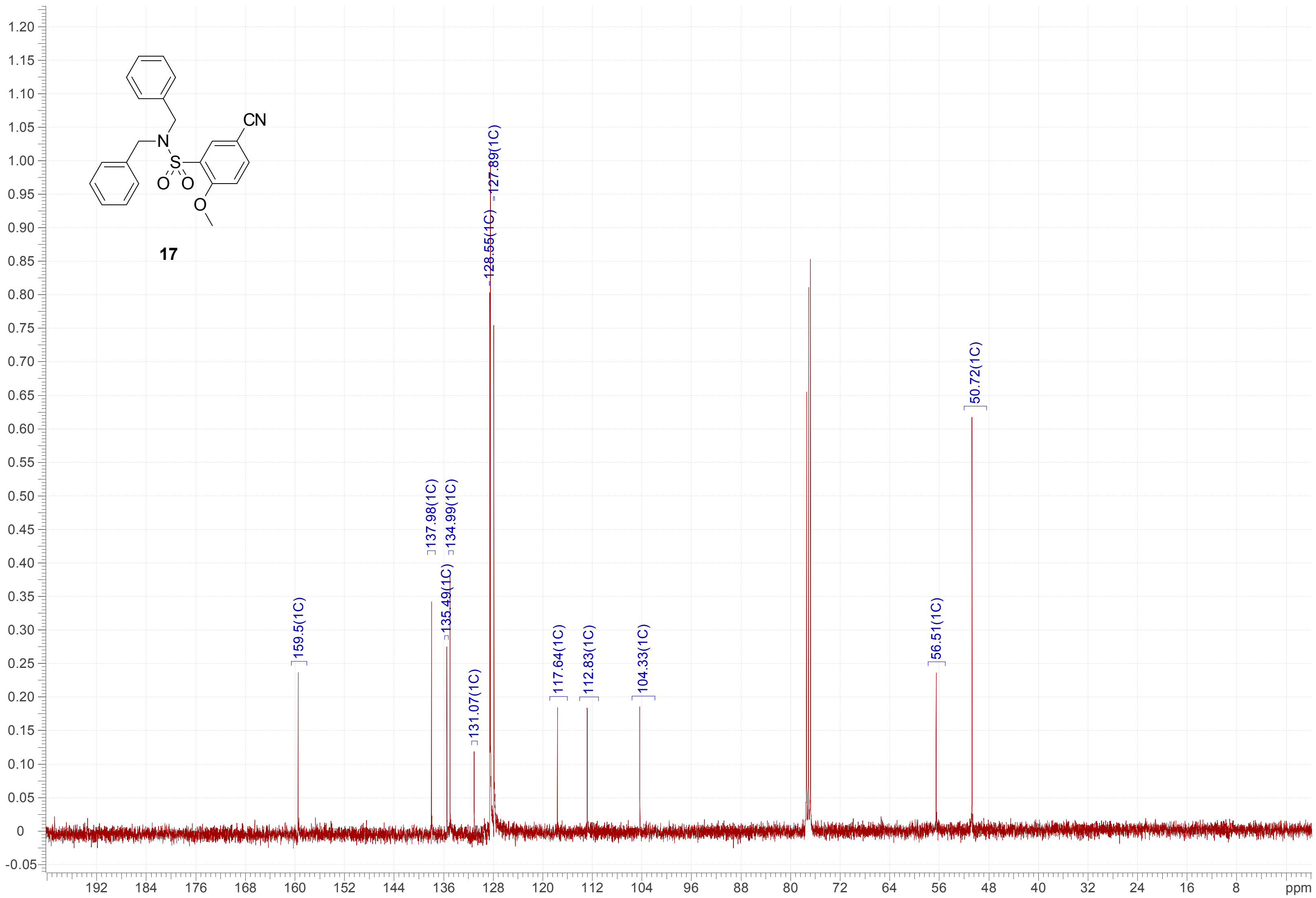
CN
N
S
O O
O
17
159.5(1C)
137.98(1C)
135.49(1C)
134.99(1C)
131.07(1C)
128.55(1C)
127.89(1C)
117.64(1C)
112.83(1C)
104.33(1C)
56.51(1C)
50.72(1C)
ppm

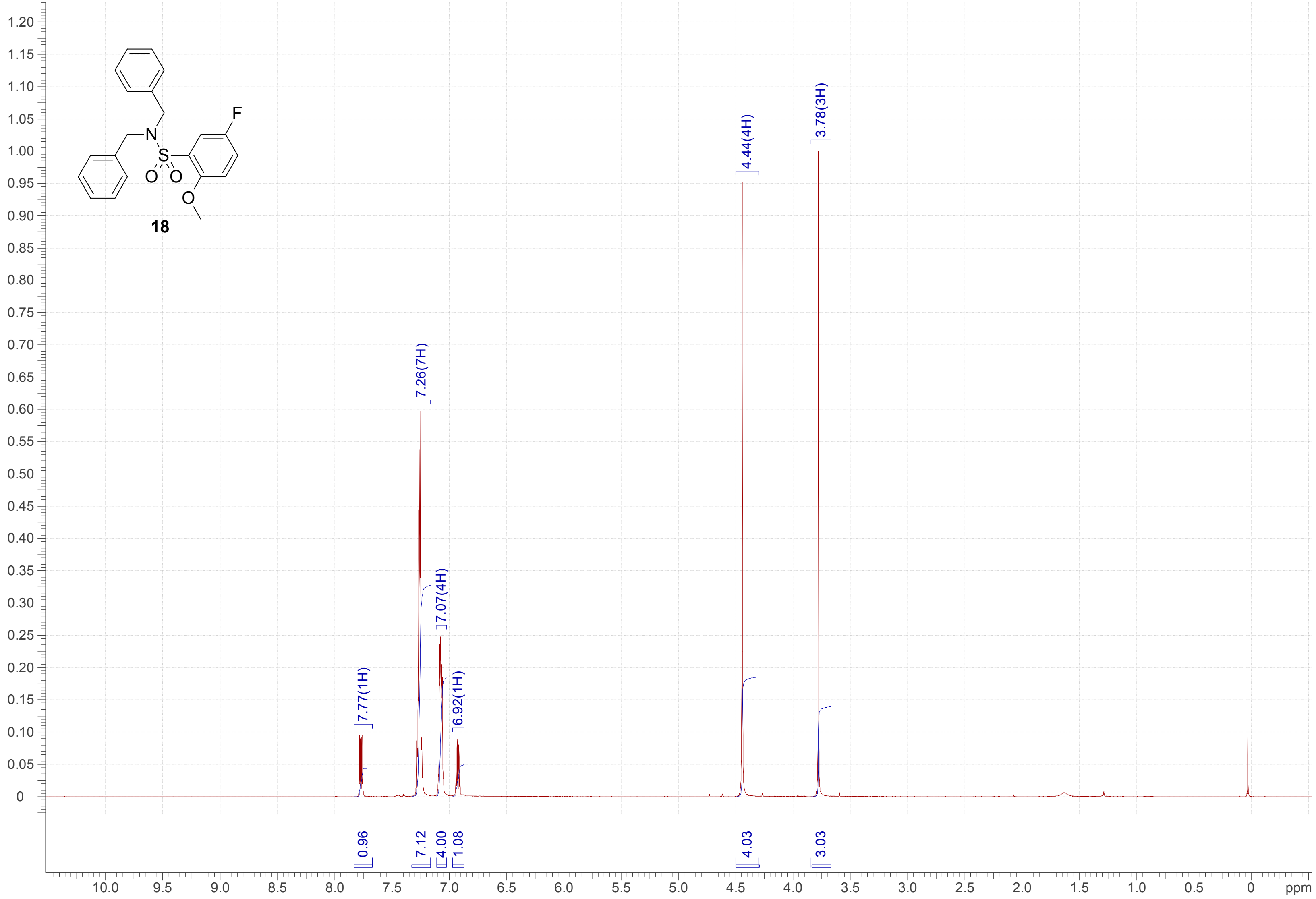
18
F
N
S
O
O
O
7.77(1H)
7.26(7H)
7.07(4H)
6.92(1H)
4.44(4H)
3.78(3H)
0.96
7.12
4.00
1.08
4.03
3.03
ppm

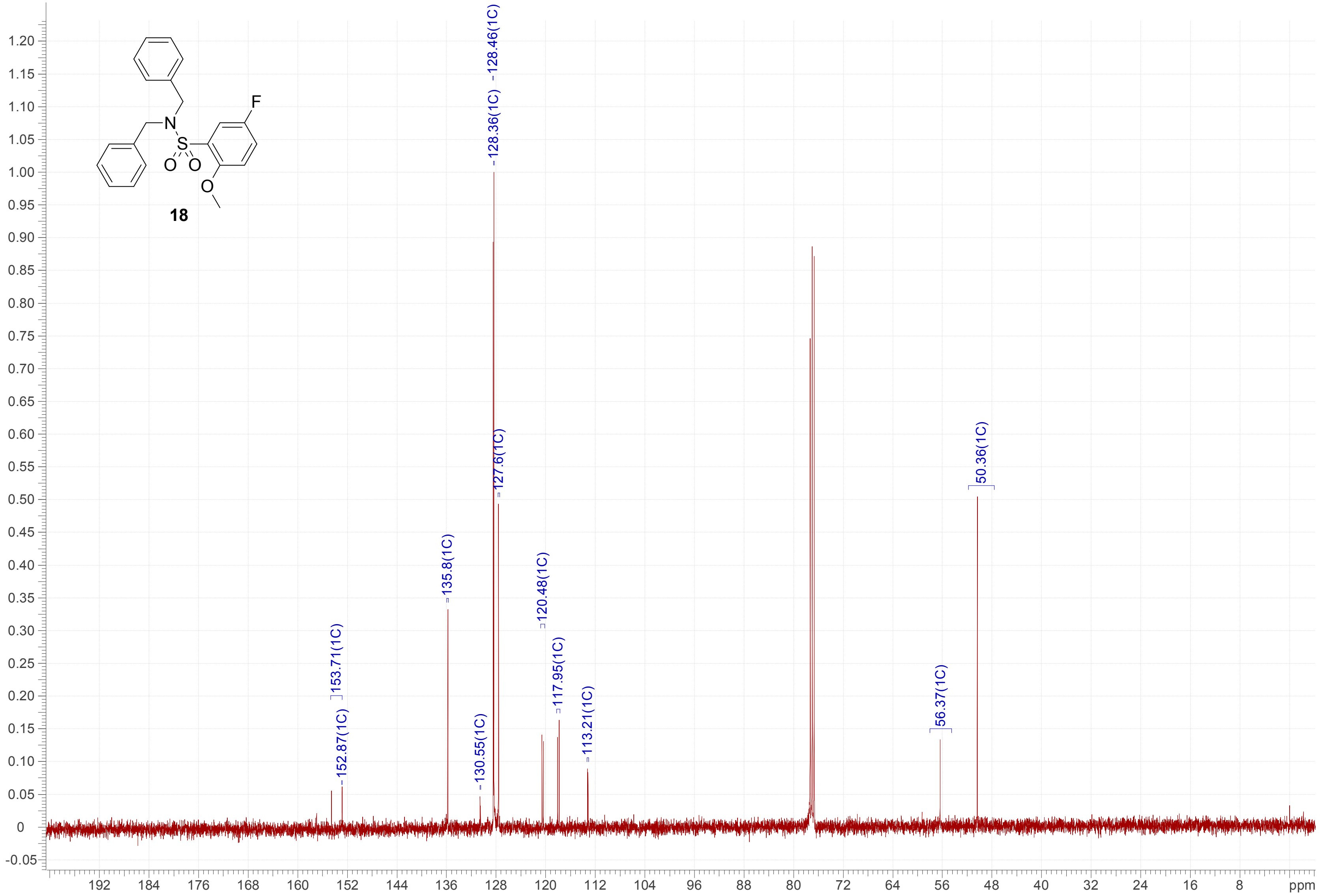

F
N
S
O O
O
18
153.71(1C)
152.87(1C)
135.8(1C)
130.55(1C)
128.46(1C)
128.36(1C)
127.6(1C)
120.48(1C)
117.95(1C)
113.21(1C)
56.37(1C)
50.36(1C)
1.20
1.15
1.10
1.05
1.00
0.95
0.90
0.85
0.80
0.75
0.70
0.65
0.60
0.55
0.50
0.45
0.40
0.35
0.30
0.25
0.20
0.15
0.10
0.05
0
-0.05
192 184 176 168 160 152 144 136 128 120 112 104 96 88 80 72 64 56 48 40 32 24 16 8 ppm

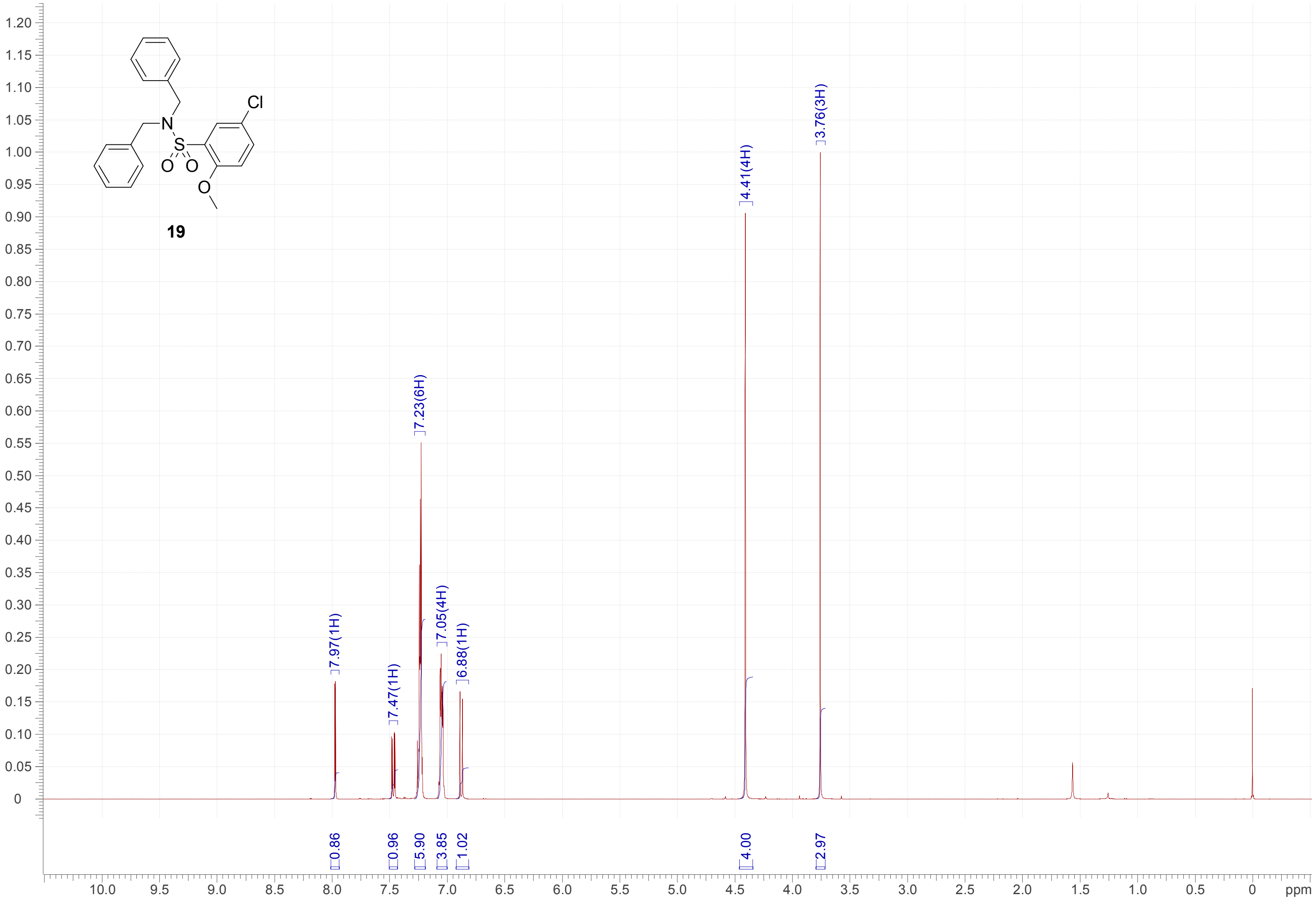

Cl
N
S
O
O
O
19
7.97(1H)
7.47(1H)
7.23(6H)
7.05(4H)
6.88(1H)
4.41(4H)
3.76(3H)
0.86
0.96
5.90
3.85
1.02
4.00
2.97
ppm

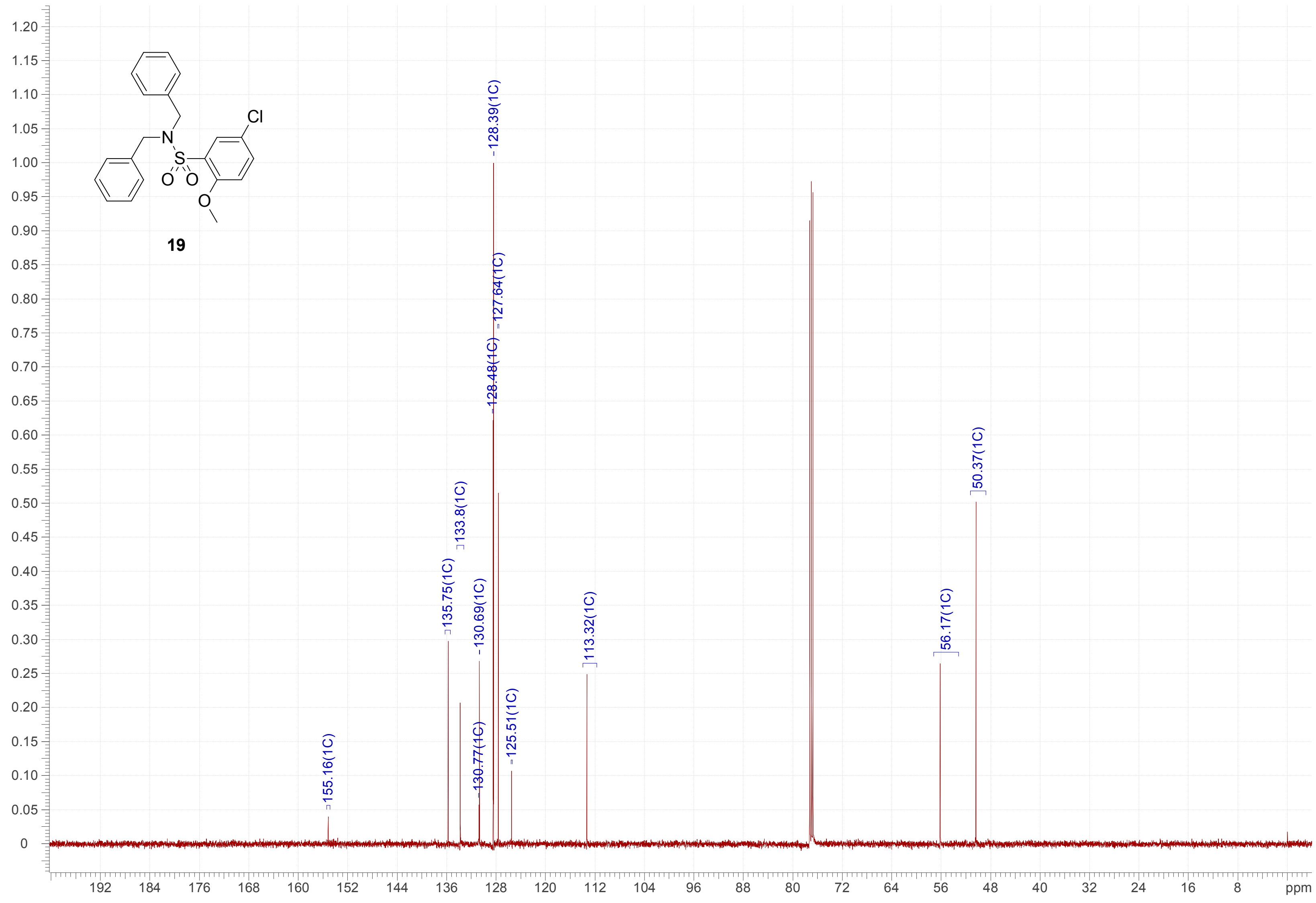

Cl
N
S
O
O
O
19
155.16(1C)
135.75(1C)
133.8(1C)
130.77(1C)
130.69(1C)
128.48(1C)
128.39(1C)
127.64(1C)
125.51(1C)
113.32(1C)
56.17(1C)
50.37(1C)
ppm

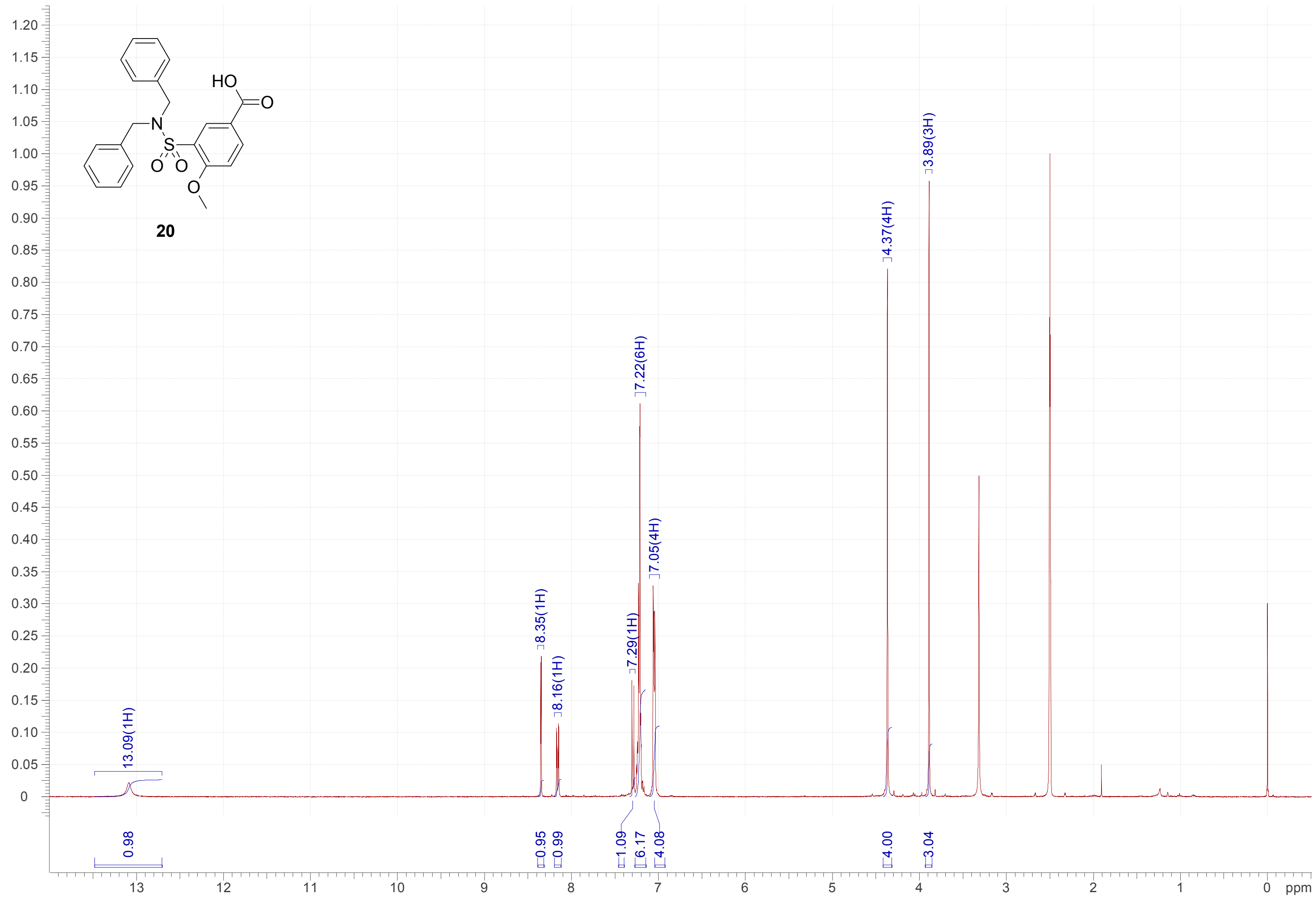
HO
O
N
S
O
O
O
20
13.09(1H)
8.35(1H)
8.16(1H)
7.29(1H)
7.22(6H)
7.05(4H)
4.37(4H)
3.89(3H)
0.98
0.95
0.99
1.09
6.17
4.08
4.00
3.04
ppm

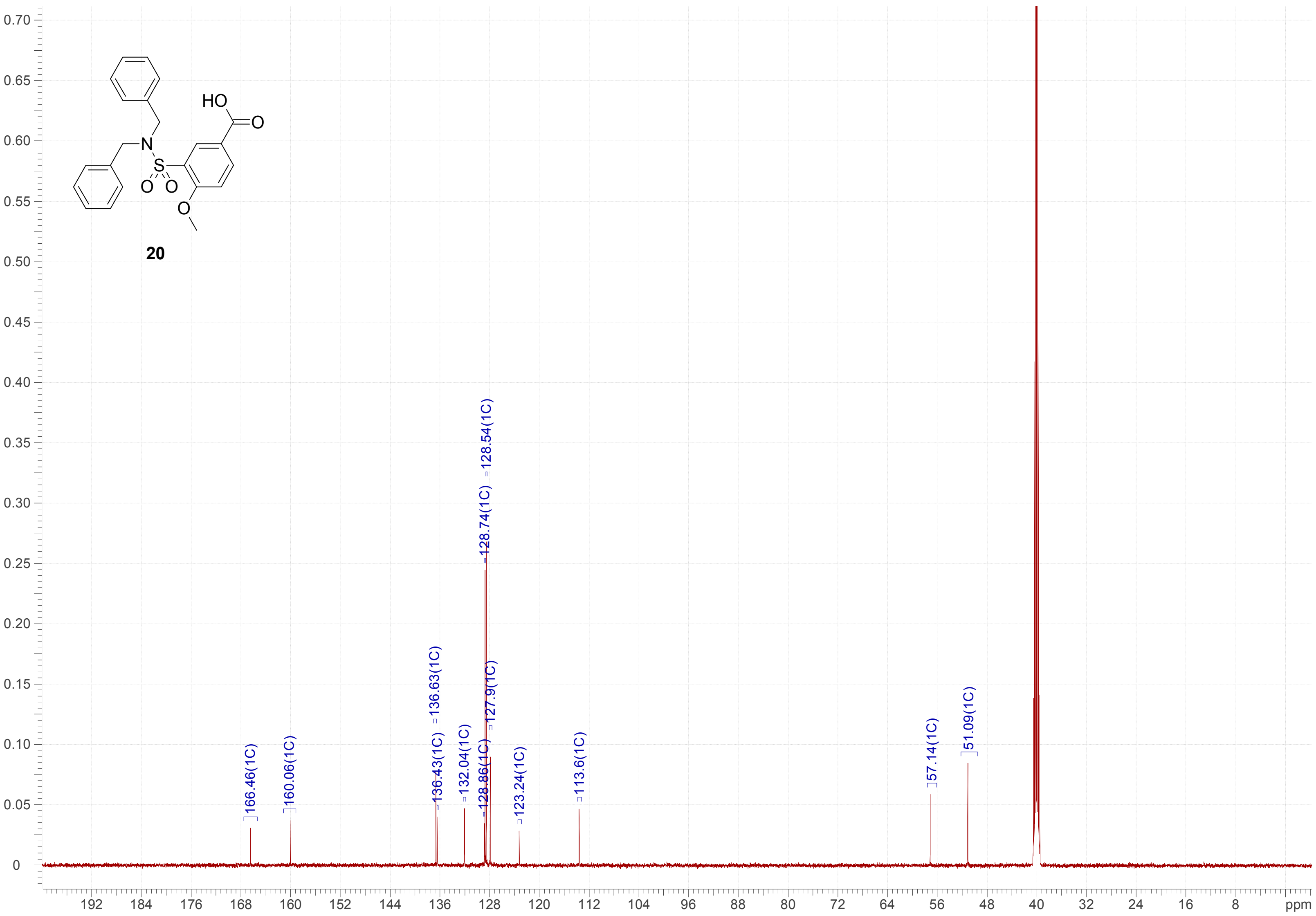
20
166.46(1C)
160.06(1C)
136.63(1C)
136.43(1C)
132.04(1C)
128.86(1C)
128.74(1C)
128.54(1C)
127.9(1C)
123.24(1C)
113.6(1C)
57.14(1C)
51.09(1C)
ppm

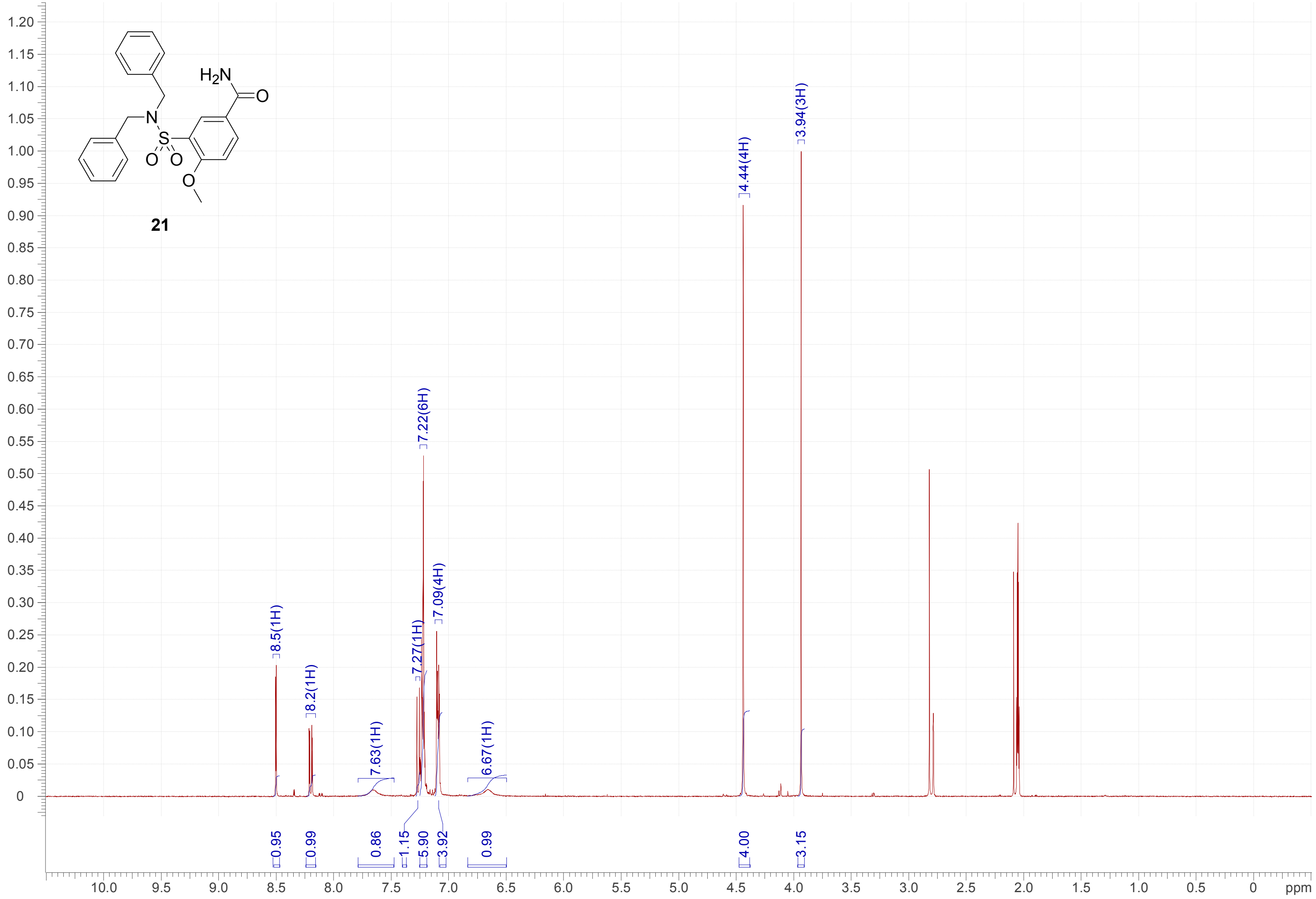

H2N
O
N
S
O
O
O
21
8.5(1H)
8.2(1H)
7.63(1H)
7.27(1H)
7.22(6H)
7.09(4H)
6.67(1H)
4.44(4H)
3.94(3H)
0.95
0.99
0.86
1.15
5.90
3.92
0.99
4.00
3.15
ppm

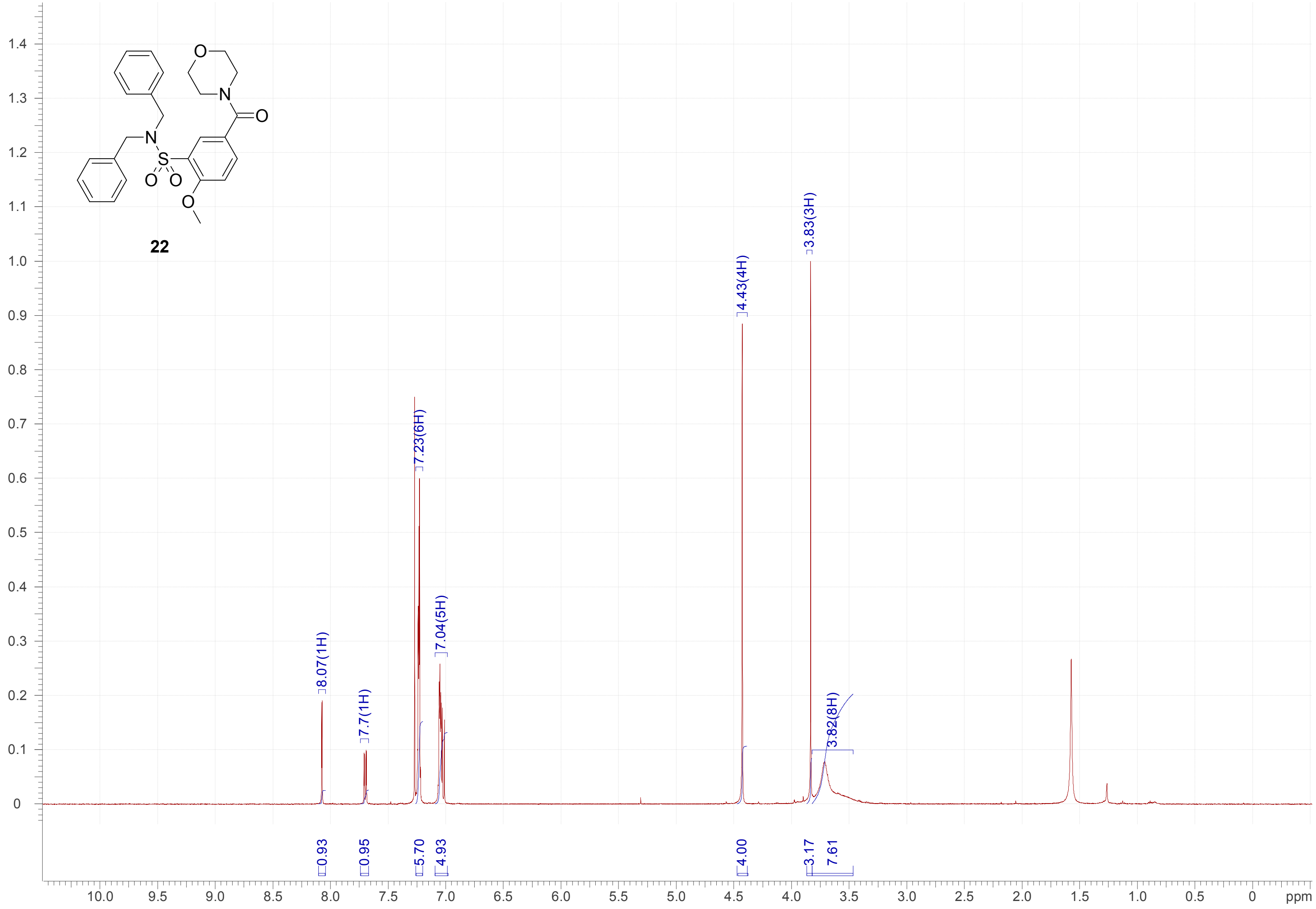
22
8.07(1H)
7.7(1H)
7.23(6H)
7.04(5H)
4.43(4H)
3.83(3H)
3.82(8H)
0.93
0.95
5.70
4.93
4.00
3.17
7.61
ppm

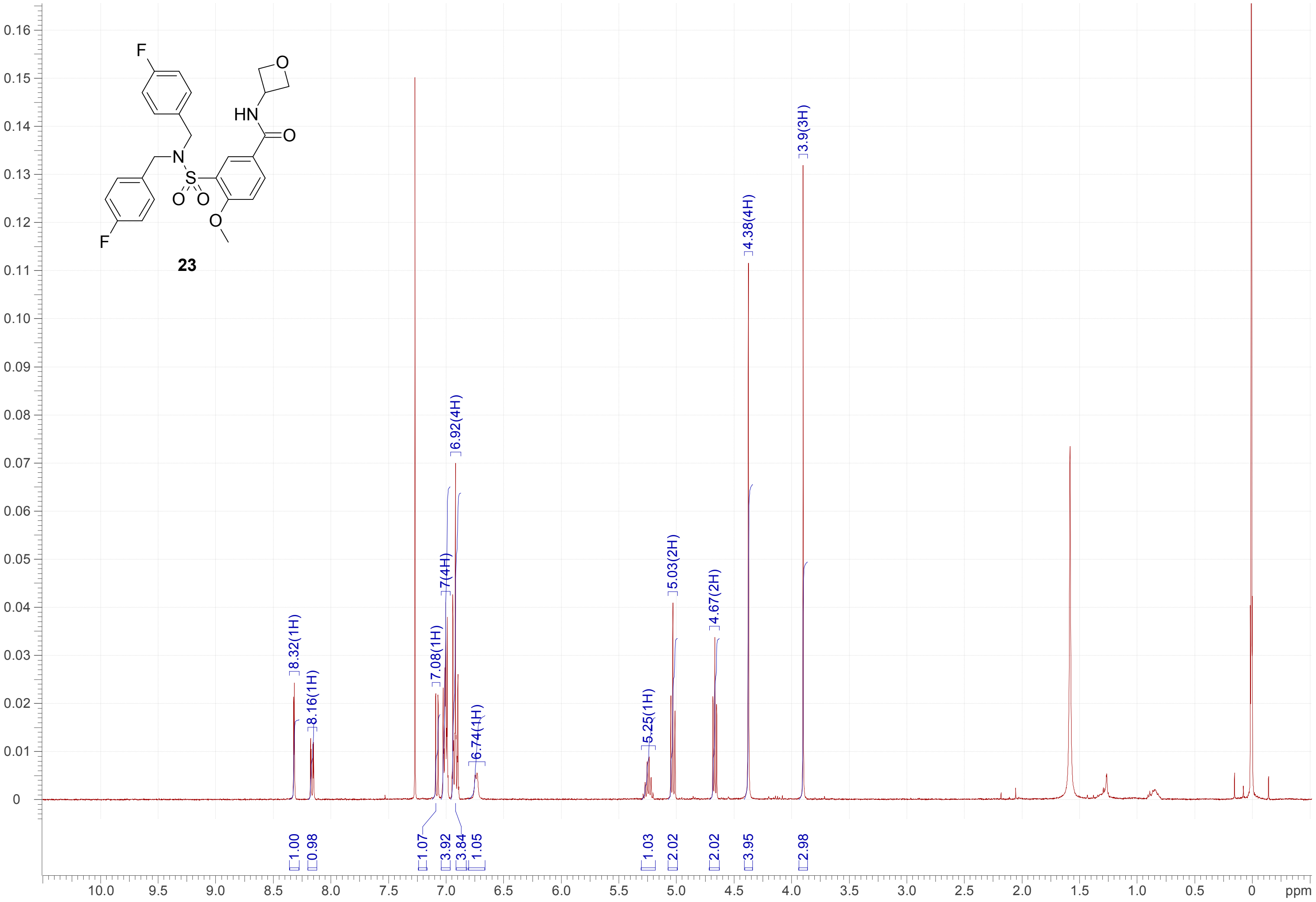

23
F
F
O
HN
O
N
S
O O
O
8.32(1H)
8.16(1H)
7.08(1H)
7(4H)
6.92(4H)
6.74(1H)
5.25(1H)
5.03(2H)
4.67(2H)
4.38(4H)
3.9(3H)
1.00
0.98
1.07
3.92
3.84
1.05
1.03
2.02
2.02
3.95
2.98
ppm

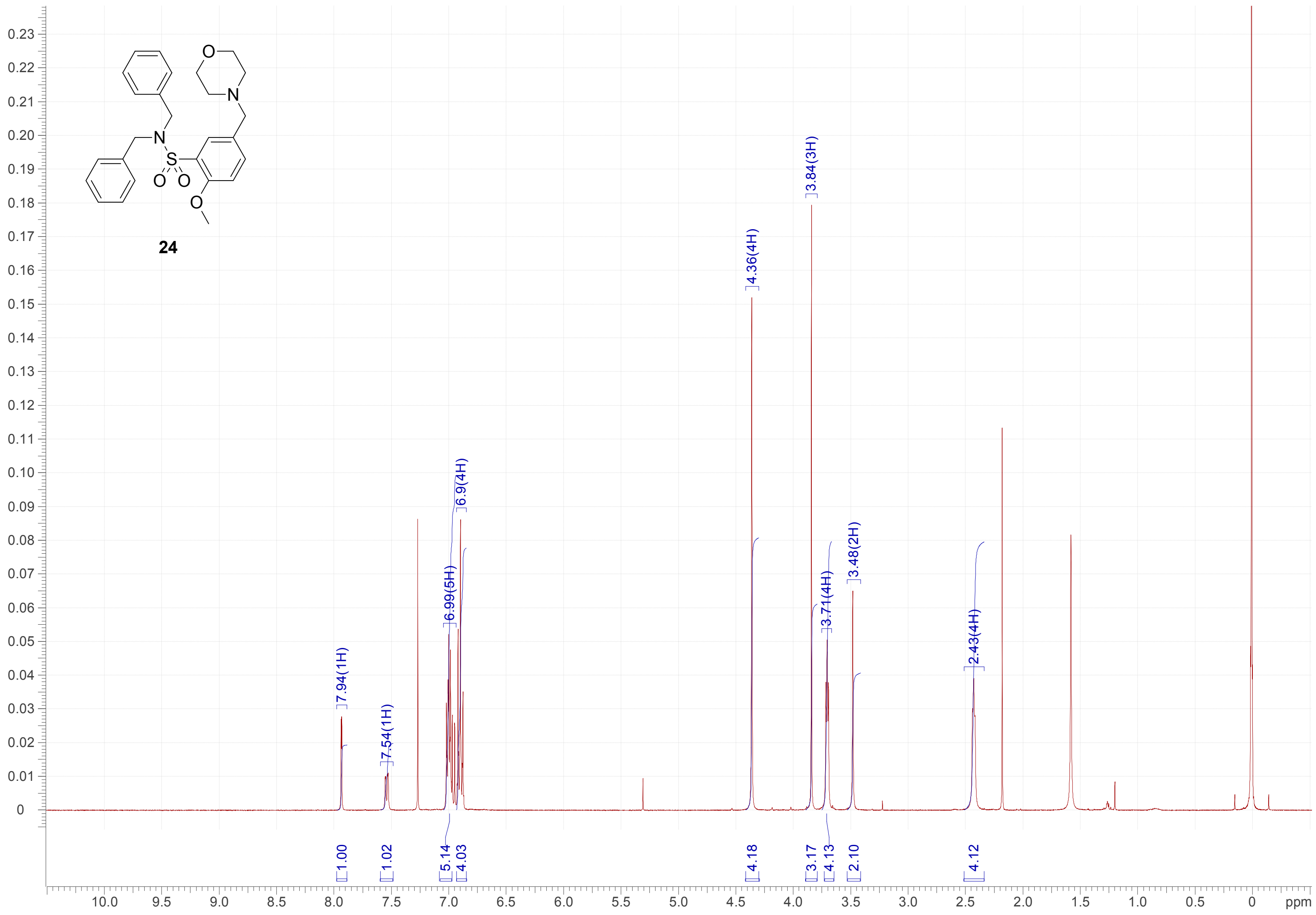
24
7.94(1H)
7.54(1H)
6.99(5H)
6.9(4H)
4.36(4H)
3.84(3H)
3.71(4H)
3.48(2H)
2.43(4H)
1.00
1.02
5.14
4.03
4.18
3.17
4.13
2.10
4.12
ppm

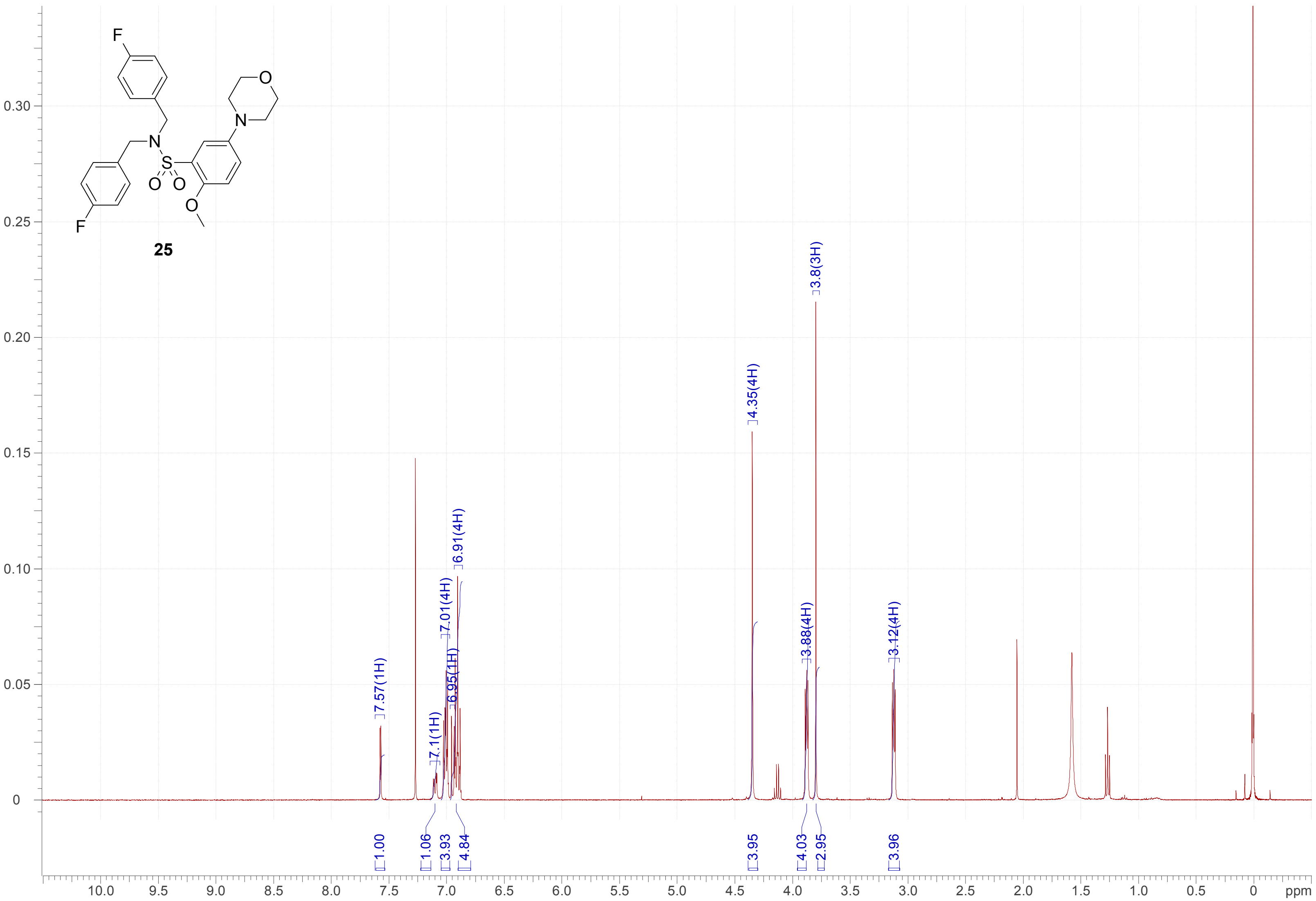

25
7.57(1H)
7.1(1H)
7.01(4H)
6.95(1H)
6.91(4H)
4.35(4H)
3.88(4H)
3.8(3H)
3.12(4H)
1.00
1.06
3.93
4.84
3.95
4.03
2.95
3.96
ppm

DESSOY MAD1834_T6 CDCl3 250MHz nov27madH1

Acquisition Time (sec)	3.2768	***Comment***	DESSOY MAD1834_T6 CDCl3 250MHz nov27madH1			***Date***	27 Nov 2018 09:23:04
File Name	\\nmrsparc.iqm.unicamp.br\espectros\bruker250\2018\nov18\Reserva\Luiz Carlos\nov27madH1_001001r					***Frequency (MHz)***	250.13
Nucleus	1H	***Number of Transients***	8	***Original Points Count***	16384	***Points Count***	65536
Pulse Sequence	zg30	***Solvent***	CHLOROFORM-D	***Sweep Width (Hz)***	5000.00	***Temperature (degree C)***	25.148

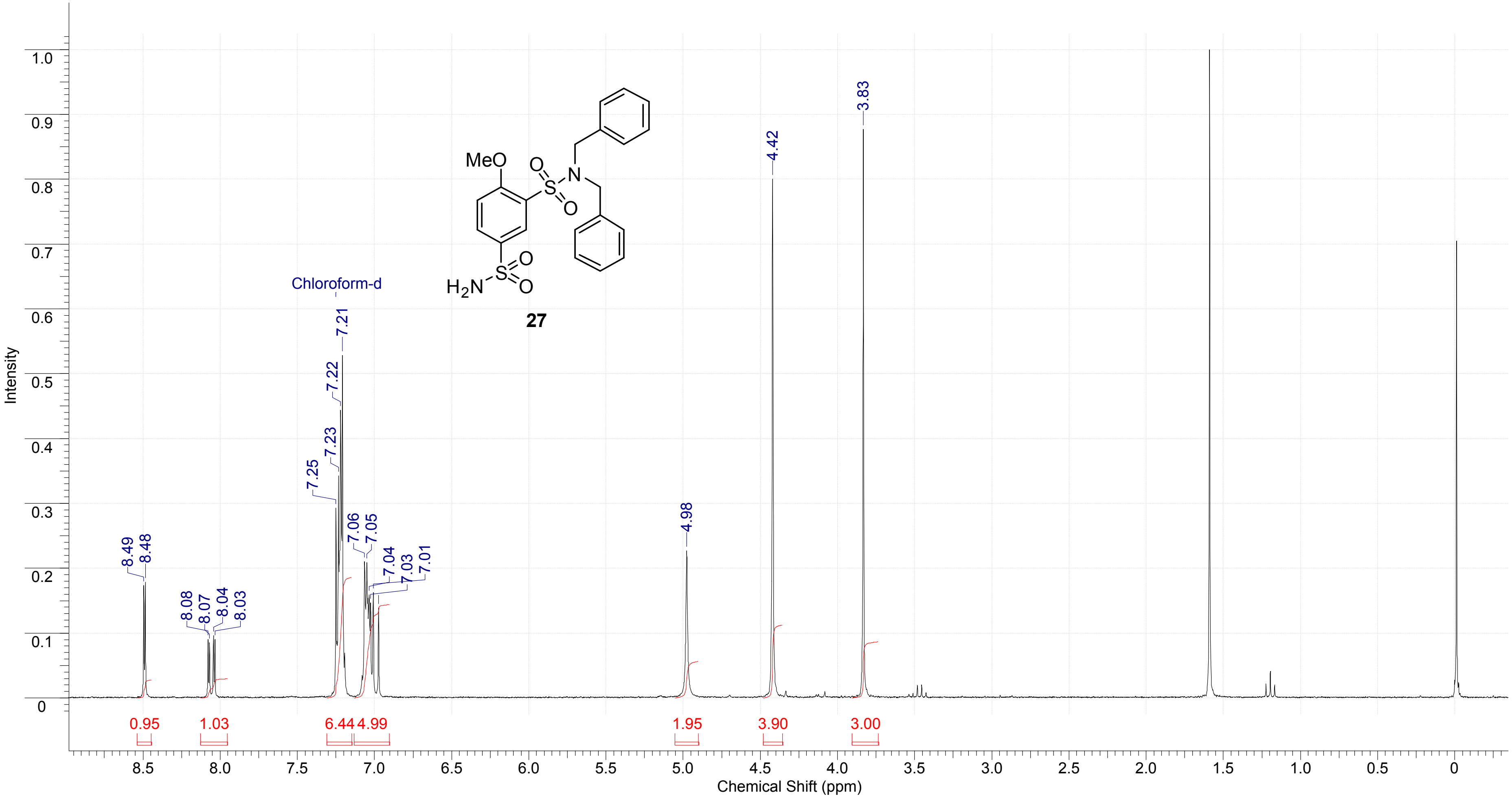

Dessoy - MAD1834 - CDCl3 - AV 500 MHz - nov22madH1 13C

Acquisition Time (sec)	0.4981	***Comment***	Dessoy - MAD1834 - CDCl3 - AV 500 MHz - nov22madH1 13C			***Date***	22 Nov 2018 12:42:56
File Name	\\nmrsparc.iqm.unicamp.br\espectros\avance500\2018\nov18\Sala\Luiz Carlos\nov22madH1_002001r					***Frequency (MHz)***	125.69
Nucleus	13C	***Number of Transients***	256	***Original Points Count***	16384	***Points Count***	32768
Pulse Sequence	zgpg30	***Solvent***	CHLOROFORM-D	***Sweep Width (Hz)***	32894.74	***Temperature (degree C)***	25.151

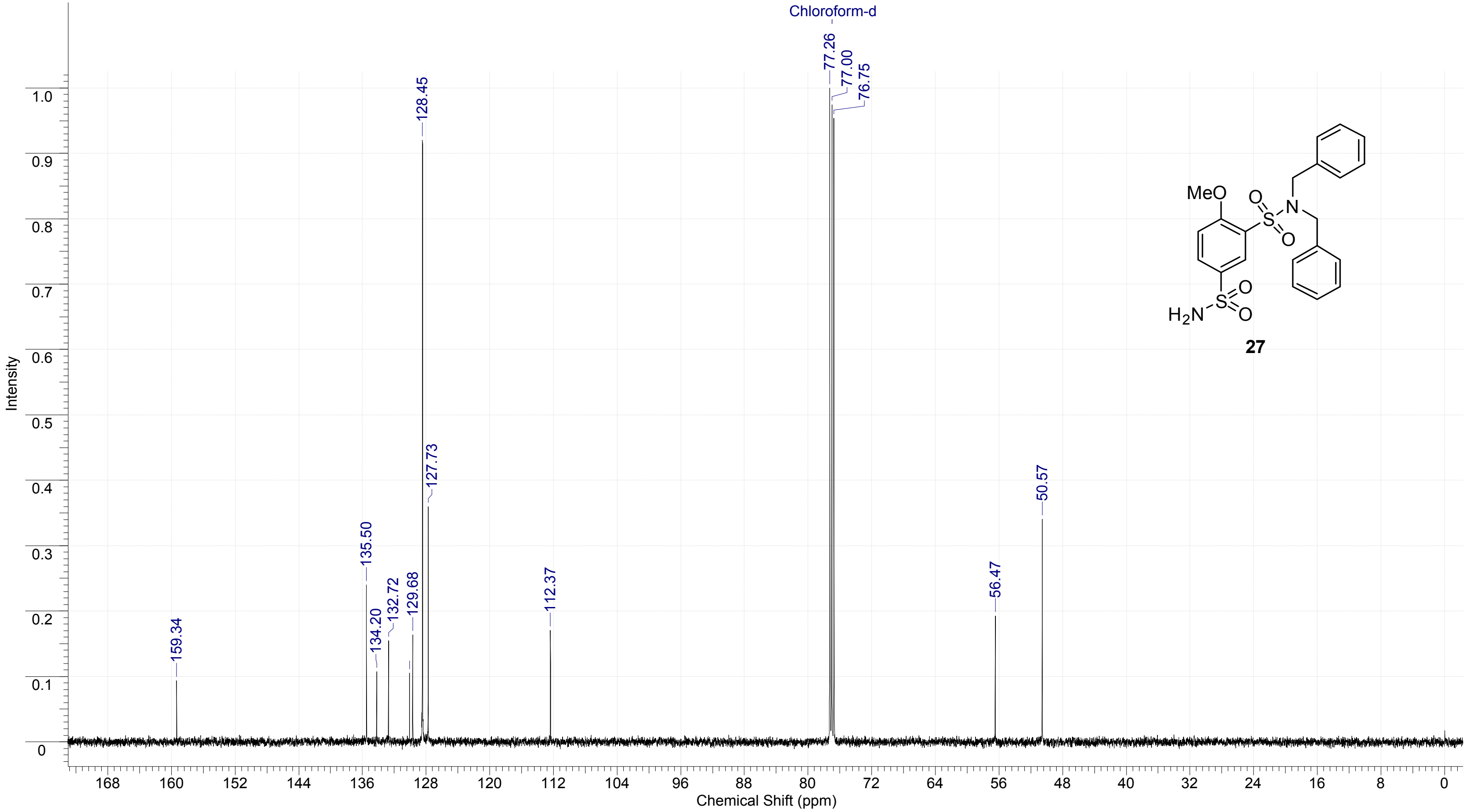

Dessoy - MAD 1883 - CDCl3 - Avance 500 MHz - abr05madH1 - 1H

Acquisition Time (sec)	1.5903	***Comment***	Dessoy - MAD 1883 - CDCl3 - Avance 500 MHz - abr05madH1 - 1H			***Date***	05 Apr 2019 07:33:42
File Name	\\nmrsparc.iqm.unicamp.br\espectros\avance500\2019\abr19\Sala\Luiz Carlos\abr05madH1_001001r					***Frequency (MHz)***	499.87
Nucleus	1H	***Number of Transients***	16	***Original Points Count***	16384	***Points Count***	65536
Pulse Sequence	zg30	***Solvent***	CHLOROFORM-D	***Sweep Width (Hz)***	10302.20	***Temperature (degree C)***	25.149

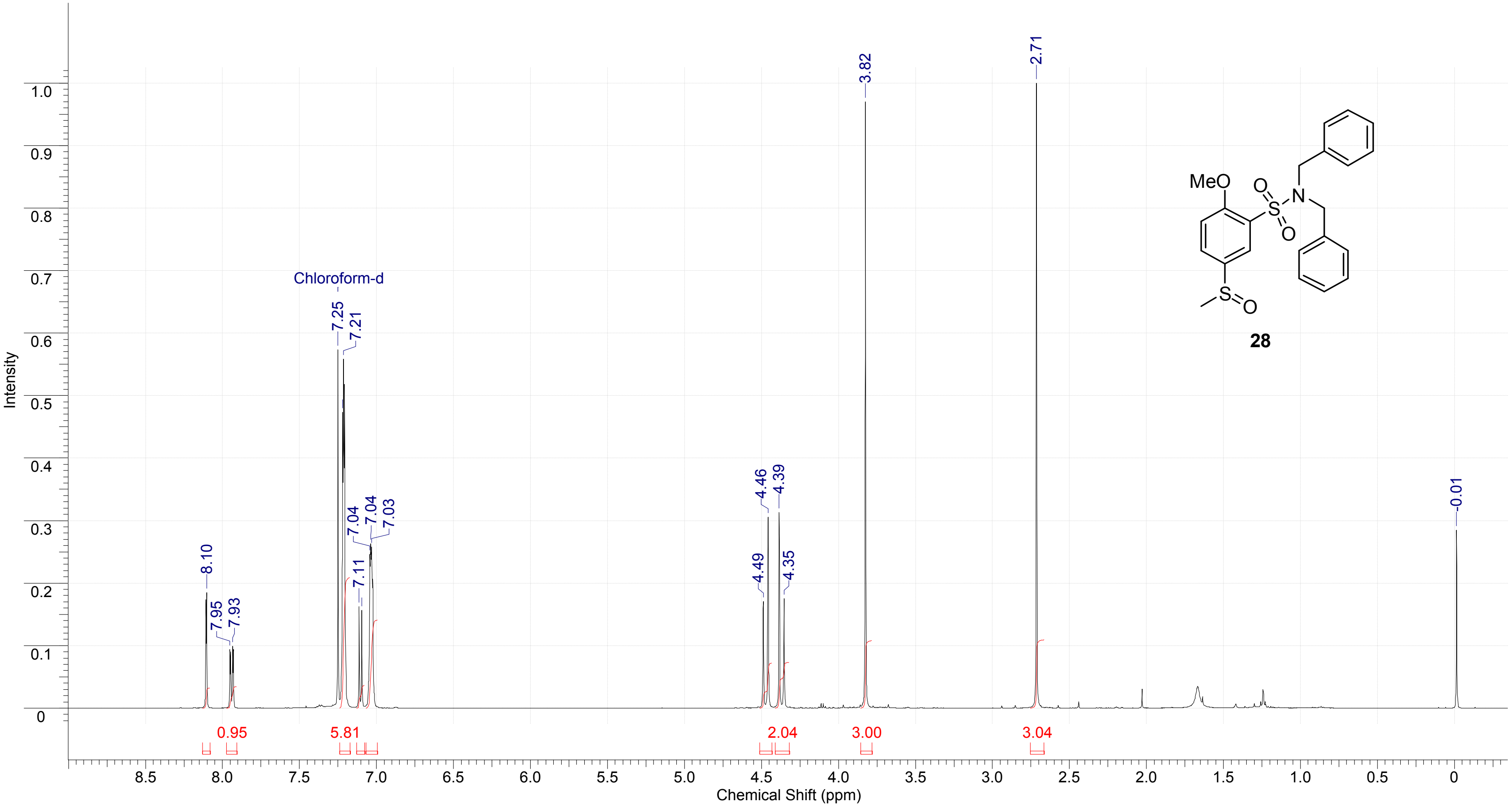

**Acquisition Time (sec)**	0.4981	**Comment**	Dessoy - MAD 1883 - CDCl3 - Avance 500 MHz - abr05madH1 - 13C			**Date**	05 Apr 2019 07:56:38
**File Name**	\\nmrsparc.iqm.unicamp.br\espectros\avance500\2019\abr19\Sala\Luiz Carlos\abr05madH1_002001r					**Frequency (MHz)**	125.69
**Nucleus**	13C	**Number of Transients**	512	**Original Points Count**	16384	**Points Count**	32768
**Pulse Sequence**	zgpg30	**Solvent**	CHLOROFORM-D	**Sweep Width (Hz)**	32894.74	**Temperature (degree C)**	25.149

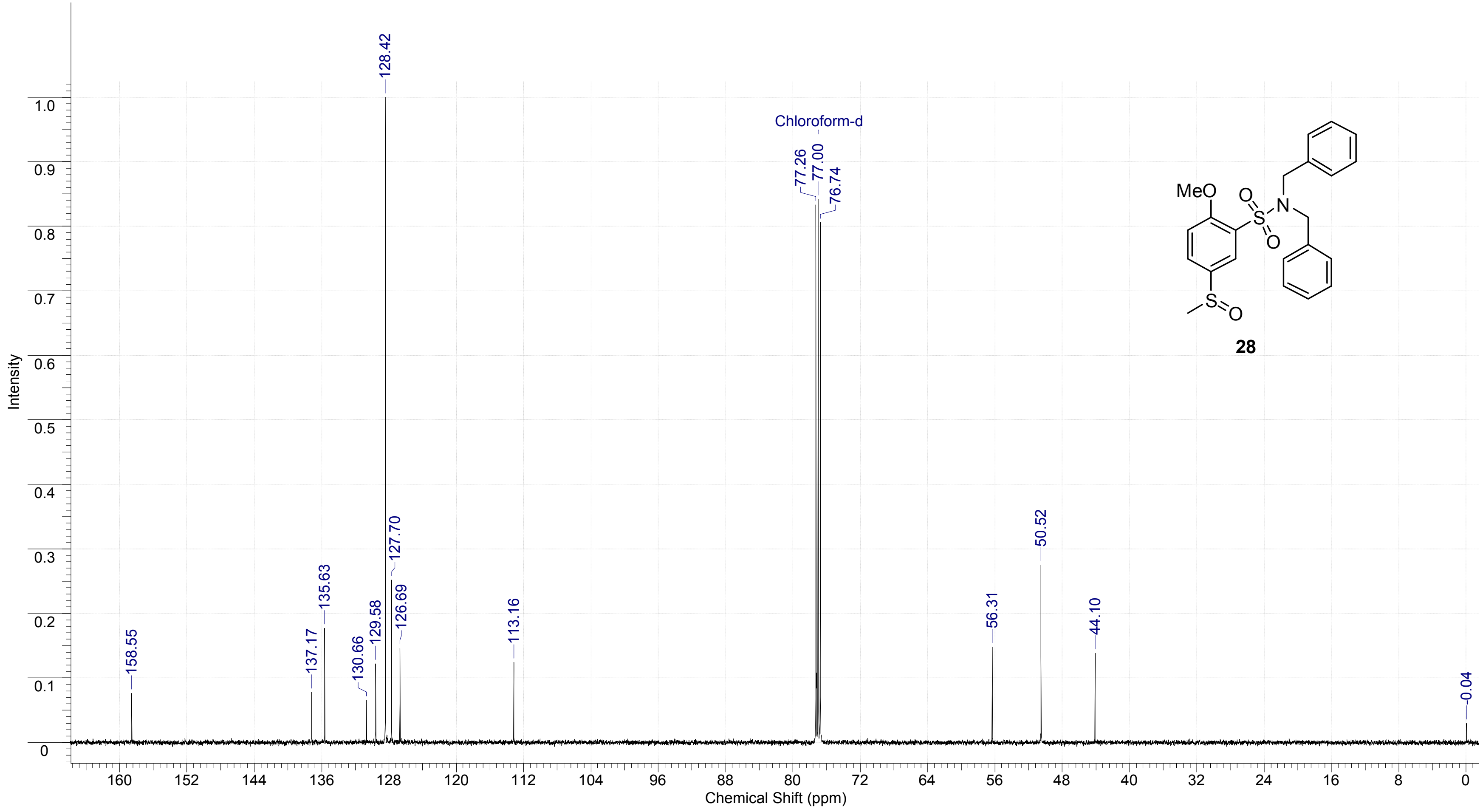

DESSOY MAD1855 CDCl3 250MHz jan30madH1

Acquisition Time (sec)	3.2768	***Comment***	DESSOY MAD1855 CDCl3 250MHz jan30madH1			***Date***	30 Jan 2019 08:12:06
File Name	\\nmrsparc.iqm.unicamp.br\espectros\bruker250\2019\jan19\Reserva\Luiz Carlos\jan30madH1_001001r					***Frequency (MHz)***	250.13
Nucleus	1H	***Number of Transients***	8	***Original Points Count***	16384	***Points Count***	65536
Pulse Sequence	zg30	***Solvent***	CHLOROFORM-D	***Sweep Width (Hz)***	5000.00	***Temperature (degree C)***	25.150

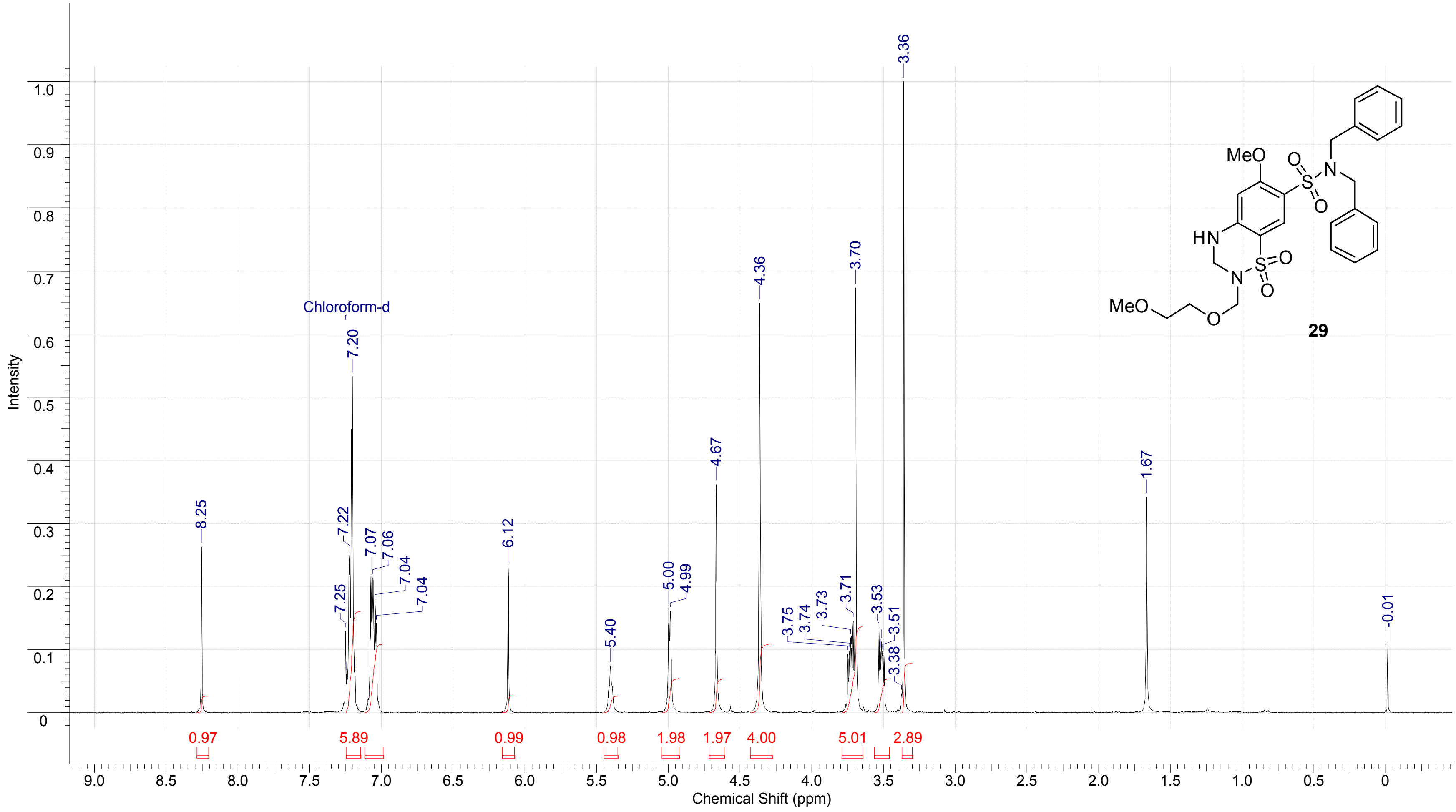

Dessoy - MAD 1855 - CDCl3 - Avance 500 MHz - jan30madC1 - 13C

Acquisition Time (sec)	0.4981	***Comment***	Dessoy - MAD 1855 - CDCl3 - Avance 500 MHz - jan30madC1 - 13C			***Date***	30 Jan 2019 10:04:44
File Name	\\nmrsparc.iqm.unicamp.br\espectros\avance500\2019\jan19\Sala\Luiz Carlos\jan30madC1_001001r					***Frequency (MHz)***	125.69
Nucleus	13C	***Number of Transients***	512	***Original Points Count***	16384	***Points Count***	32768
Pulse Sequence	zgpg30	***Solvent***	CHLOROFORM-D	***Sweep Width (Hz)***	32894.74	***Temperature (degree C)***	25.152

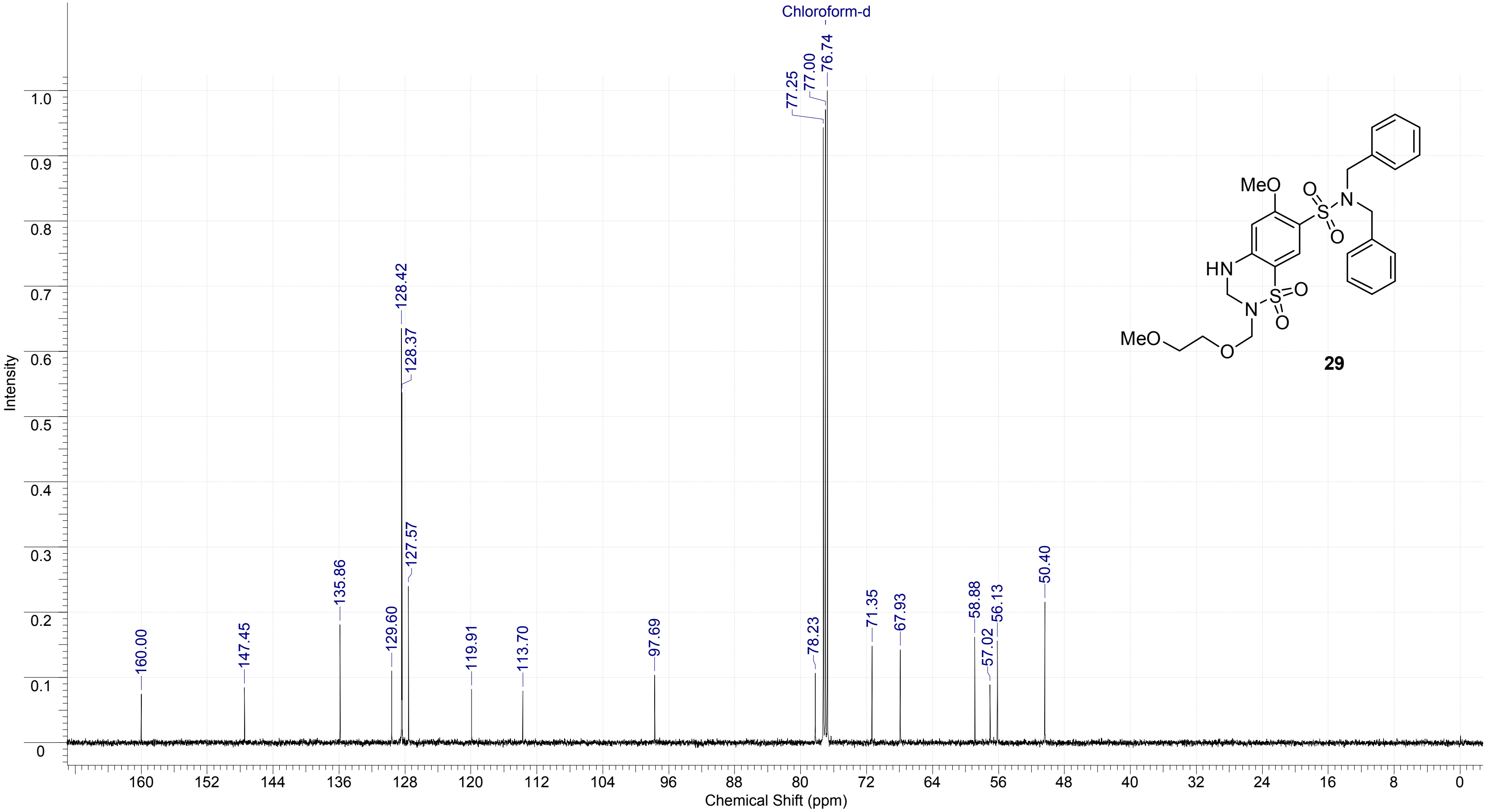

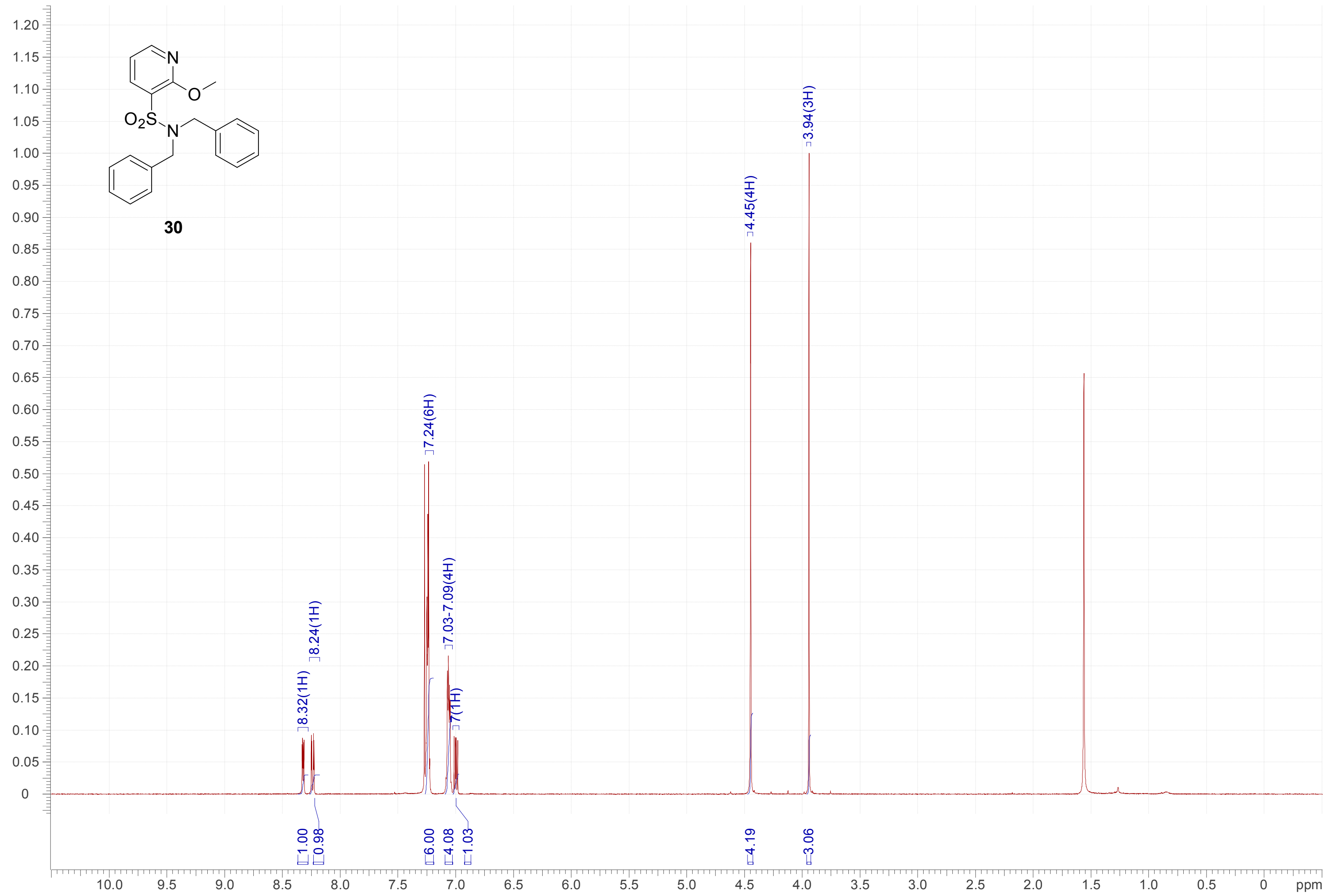

N
O
O_2S
N
30
8.32(1H)
8.24(1H)
7.24(6H)
7.03-7.09(4H)
7(1H)
4.45(4H)
3.94(3H)
1.00
0.98
6.00
4.08
1.03
4.19
3.06
ppm

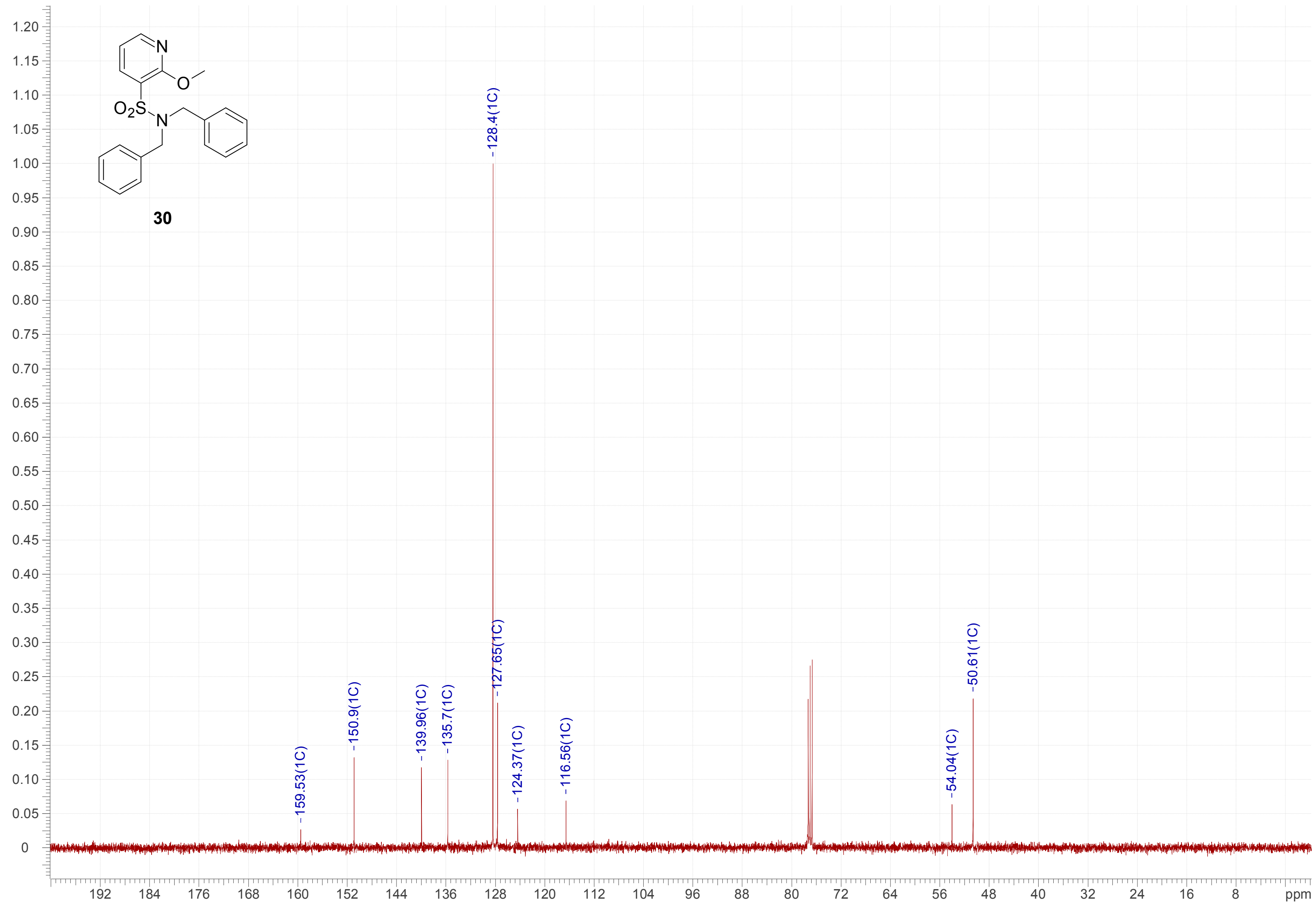
N
O
O2S
N
30
159.53(1C)
150.9(1C)
139.96(1C)
135.7(1C)
128.4(1C)
127.65(1C)
124.37(1C)
116.56(1C)
54.04(1C)
50.61(1C)
ppm

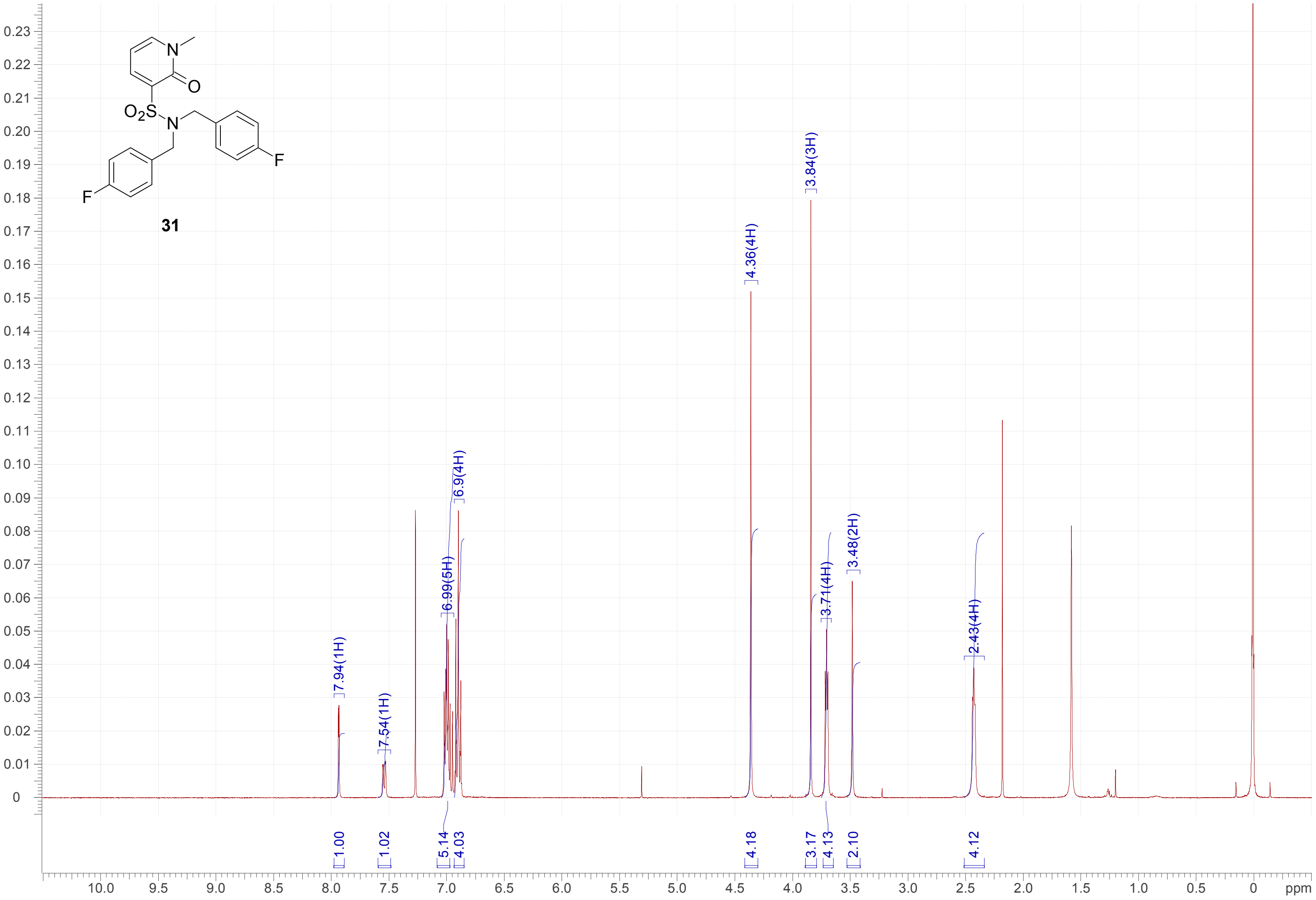
31
7.94(1H)
7.54(1H)
6.99(5H)
6.9(4H)
4.36(4H)
3.84(3H)
3.71(4H)
3.48(2H)
2.43(4H)
1.00
1.02
5.14
4.03
4.18
3.17
4.13
2.10
4.12
ppm

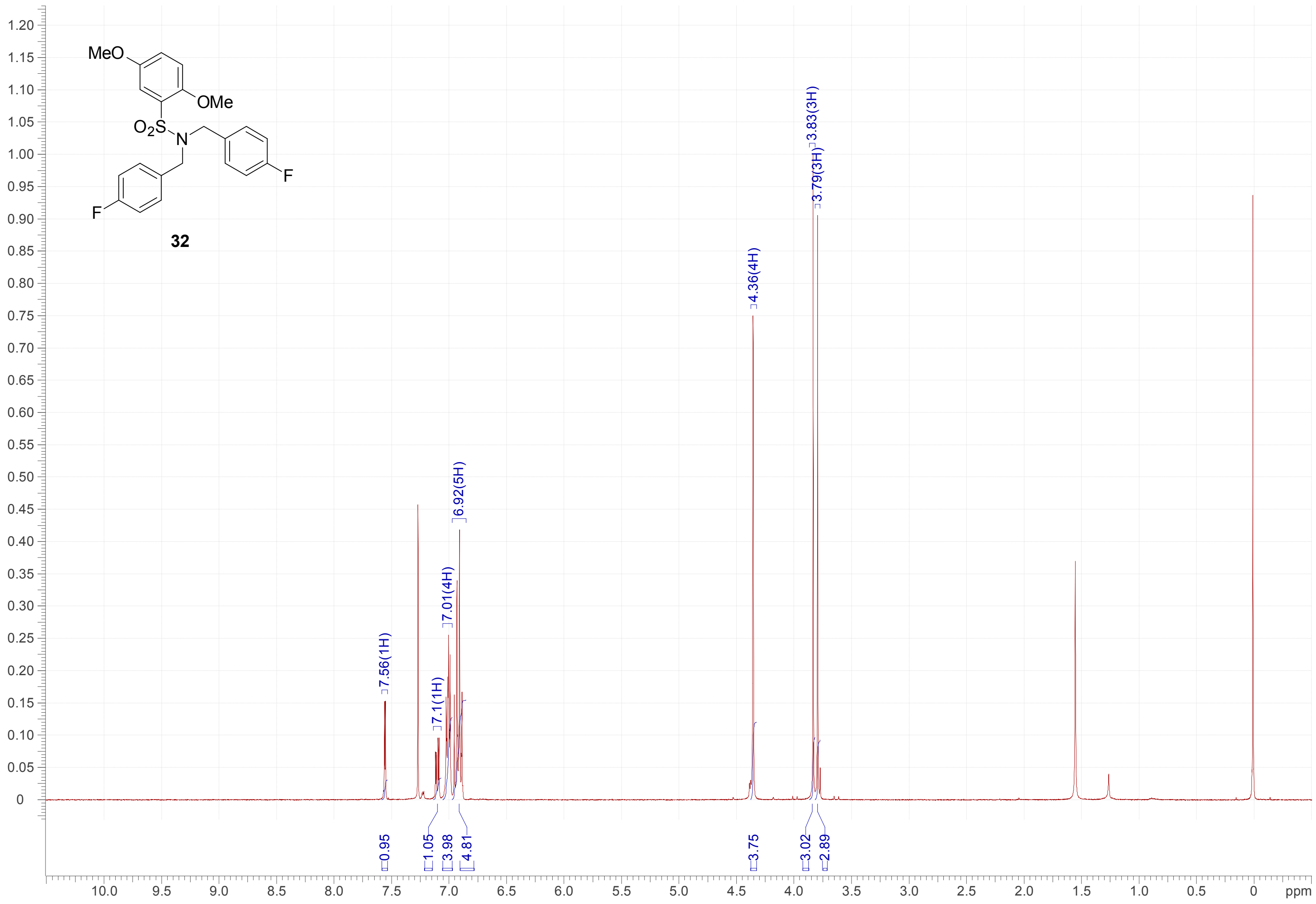
MeO
OMe
O2S
N
F
F
32
7.56(1H)
7.1(1H)
7.01(4H)
6.92(5H)
4.36(4H)
3.79(3H)
3.83(3H)
0.95
1.05
3.98
4.81
3.75
3.02
2.89
ppm

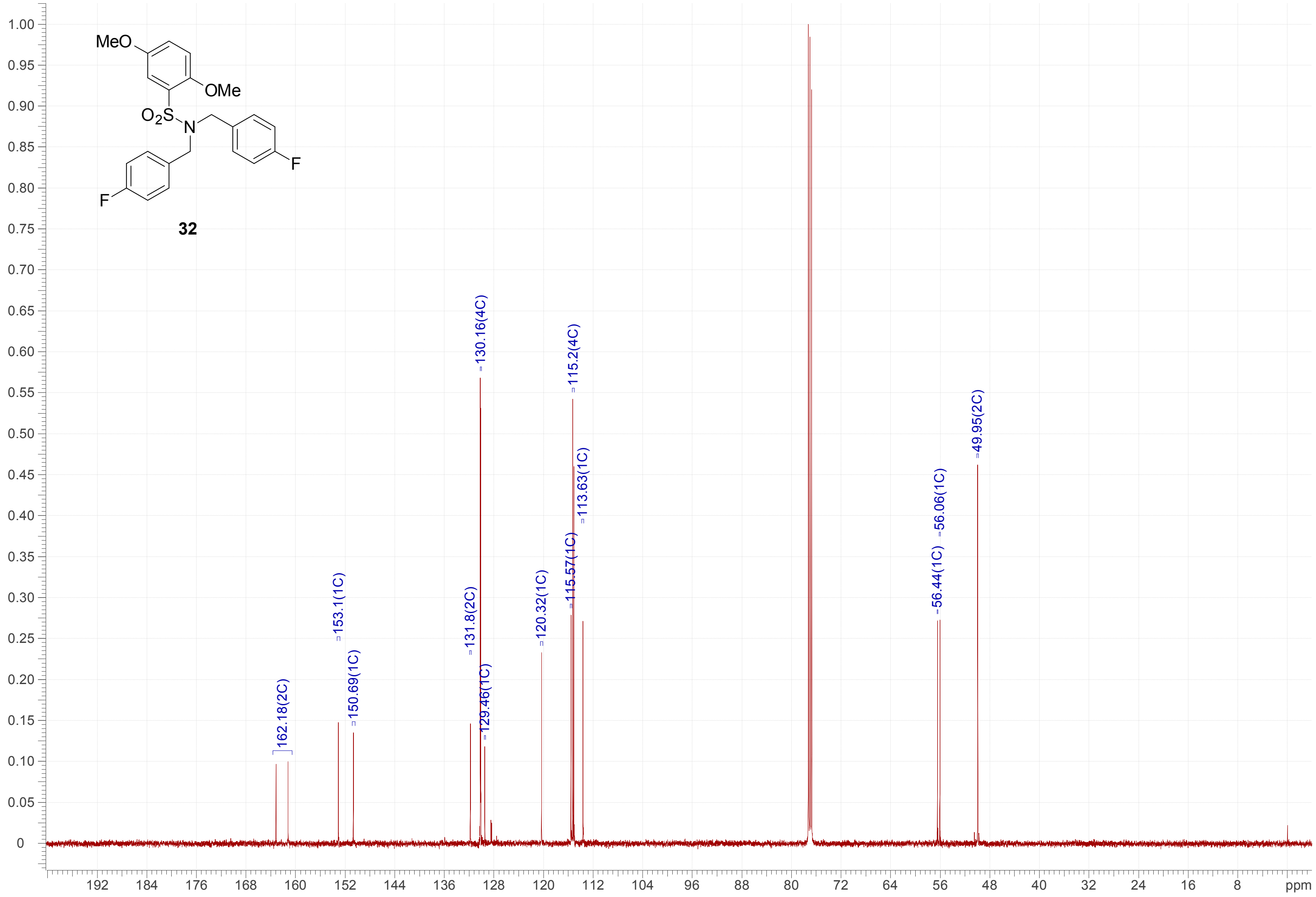
MeO
OMe
O2S
N
F
F
32
162.18(2C)
153.1(1C)
150.69(1C)
131.8(2C)
130.16(4C)
129.46(1C)
120.32(1C)
115.57(1C)
115.2(4C)
113.63(1C)
56.44(1C)
56.06(1C)
49.95(2C)
ppm

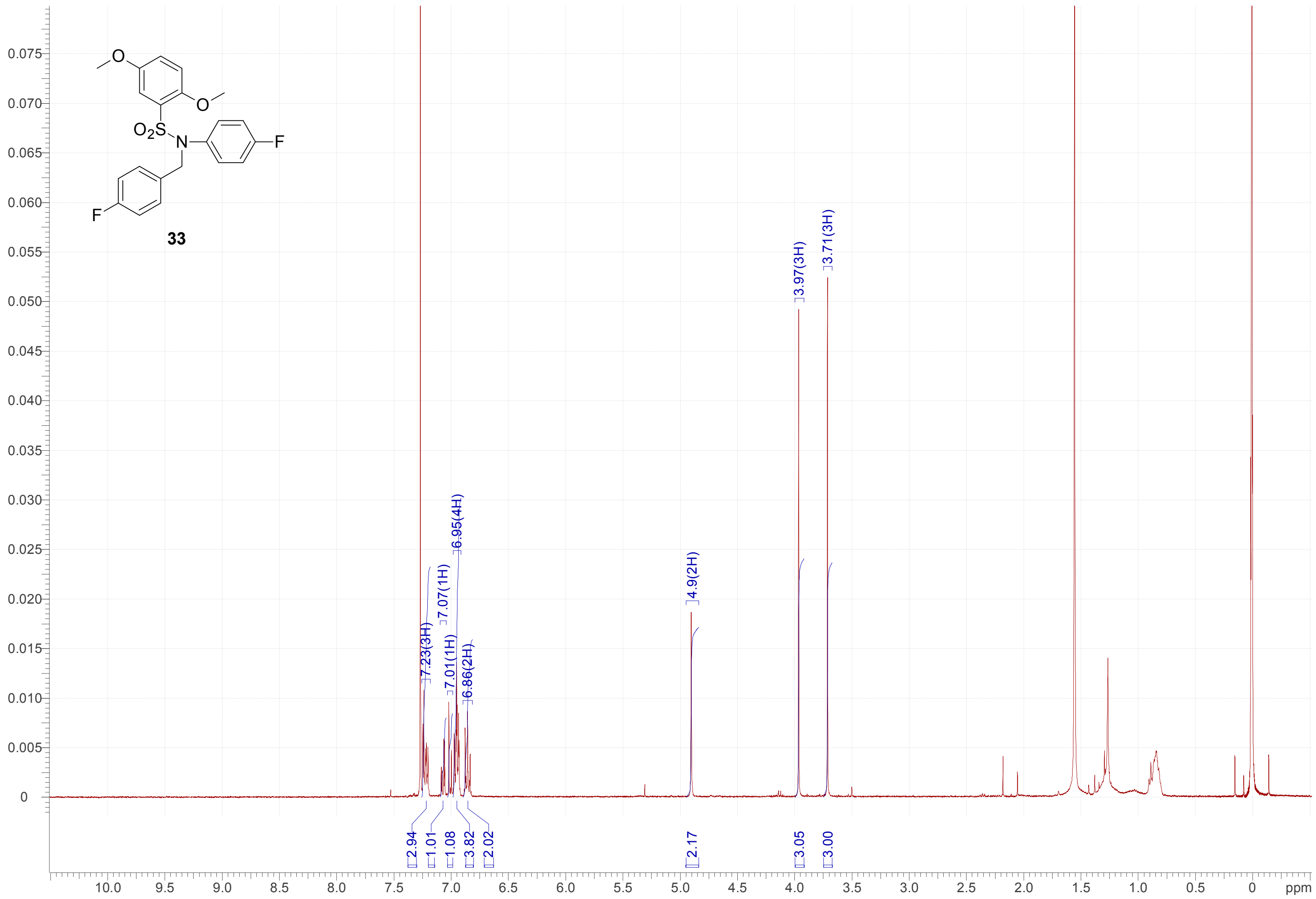

O2S
N
F
F
O
O
33
7.23(3H)
7.07(1H)
7.01(1H)
6.95(4H)
6.86(2H)
4.9(2H)
3.97(3H)
3.71(3H)
2.94
1.01
1.08
3.82
2.02
2.17
3.05
3.00
ppm

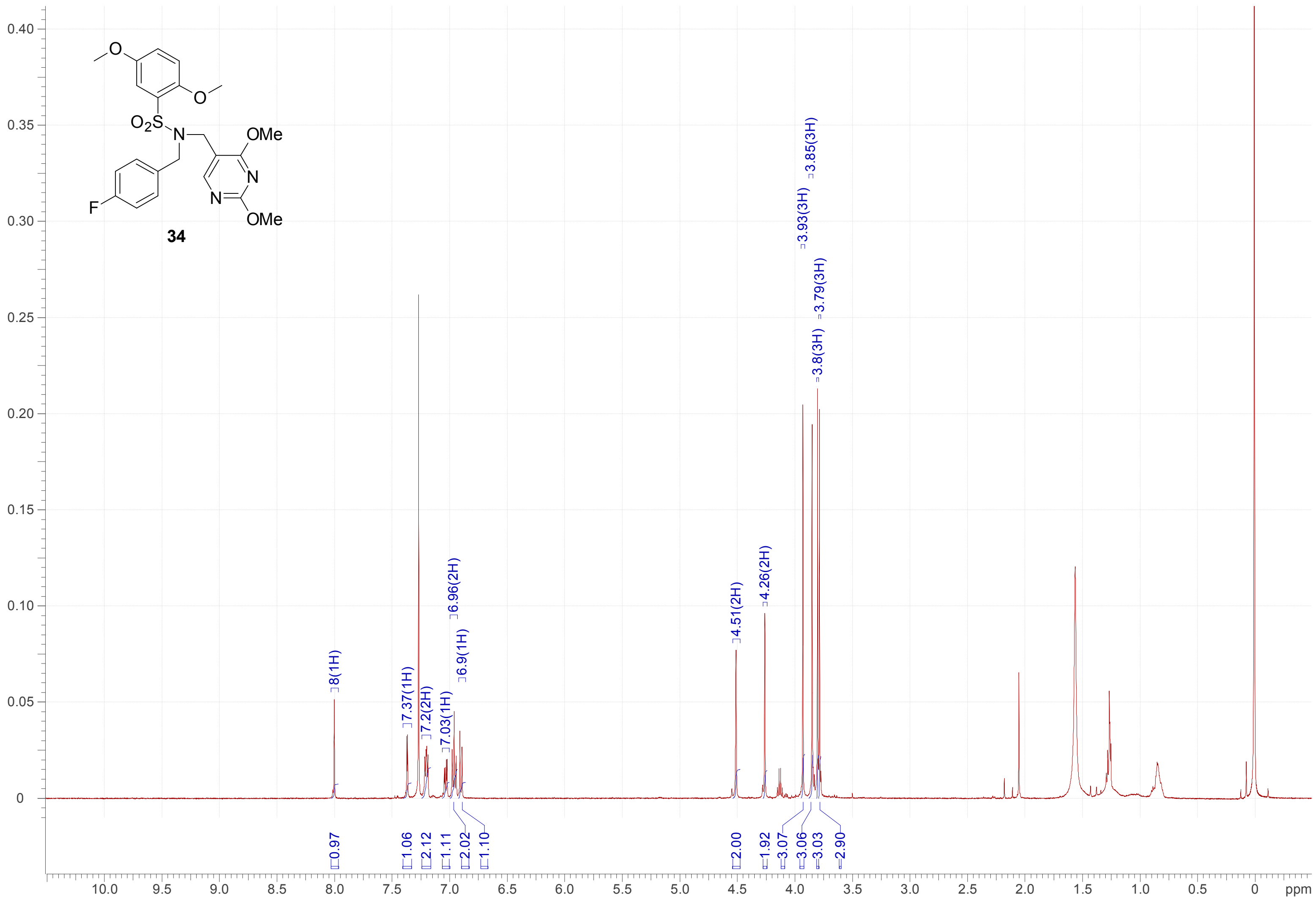
OMe
OMe
34
8(1H)
7.37(1H)
7.2(2H)
7.03(1H)
6.96(2H)
6.9(1H)
4.51(2H)
4.26(2H)
3.93(3H)
3.85(3H)
3.8(3H)
3.79(3H)
0.97
1.06
2.12
1.11
2.02
1.10
2.00
1.92
3.07
3.06
3.03
2.90
ppm

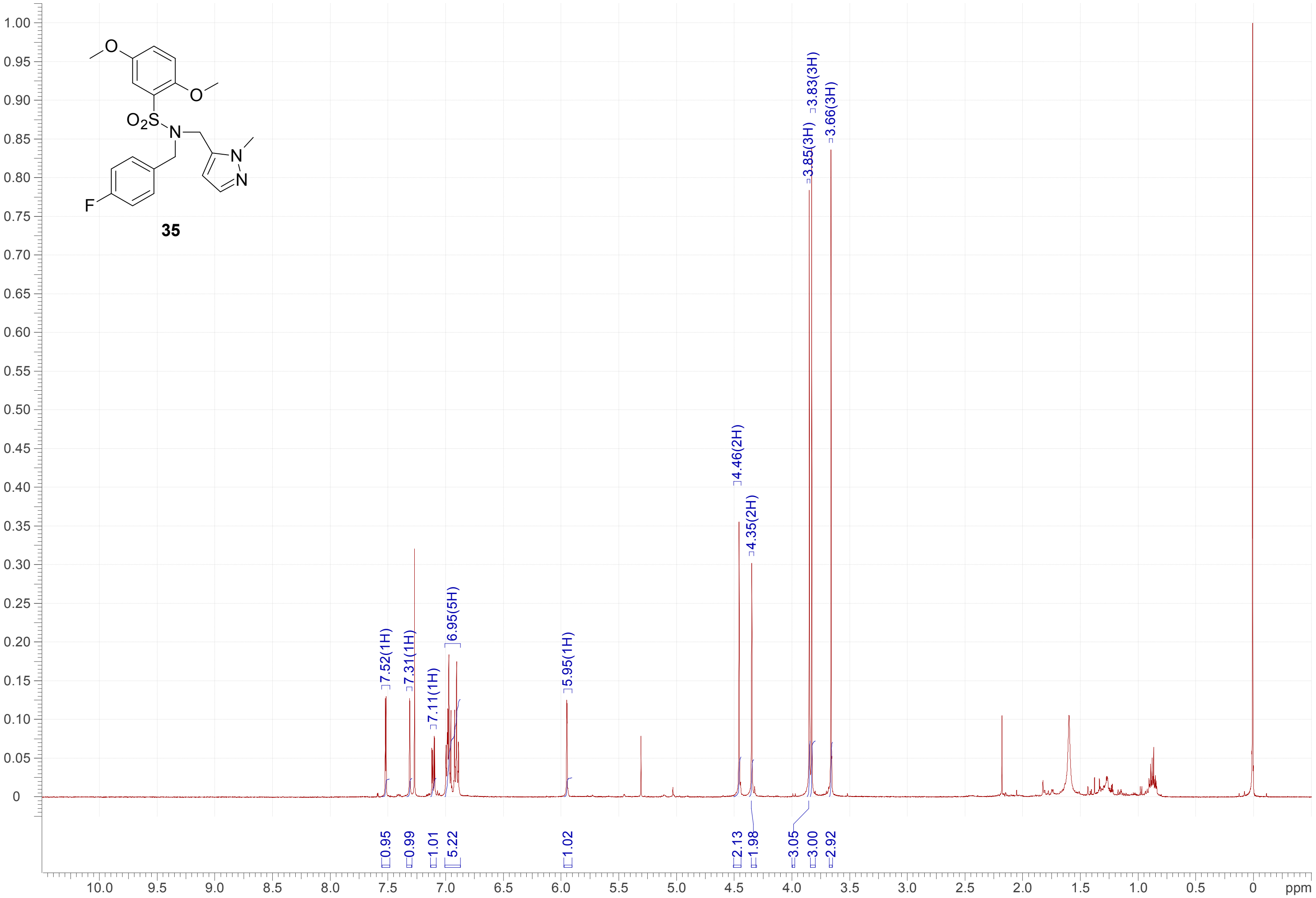
35
7.52(1H)
7.31(1H)
7.11(1H)
6.95(5H)
5.95(1H)
4.46(2H)
4.35(2H)
3.85(3H)
3.83(3H)
3.66(3H)
0.95
0.99
1.01
5.22
1.02
2.13
1.98
3.05
3.00
2.92
ppm

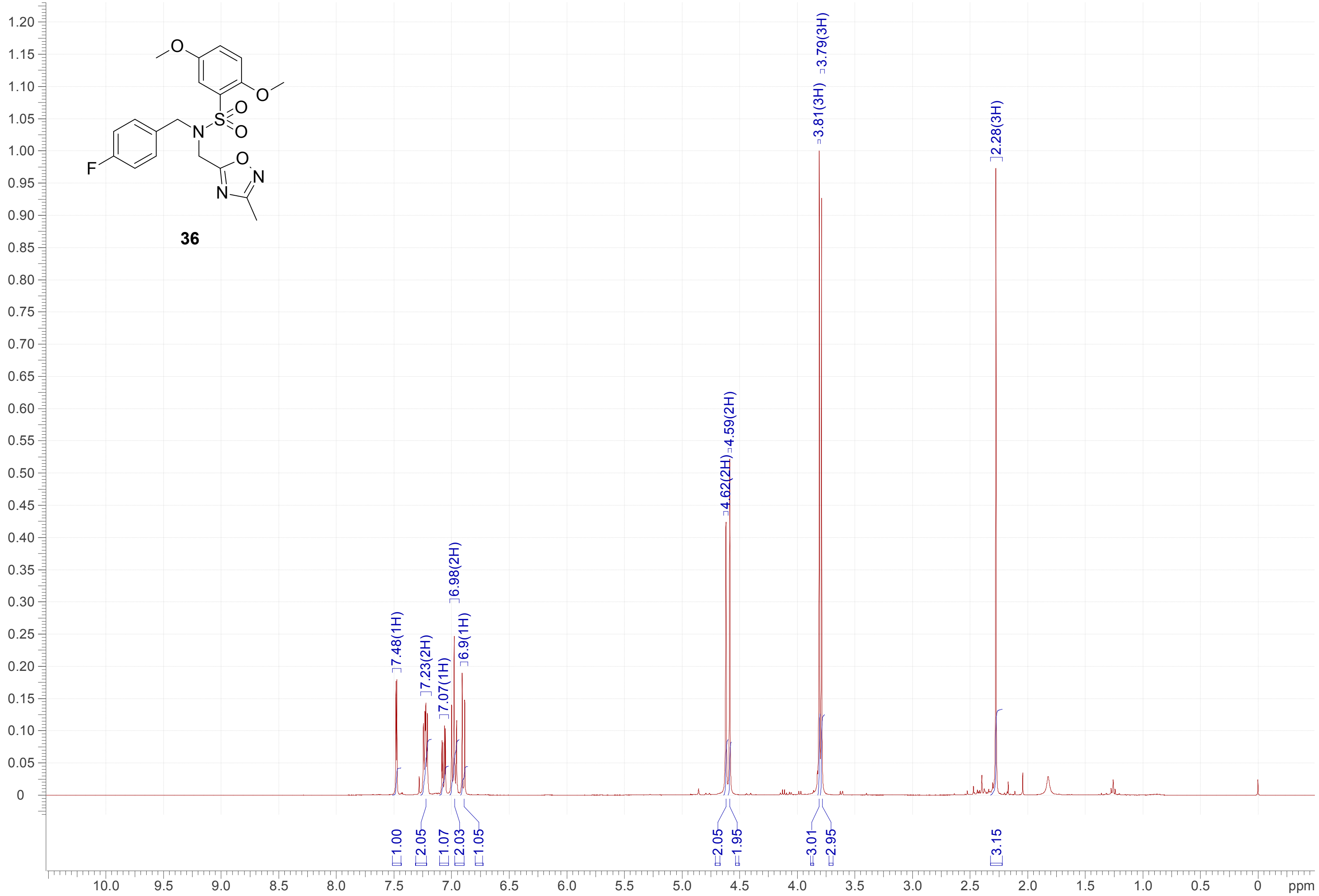
36
7.48(1H)
7.23(2H)
7.07(1H)
6.98(2H)
6.9(1H)
4.62(2H)
4.59(2H)
3.81(3H)
3.79(3H)
2.28(3H)
1.00
2.05
1.07
2.03
1.05
2.05
1.95
3.01
2.95
3.15
ppm

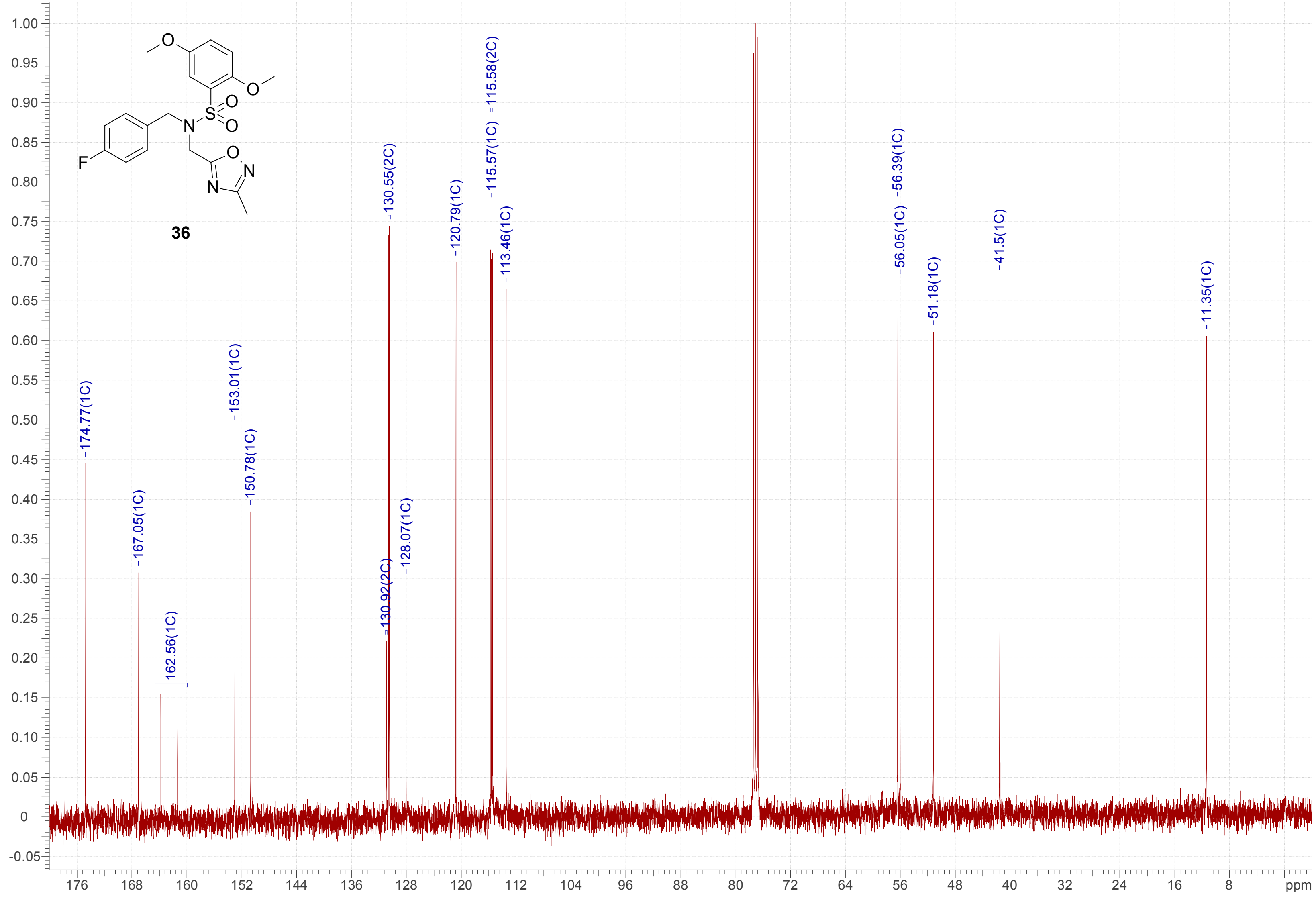

36
174.77(1C)
167.05(1C)
162.56(1C)
153.01(1C)
150.78(1C)
130.92(2C)
130.55(2C)
128.07(1C)
120.79(1C)
115.58(2C)
115.57(1C)
113.46(1C)
56.39(1C)
56.05(1C)
51.18(1C)
41.5(1C)
11.35(1C)
ppm

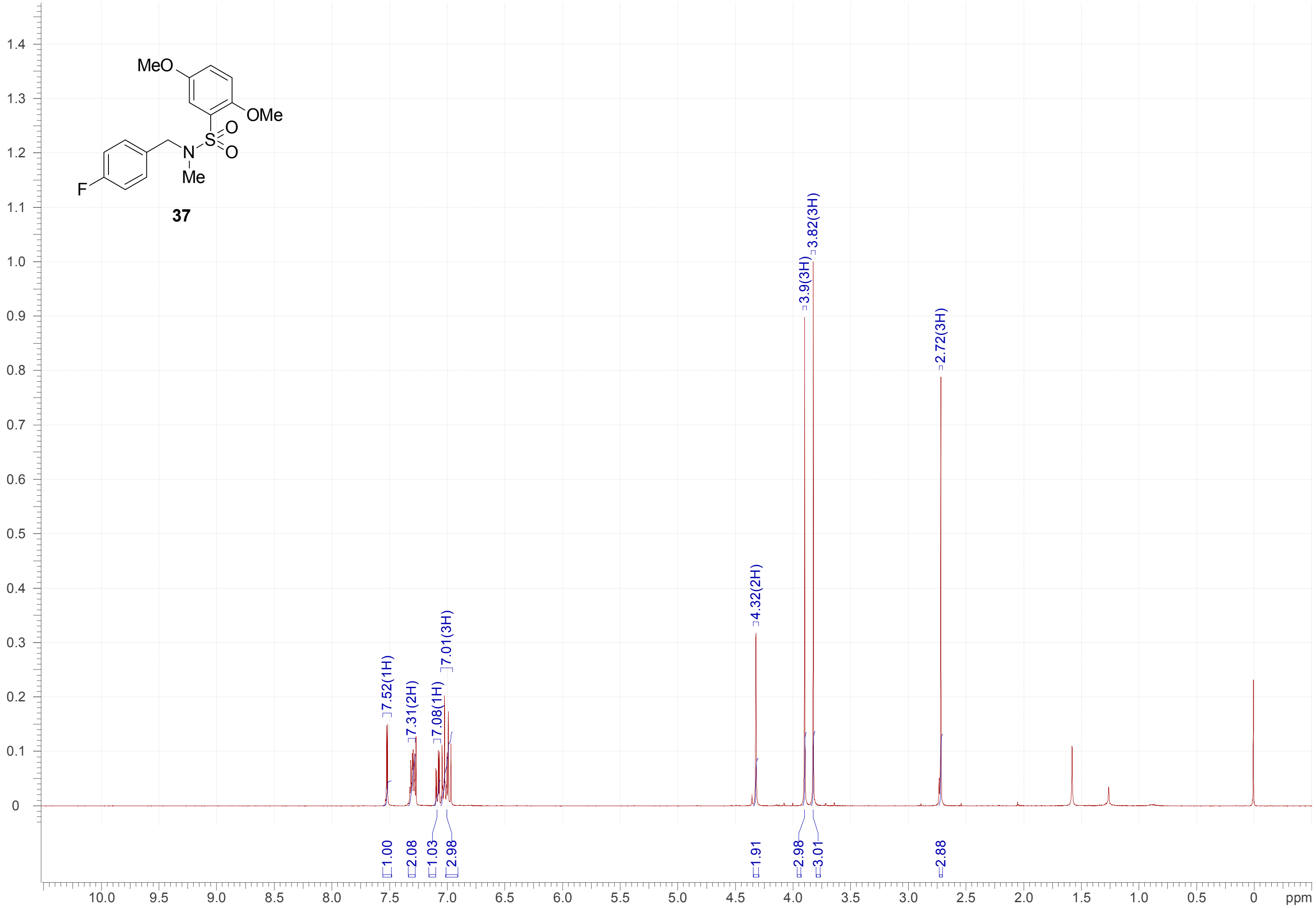
MeO
OMe
S
O
O
N
Me
F
37
7.52(1H)
7.31(2H)
7.08(1H)
7.01(3H)
4.32(2H)
3.9(3H)
3.82(3H)
2.72(3H)
1.00
2.08
1.03
2.98
1.91
2.98
3.01
2.88
ppm

mad1629 cdcl3

Acquisition Time (sec)	2.0447	***Comment***	mad1629 cdcl3	***Date***	11 Sep 2017 09:14:30		
File Name	\\nmrsparc.iqm.unicamp.br\espectros\avance400\2017\set17\Reserva\Luiz Carlos\set09madH1_001001r					***Frequency (MHz)***	400.18
Nucleus	1H	***Number of Transients***	8	***Original Points Count***	16384	***Points Count***	65536
Pulse Sequence	zg30	***Solvent***	CHLOROFORM-D	***Sweep Width (Hz)***	8012.82	***Temperature (degree C)***	24.170

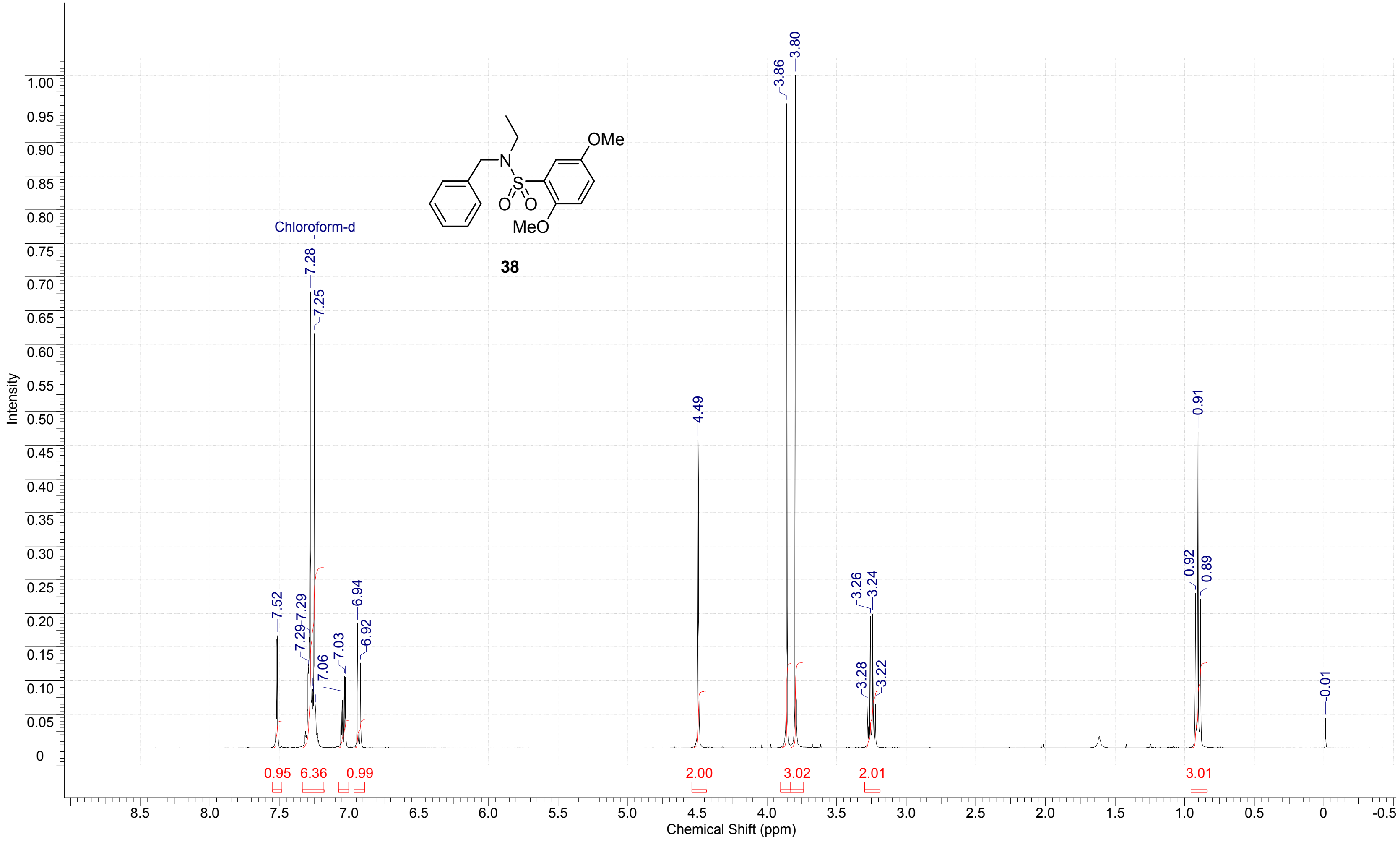

**Acquisition Time (sec)**	0.6816	**Comment**	Dessoy - MAD 1629 - CDCl3 - Avance 400 MHz - set12madC1 - 13C 2 horas			**Date**	13 Sep 2017 07:13:12
**File Name**	\\nmrsparc.iqm.unicamp.br\espectros\avance400\2017\set17\Sala\Luiz Carlos\set12madC1_001001r					**Frequency (MHz)**	100.63
**Nucleus**	13C	**Number of Transients**	4096	**Original Points Count**	16384	**Points Count**	65536
**Pulse Sequence**	zgpg30	**Solvent**	CHLOROFORM-D	**Sweep Width (Hz)**	24038.46	**Temperature (degree C)**	25.042

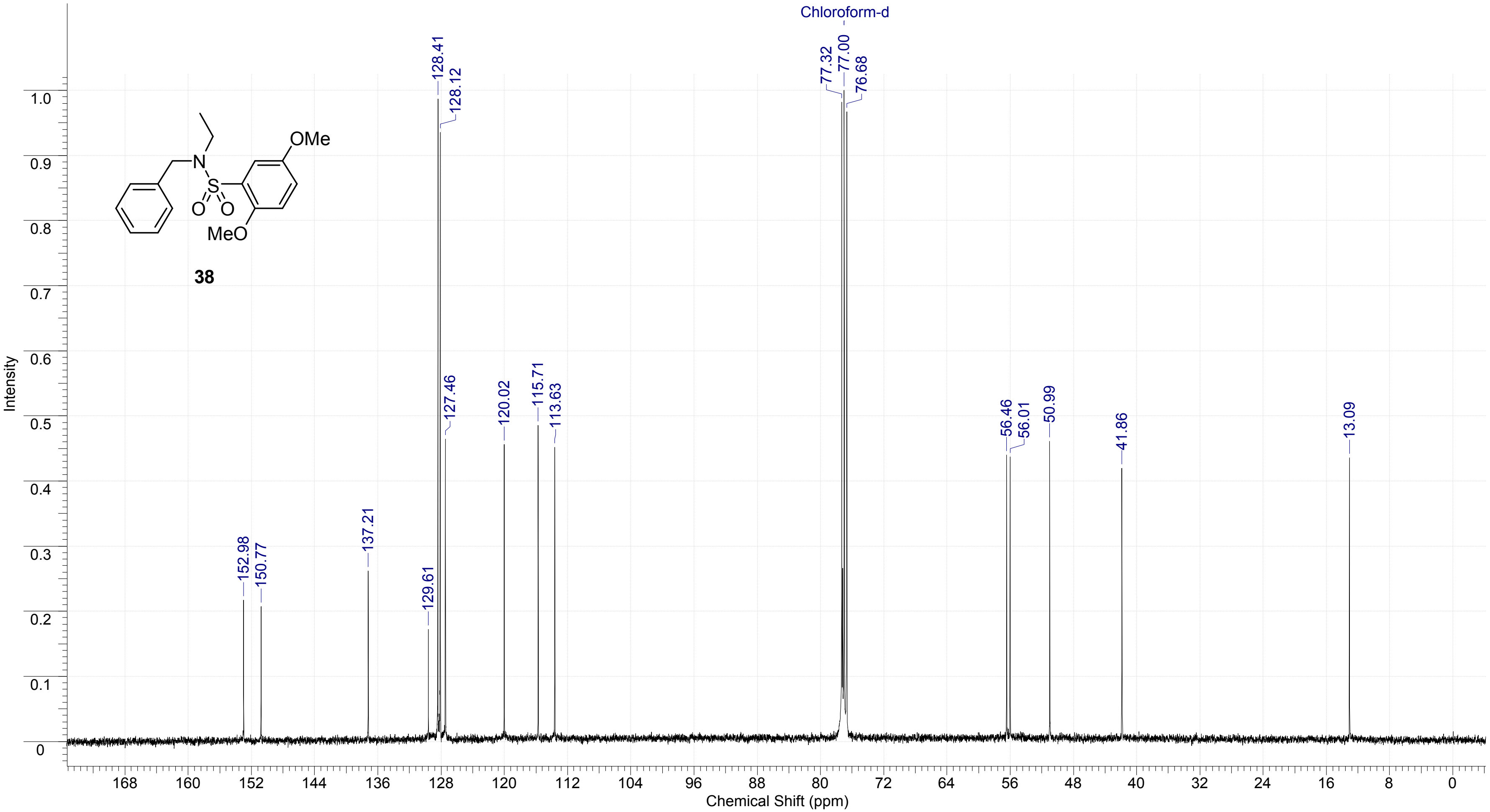

DESSOY MAD1632 CDCl3 400 MHz set19madH1

Acquisition Time (sec)	2.0447	***Comment***	DESSOY MAD1632 CDCl3 400 MHz set19madH1			***Date***	19 Sep 2017 11:28:32
File Name	\\nmrsparc.iqm.unicamp.br\espectros\avance400\2017\set17\Reserva\Luiz Carlos\set19madH1_001001r					***Frequency (MHz)***	400.18
Nucleus	1H	***Number of Transients***	16	***Original Points Count***	16384	***Points Count***	65536
Pulse Sequence	zg30	***Solvent***	CHLOROFORM-D	***Sweep Width (Hz)***	8012.82	***Temperature (degree C)***	25.292

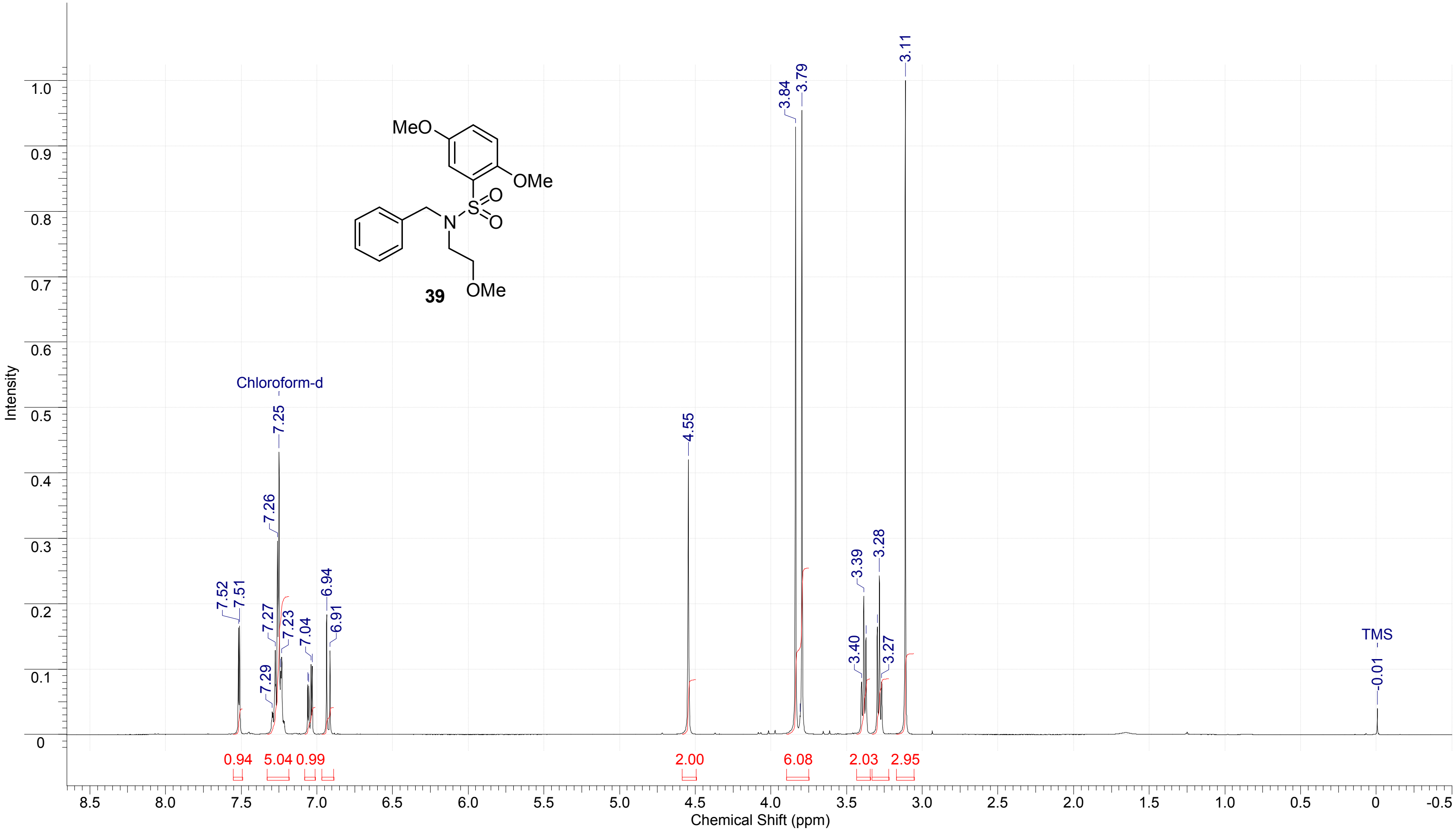

Dessoy - MAD 1632 - CDCl3 - Avance 400 MHz - set19madC1 - 13C 1h 30min

Acquisition Time (sec)	0.6816	***Comment***	Dessoy - MAD 1632 - CDCl3 - Avance 400 MHz - set19madC1 - 13C 1h 30min			***Date***	20 Sep 2017 07:07:00
File Name	\\nmrsparc.iqm.unicamp.br\espectros\avance400\2017\set17\Sala\Luiz Carlos\set19madC1_001001r					***Frequency (MHz)***	100.63
Nucleus	13C	***Number of Transients***	3072	***Original Points Count***	16384	***Points Count***	65536
Pulse Sequence	zgpg30	***Solvent***	CHLOROFORM-D	***Sweep Width (Hz)***	24038.46	***Temperature (degree C)***	25.167

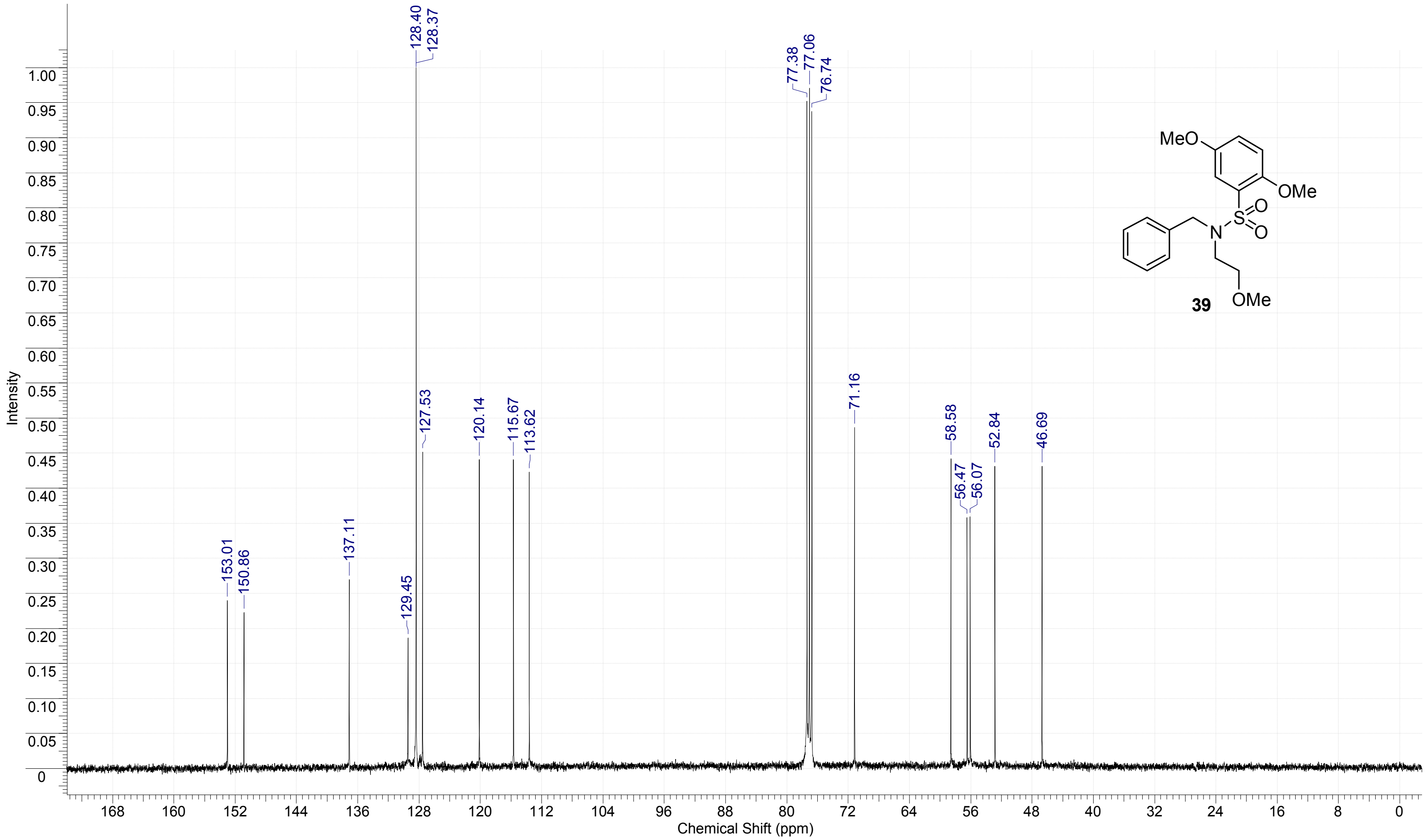

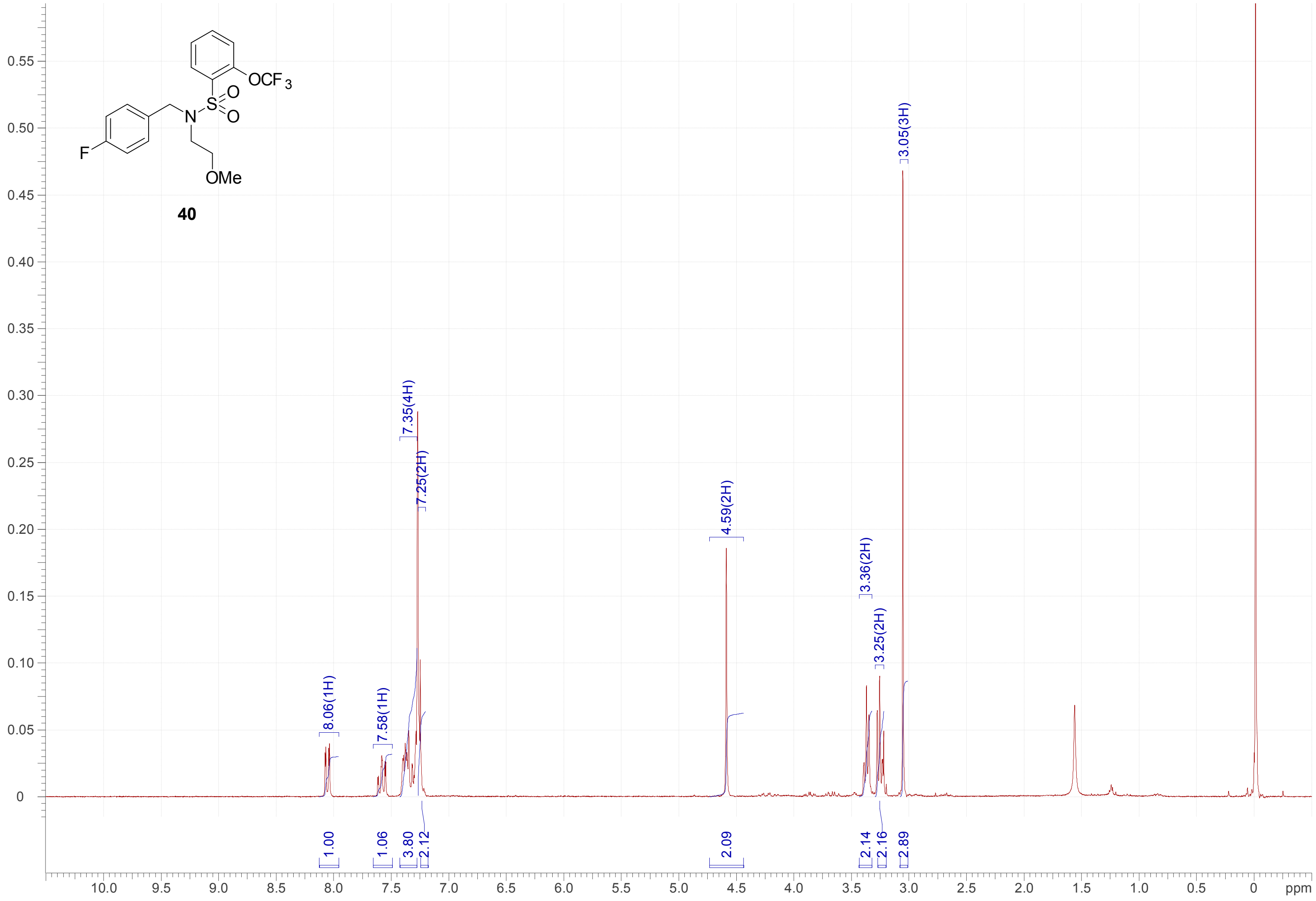

OCF3
S
O
O
N
F
OMe
40
8.06(1H)
7.58(1H)
7.35(4H)
7.25(2H)
4.59(2H)
3.36(2H)
3.25(2H)
3.05(3H)
1.00
1.06
3.80
2.12
2.09
2.14
2.16
2.89
0.55
0.50
0.45
0.40
0.35
0.30
0.25
0.20
0.15
0.10
0.05
0
10.0 9.5 9.0 8.5 8.0 7.5 7.0 6.5 6.0 5.5 5.0 4.5 4.0 3.5 3.0 2.5 2.0 1.5 1.0 0.5 0 ppm

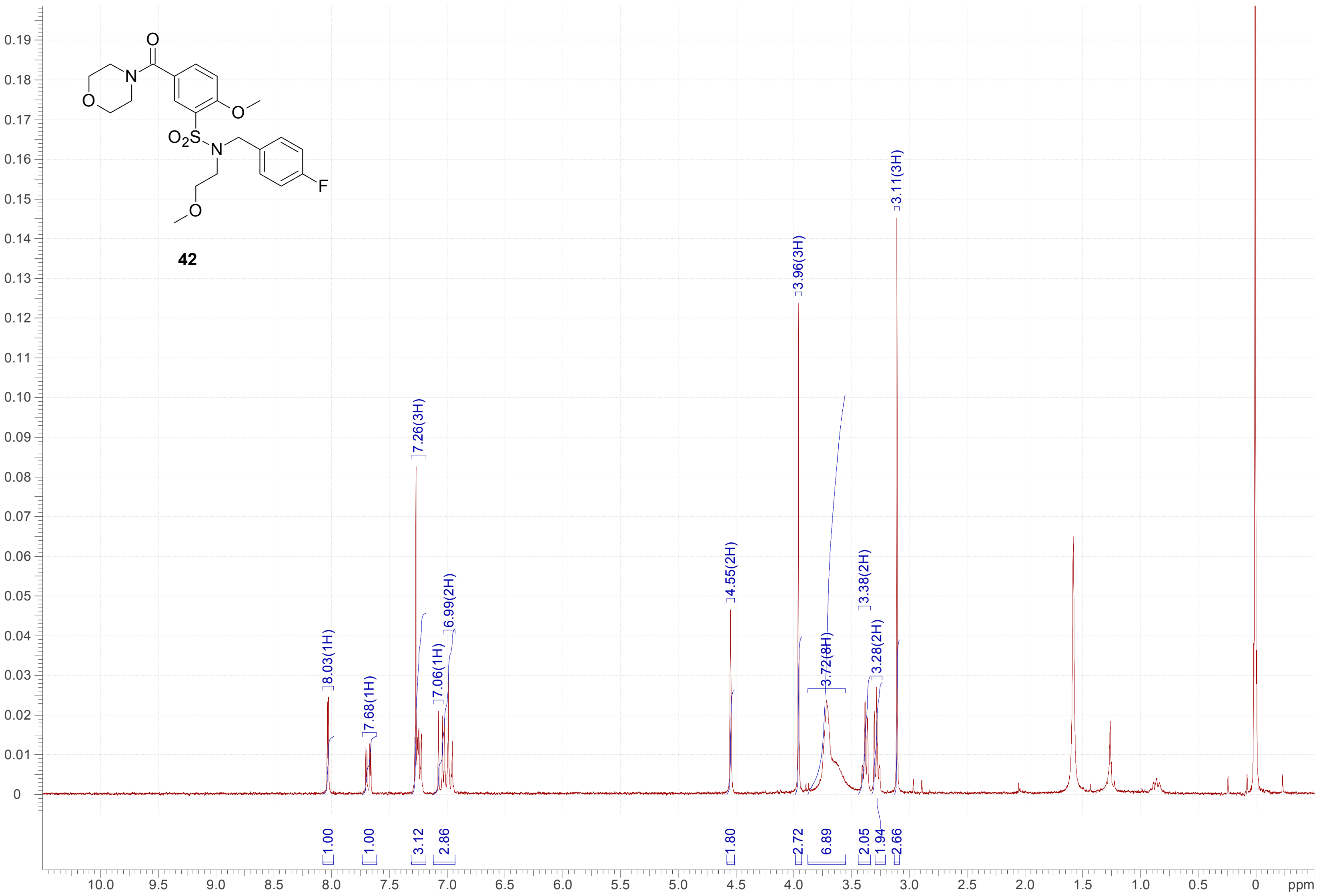

42
8.03(1H)
7.68(1H)
7.26(3H)
7.06(1H)
6.99(2H)
4.55(2H)
3.96(3H)
3.72(8H)
3.38(2H)
3.28(2H)
3.11(3H)
1.00
1.00
3.12
2.86
1.80
2.72
6.89
2.05
1.94
2.66
ppm

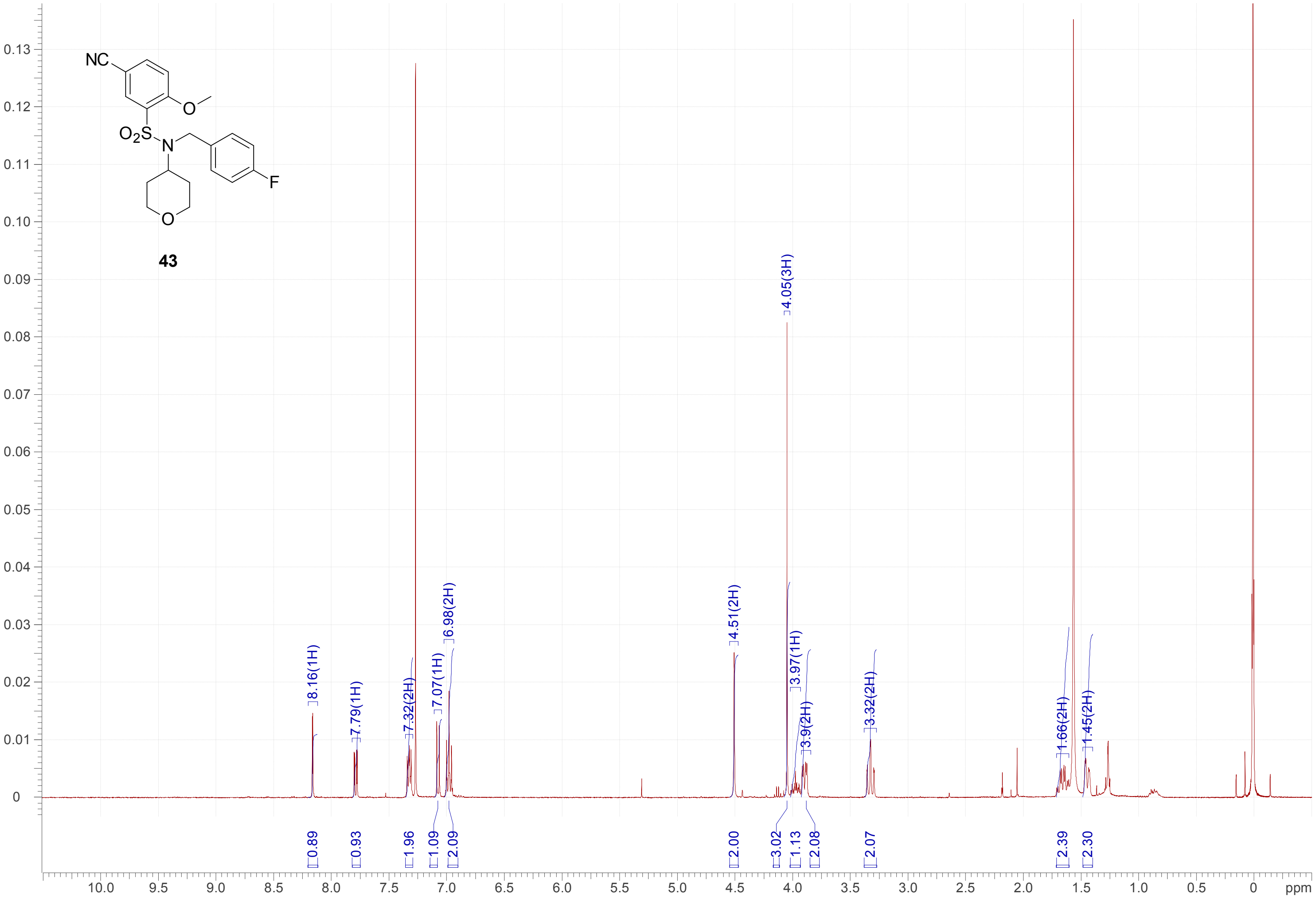
NC
O
O2S
N
F
O
43
8.16(1H)
7.79(1H)
7.32(2H)
7.07(1H)
6.98(2H)
4.51(2H)
4.05(3H)
3.97(1H)
3.9(2H)
3.32(2H)
1.66(2H)
1.45(2H)
0.89
0.93
1.96
1.09
2.09
2.00
3.02
1.13
2.08
2.07
2.39
2.30
ppm

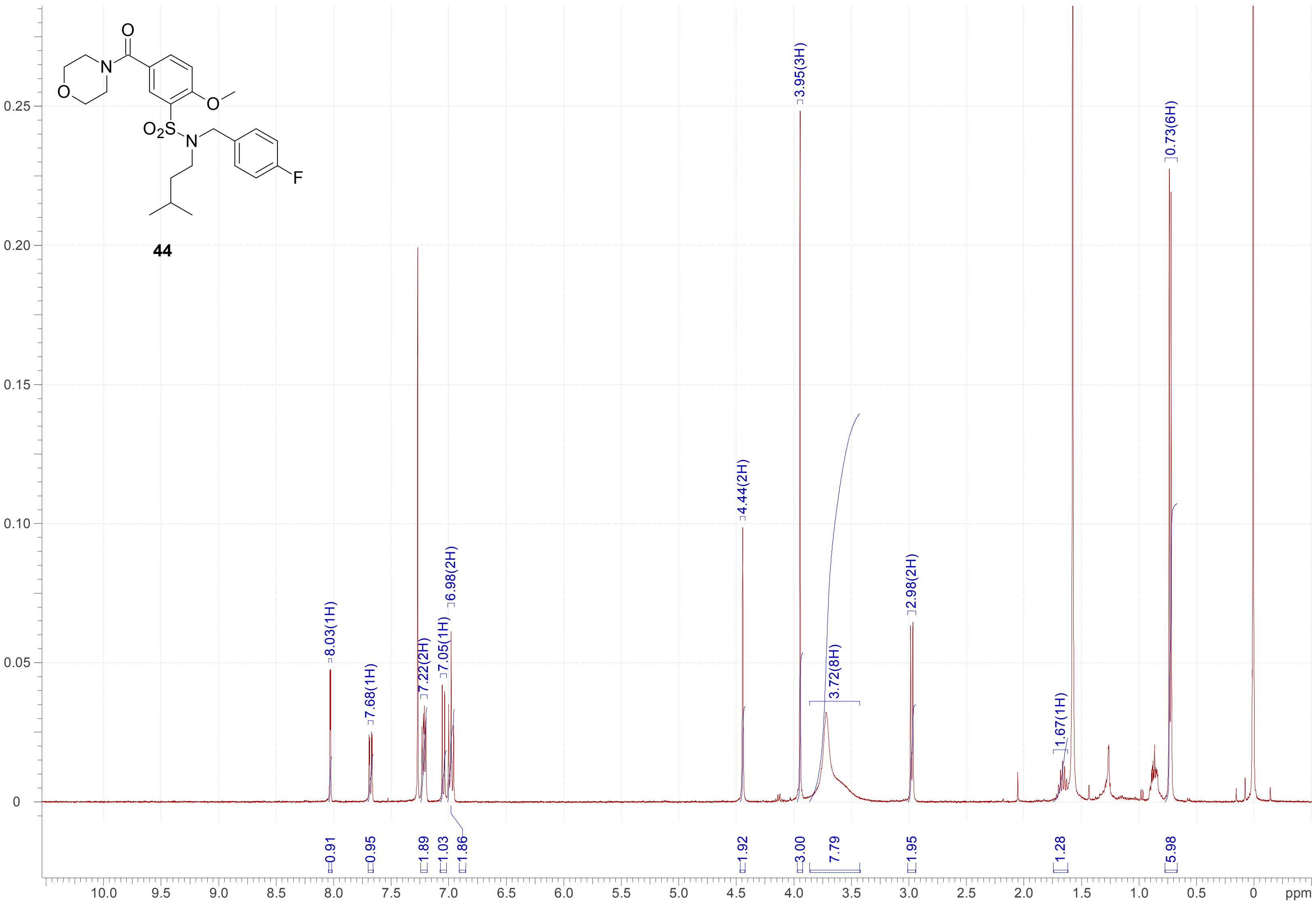
44
8.03(1H)
7.68(1H)
7.22(2H)
7.05(1H)
6.98(2H)
4.44(2H)
3.95(3H)
3.72(8H)
2.98(2H)
1.67(1H)
0.73(6H)
0.91
0.95
1.89
1.03
1.86
1.92
3.00
7.79
1.95
1.28
5.98
ppm

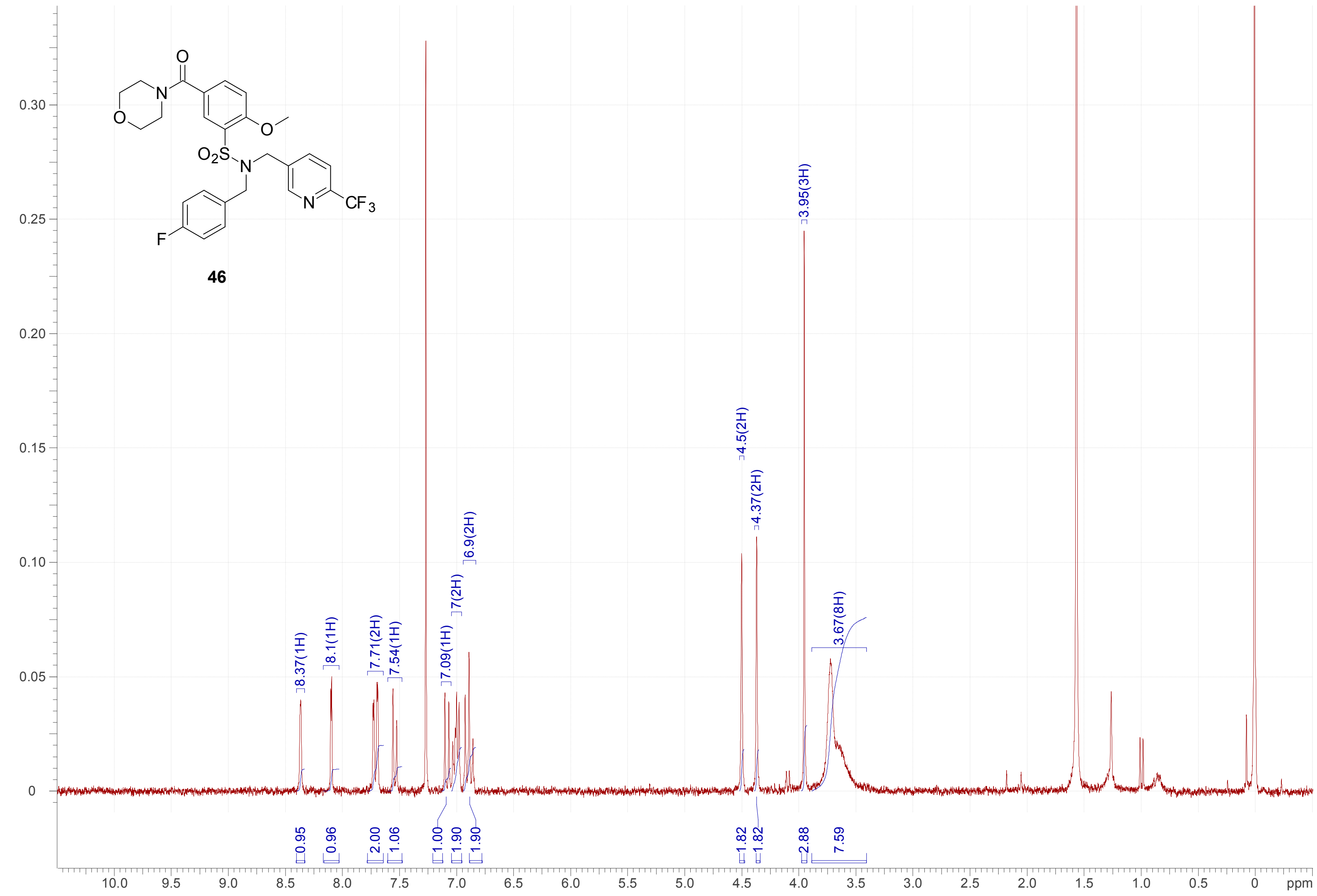
46
O
N
O
O
O_2S
N
F
N
CF_3
8.37(1H)
8.1(1H)
7.71(2H)
7.54(1H)
7.09(1H)
7(2H)
6.9(2H)
4.5(2H)
4.37(2H)
3.95(3H)
3.67(8H)
0.95
0.96
2.00
1.06
1.00
1.90
1.90
1.82
1.82
2.88
7.59
0.30
0.25
0.20
0.15
0.10
0.05
0
10.0 9.5 9.0 8.5 8.0 7.5 7.0 6.5 6.0 5.5 5.0 4.5 4.0 3.5 3.0 2.5 2.0 1.5 1.0 0.5 0 ppm

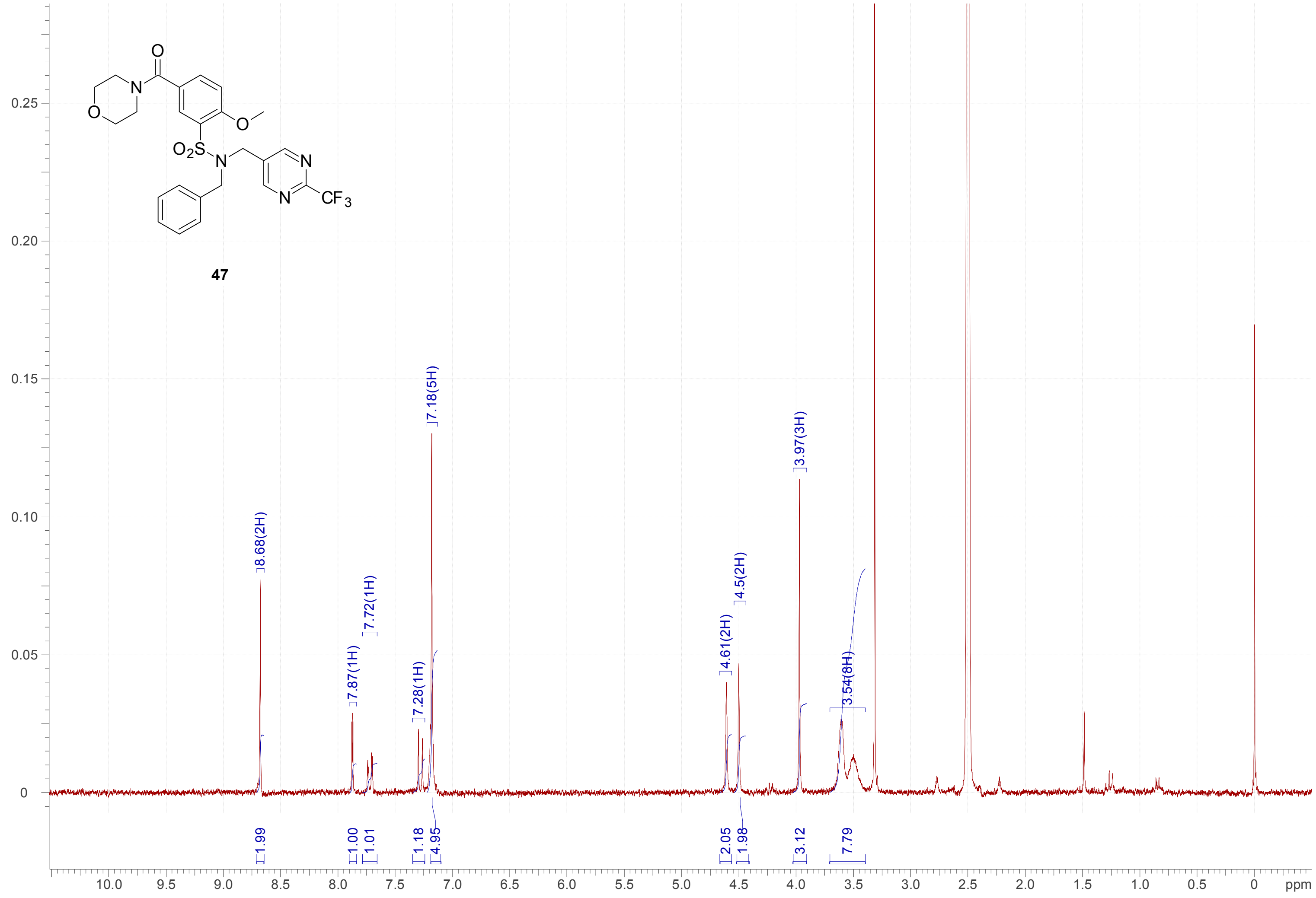

8.68(2H)
7.87(1H)
7.72(1H)
7.28(1H)
7.18(5H)
4.61(2H)
4.5(2H)
3.97(3H)
3.54(8H)
1.99
1.00
1.01
1.18
4.95
2.05
1.98
3.12
7.79
ppm

47

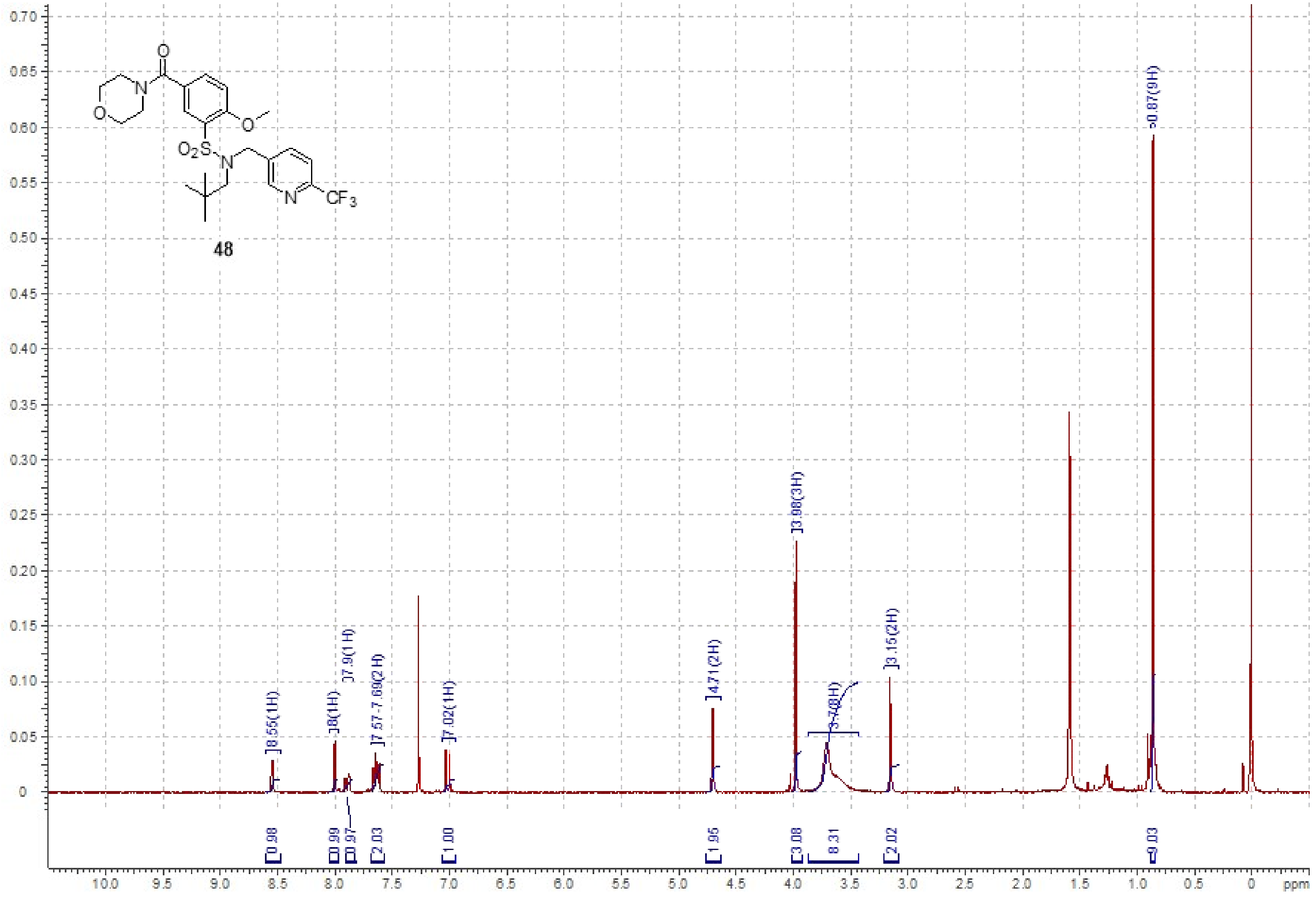
O
N
O
O
O_2S
N
N
CF_3
48
8.55(1H)
8(1H)
7.9(1H)
7.57-7.69(2H)
7.02(1H)
4.71(2H)
3.98(3H)
3.7(8H)
3.15(2H)
0.87(9H)
0.98
0.99
0.97
2.03
1.00
1.95
3.08
8.31
2.02
9.03
0.70
0.65
0.60
0.55
0.50
0.45
0.40
0.35
0.30
0.25
0.20
0.15
0.10
0.05
0
10.0
9.5
9.0
8.5
8.0
7.5
7.0
6.5
6.0
5.5
5.0
4.5
4.0
3.5
3.0
2.5
2.0
1.5
1.0
0.5
0
ppm

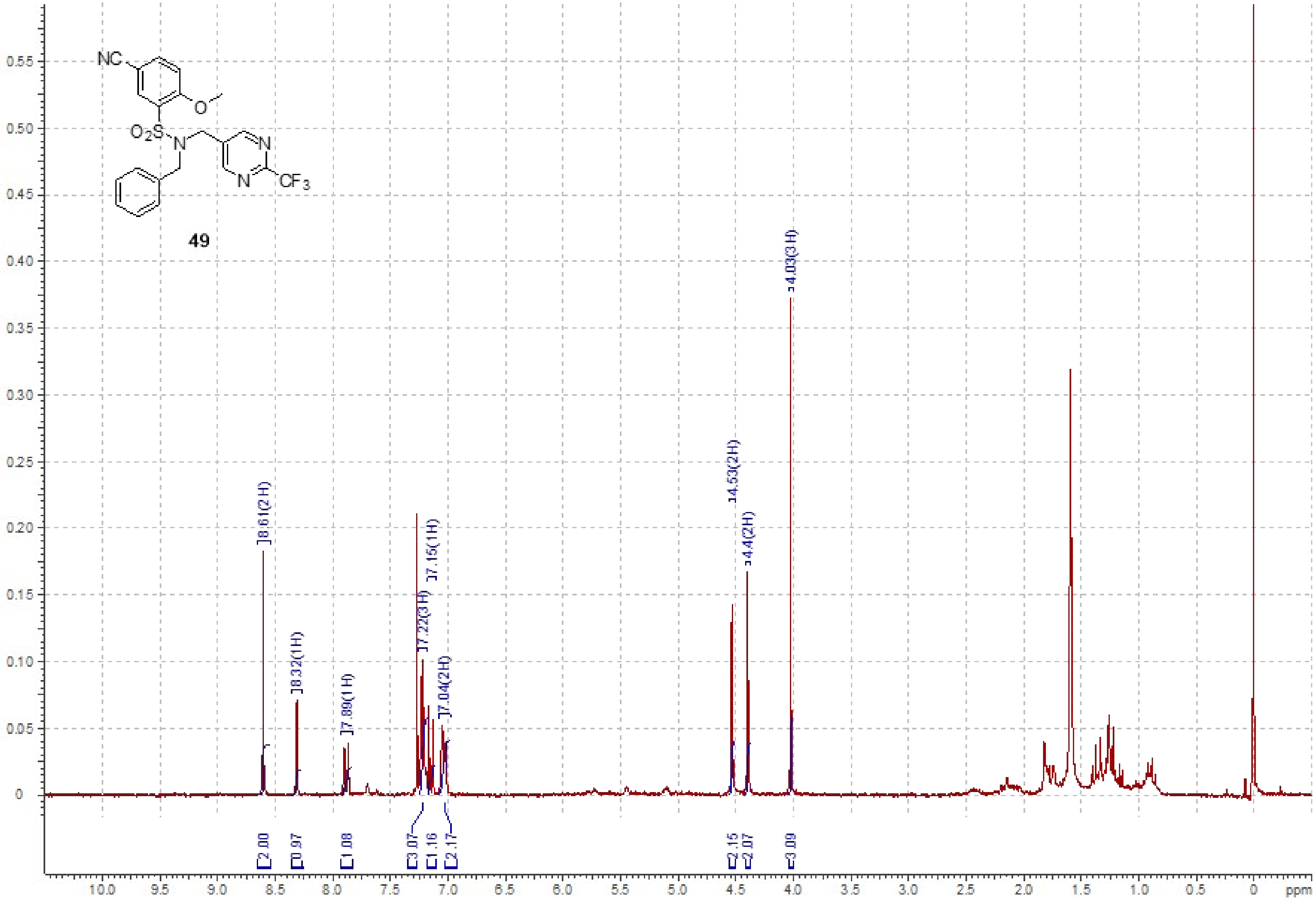

NC
O
O2S
N
N
N
CF3
49
8.61(2H)
8.32(1H)
7.89(1H)
7.22(3H)
7.15(1H)
7.04(2H)
4.53(2H)
4.4(2H)
4.03(3H)
2.00
0.97
1.08
3.07
1.16
2.17
2.15
2.07
3.09
ppm

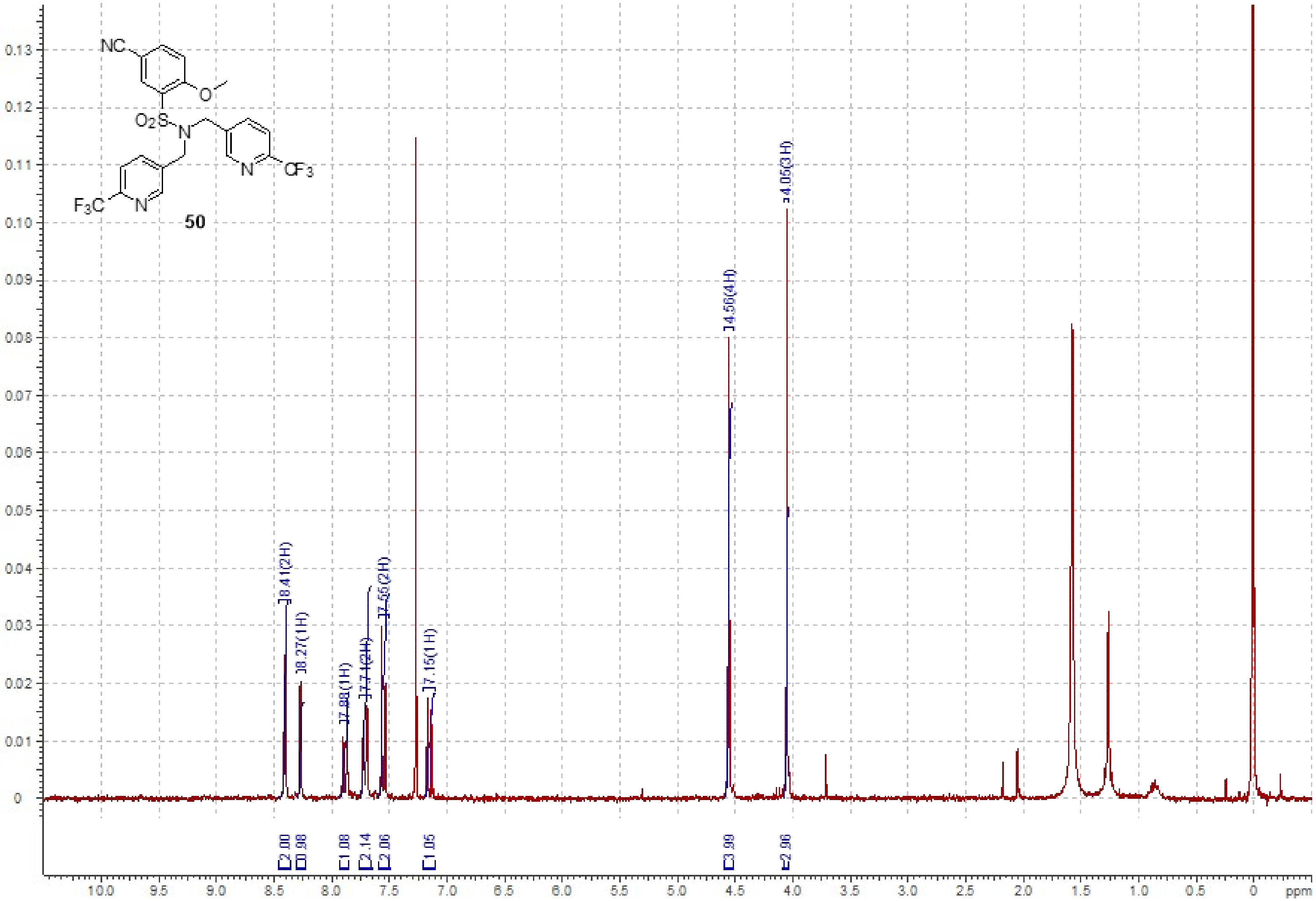
NC
O
O2S
N
N
CF3
F3C
N
50
8.41(2H)
8.27(1H)
7.88(1H)
7.71(2H)
7.55(2H)
7.15(1H)
4.56(4H)
4.05(3H)
2.00
0.98
1.08
2.14
2.06
1.05
3.99
2.96
ppm
